# Supplementary material for: Transcriptomic Changes in Cisplatin-Resistant MCF-7 Cells
Source: Int J Mol Sci. 2024 Mar 29;25(7):3820. doi: 10.3390/ijms25073820 (PMC11011657; doi:10.3390/ijms25073820)
Supplement: Supplementary file 1 [file ijms-25-03820-s001.zip › ijms-2687107-supplementary additions/cpR-MCF-7N3_star_trim_gcrh38_rsem.pdf]

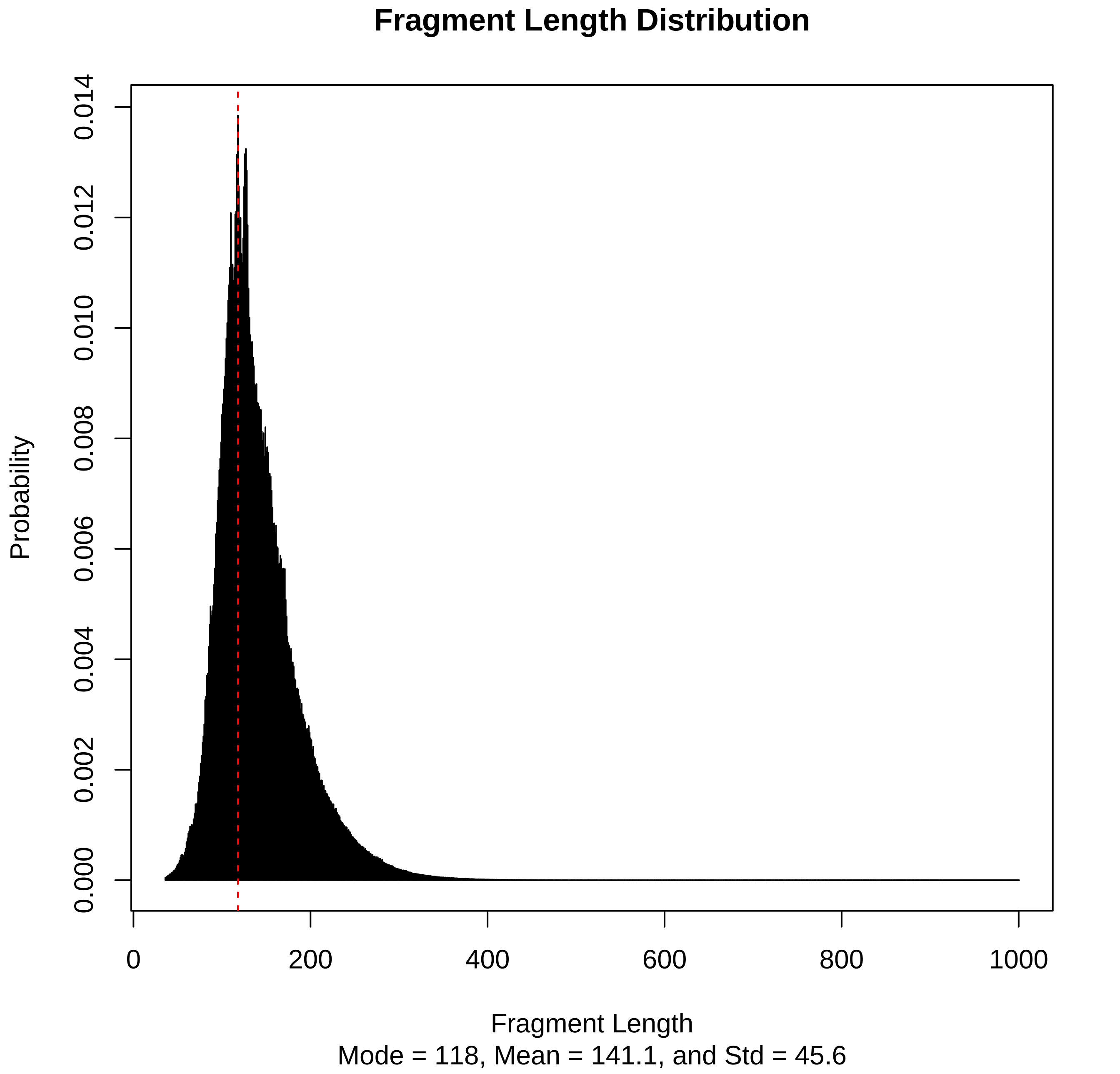

Fragment Length Distribution
Probability
0.000
0.002
0.004
0.006
0.008
0.010
0.012
0.014
0
200
400
600
800
1000
Fragment Length
Mode = 118, Mean = 141.1, and Std = 45.6

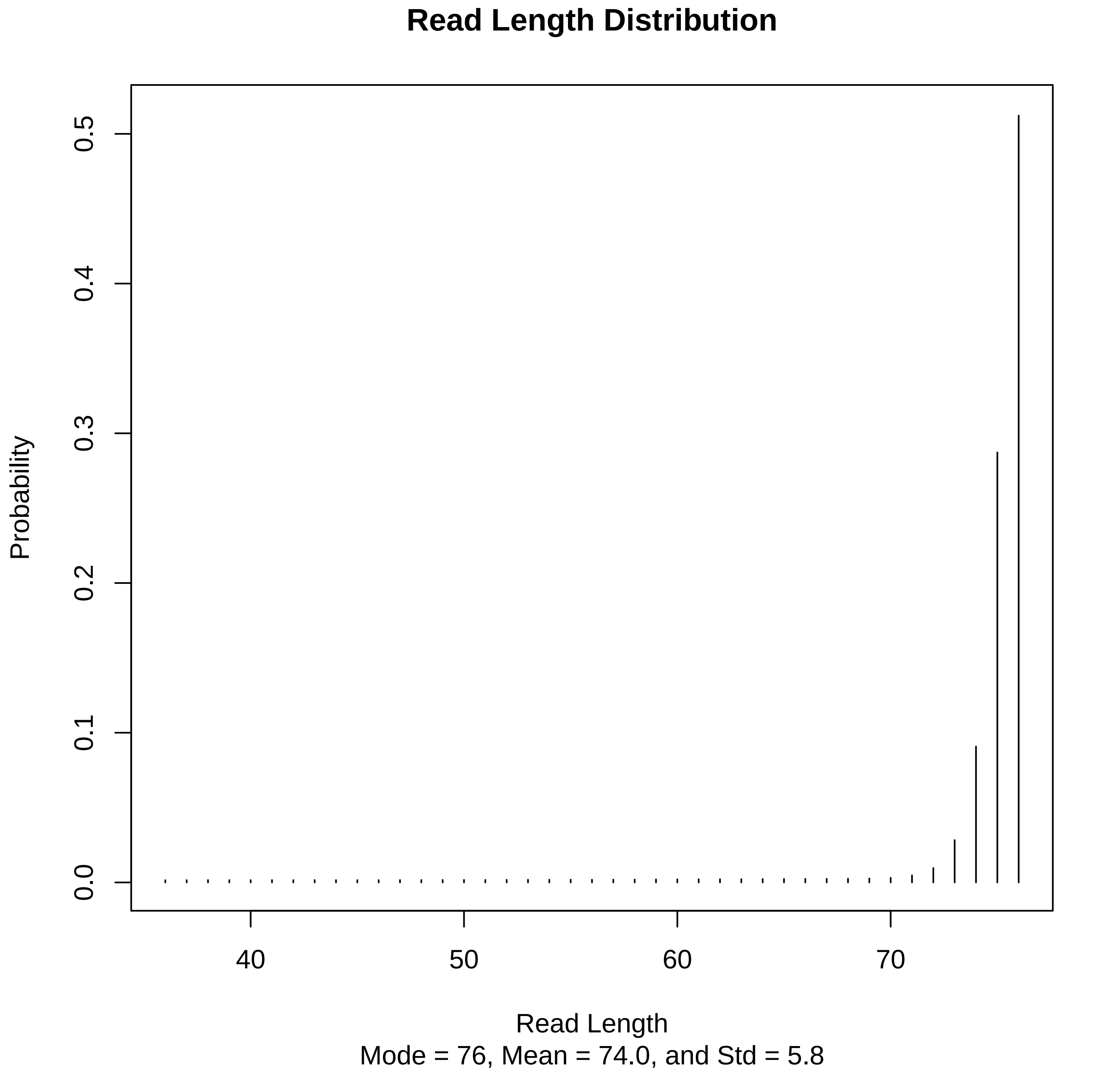
Read Length Distribution
Probability
0.0
0.1
0.2
0.3
0.4
0.5
40
50
60
70
Read Length
Mode = 76, Mean = 74.0, and Std = 5.8

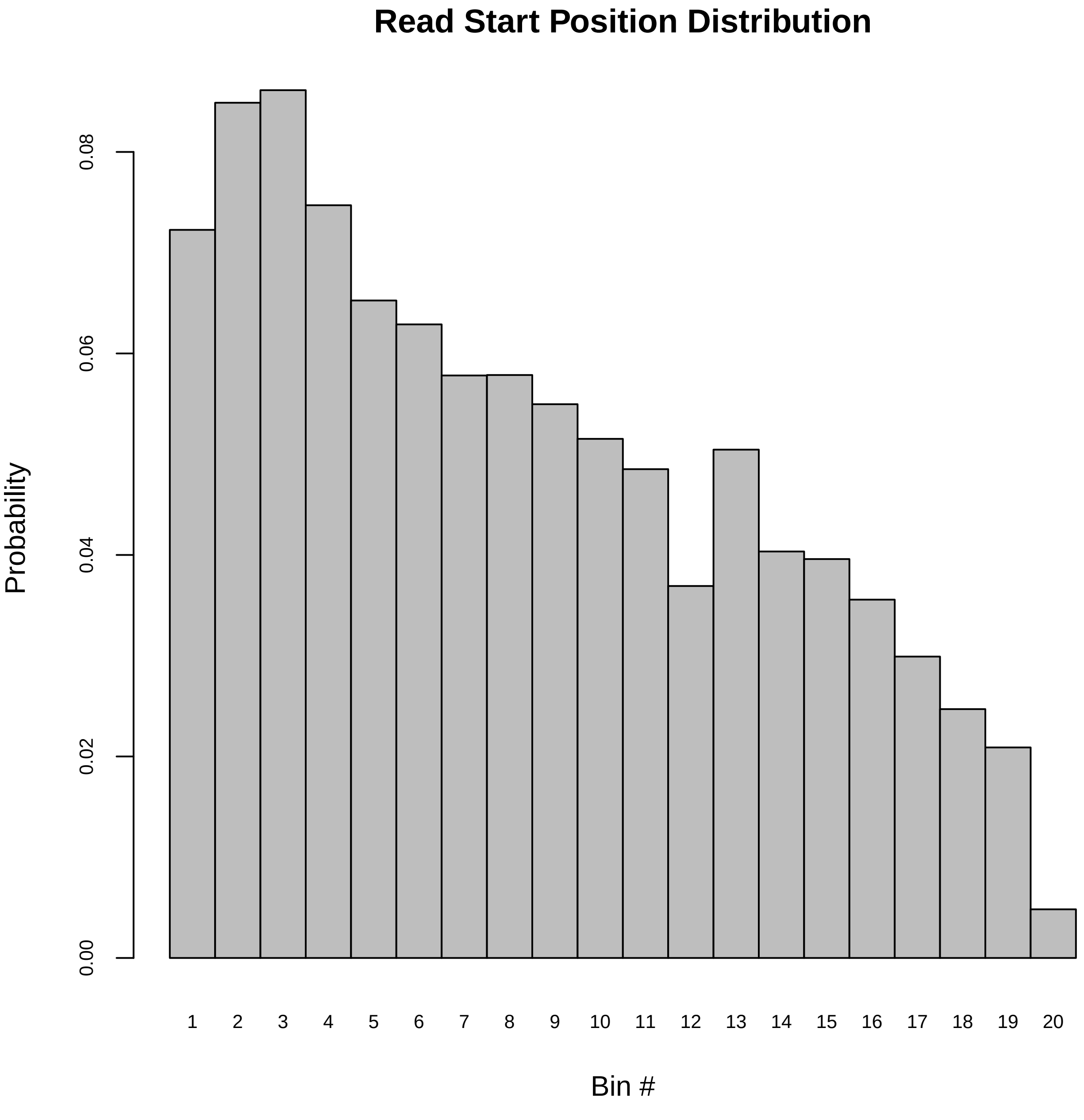
Read Start Position Distribution
Probability
0.00
0.02
0.04
0.06
0.08
1
2
3
4
5
6
7
8
9
10
11
12
13
14
15
16
17
18
19
20
Bin #

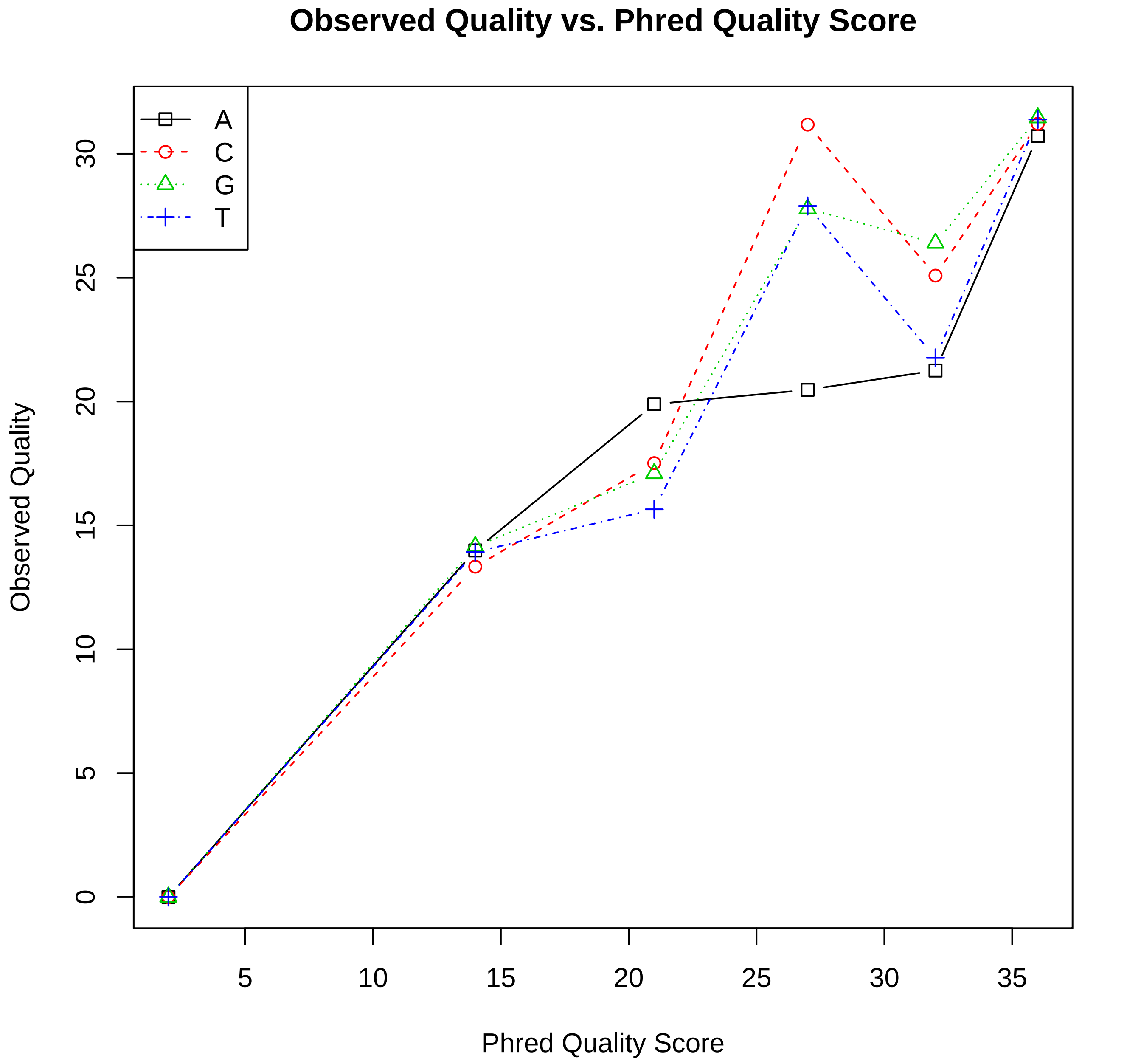
Observed Quality vs. Phred Quality Score
A
C
G
T
Observed Quality
Phred Quality Score
0
5
10
15
20
25
30
35

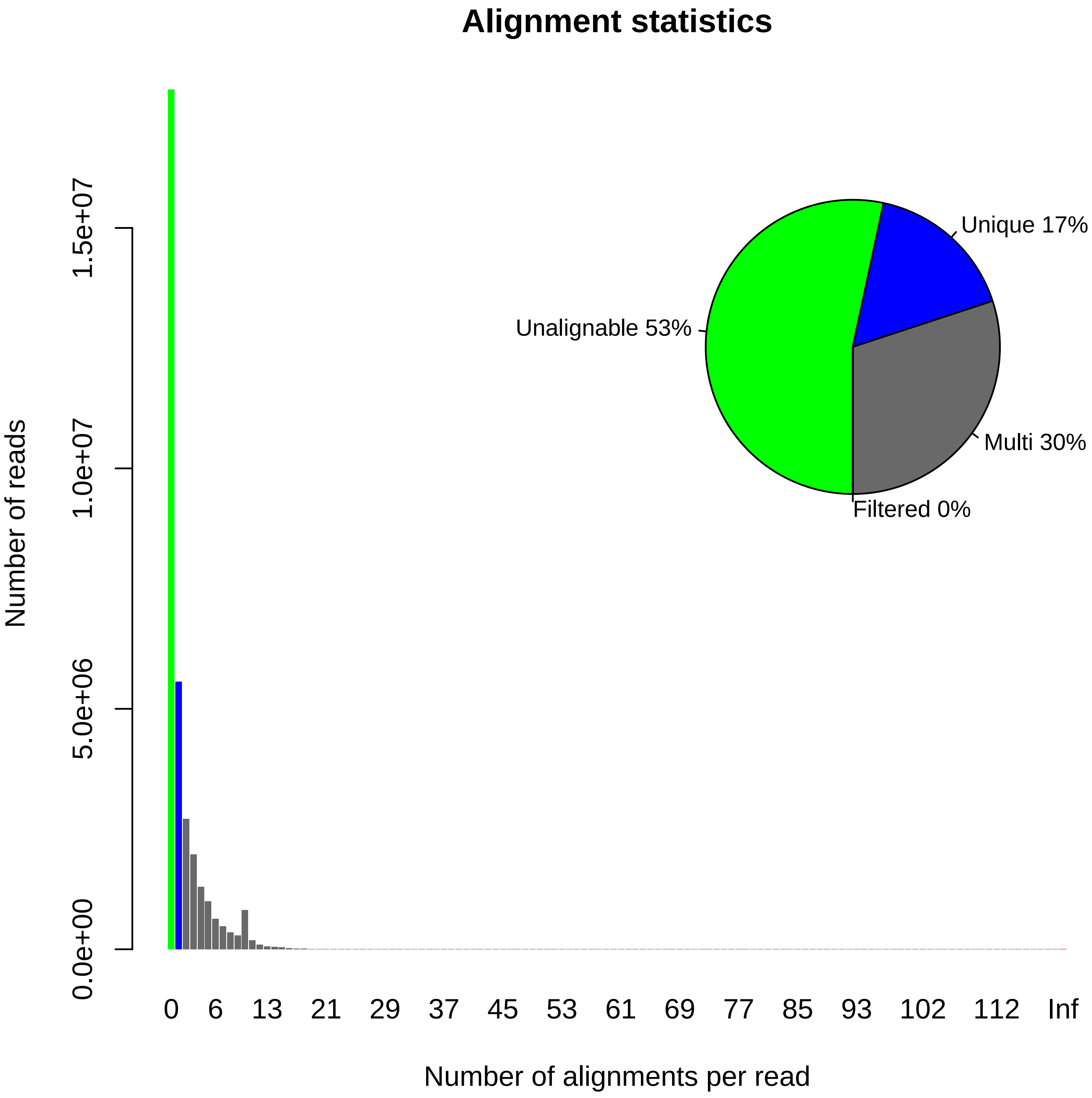
Alignment statistics
Unique 17%
Unalignable 53%
Multi 30%
Filtered 0%
1.5e+07
1.0e+07
5.0e+06
0.0e+00
Number of reads
0
6
13
21
29
37
45
53
61
69
77
85
93
102
112
Inf
Number of alignments per read
