# Supplementary material for: Transcriptomic Changes in Cisplatin-Resistant MCF-7 Cells
Source: Int J Mol Sci. 2024 Mar 29;25(7):3820. doi: 10.3390/ijms25073820 (PMC11011657; doi:10.3390/ijms25073820)
Supplement: Supplementary file 1 [file ijms-25-03820-s001.zip › ijms-2687107-supplementary additions/fastqc_report cpR-MCF-7N2.html]

R\_N2\_1.fastq.gz FastQC Report 

FastQC Report

Fri 13 Jul 2018  
R\_N2\_1.fastq.gz

## Summary

- Basic Statistics
- Per base sequence quality
- Per tile sequence quality
- Per sequence quality scores
- Per base sequence content
- Per sequence GC content
- Per base N content
- Sequence Length Distribution
- Sequence Duplication Levels
- Overrepresented sequences
- Adapter Content

## Basic Statistics

| Measure | Value |
| --- | --- |
| Filename | R\_N2\_1.fastq.gz |
| File type | Conventional base calls |
| Encoding | Sanger / Illumina 1.9 |
| Total Sequences | 35575606 |
| Sequences flagged as poor quality | 0 |
| Sequence length | 35-76 |
| %GC | 45 |

## Per base sequence quality

## Per tile sequence quality

## Per sequence quality scores

## Per base sequence content

## Per sequence GC content

## Per base N content

## Sequence Length Distribution

## Sequence Duplication Levels

## Overrepresented sequences

| Sequence | Count | Percentage | Possible Source |
| --- | --- | --- | --- |
| CTCGCTAATTTGACTATGGATTCATCAAAATGCAACTGAGGTTTGCTCAG | 224999 | 0.6324530353748578 | No Hit |
| CGCTAATTTGACTATGGATTCATCAAAATGCAACTGAGGTTTGCTCAGTT | 204935 | 0.5760548393750482 | No Hit |
| CCCCACTACCACAAATTATGCAGTCGAGTTTCCCACATTTGGGGAAATCGCAGGGGTCAGCACATCCGGAGTGCA | 96050 | 0.26998837349390475 | No Hit |
| CTGATTAGTATTTAGCCTTACCGGGTGGTCCCGGCAGATTCAGACAGGGT | 93337 | 0.26236236144508684 | No Hit |
| GTCTGATTAGTATTTAGCCTTACCGGGTGGTCCCGGCAGATTCAGACAGG | 89632 | 0.2519479218428493 | No Hit |
| GGGCTCTTTCGCTTTCGCTCGCCACTACTGACGAAATCATTATTTATTTT | 84492 | 0.23749981939871945 | No Hit |
| CCGGCATTCTCACTTTTAATCTCTCCACCAGTCCTCACGGTCTGACTTCA | 81236 | 0.22834748057418897 | No Hit |
| CCCCTCCTTAGGCAACCTGGTGGTCCCCCGCTCCCGGGAGGTCACCATAT | 77355 | 0.2174383199544092 | No Hit |
| CCACAATCCAGTAAGTGGTAGAACTATCCTTTTTCGTCACTCCATCATTC | 74643 | 0.20981511882046366 | No Hit |
| CCCTCCTTAGGCAACCTGGTGGTCCCCCGCTCCCGGGAGGTCACCATATT | 74297 | 0.20884254227461369 | No Hit |
| CCCACTACCACAAATTATGCAGTCGAGTTTCCCACATTTGGGGAAATCGC | 73988 | 0.20797396957904246 | No Hit |
| CCTCACGGTACTAGTTCACTATCGGTGTCTGATTAGTATTTAGCCTTACC | 70973 | 0.19949906123876004 | No Hit |
| CTCCATCATTCTTTTACCAAGTACAGGAATATTAACCTGTTGTCCATCGA | 67903 | 0.19086955258049576 | No Hit |
| CTCACTTAACACAATTTTGGGACCTTAGCTGACGATCTGGGTTGTTTCCC | 67096 | 0.1886011442784699 | No Hit |
| CCTTAGGCAACCTGGTGGTCCCCCGCTCCCGGGAGGTCACCATATTGATG | 57664 | 0.16208859520200442 | No Hit |
| CCCCATTCGGAAATCTCCGTATCATAGTTTATTTCCAACTCCACGAAGCT | 57637 | 0.16201270050044966 | No Hit |
| CTCAATGTAAGATGTCCTACAACCCTTTTTTACAGGTTTGGGCTCTTTCG | 55571 | 0.15620535037407374 | No Hit |
| CCCCATTAAACAATACTATACGCTAGCCCTAAAGCTATTTCGAAGAGAAC | 51575 | 0.14497293454396812 | No Hit |
| CCACAAATTATGCAGTCGAGTTTCCCACATTTGGGGAAATCGCAGGGGTCAGCACATCCGGAGTGCAATGGATA | 50388 | 0.14163637859043074 | No Hit |
| CTCCTTAGGCAACCTGGTGGTCCCCCGCTCCCGGGAGGTCACCATATTGA | 50301 | 0.14139182899653205 | No Hit |
| CCGTTACATTATTGGCGCAAGATCTCTTGACTAGTGAGCAATTACGCACT | 48646 | 0.13673976488271206 | No Hit |
| CTCGGTACAGGTTGATAAAAAATTAACACTAGAAGCTTTTCTTGGAAACA | 48257 | 0.1356463189973489 | No Hit |
| CCCATTCGGAAATCTCCGTATCATAGTTTATTTCCAACTCCACGAAGCTT | 46507 | 0.130727217970651 | No Hit |
| CCCTGACTAACCCTGGGTGGACGAACCTTGCCCAGGAAACTTTTCCCAAT | 46073 | 0.12950728091602992 | No Hit |
| CTCCGTTTCCGACCTGGGCCGGTTCACCCCTCCTTAGGCAACCTGGTGGT | 42898 | 0.12058262619616374 | No Hit |
| GGGACCTTAGCTGACGATCTGGGTTGTTTCCCTCGCGAGCGTGGACGTTA | 42721 | 0.12008509426374915 | No Hit |
| CTTCTATGTTGAAGCTTTCCAACTTCTTCTACTATCATAAAATTTTGTAA | 42167 | 0.11852784742444021 | No Hit |
| CCCATTTTTAAGTGAAGCTGTGAAGCTCCTTTCTATTACTCATCATGCGATAAATAACTATATCCGGTATTAGCT | 41535 | 0.11675134922508416 | No Hit |
| CGGCATTCTCACTTTTAATCTCTCCACCAGTCCTCACGGTCTGACTTCAACGAAATTAAAACGCTCTCCTAACGC | 40484 | 0.11379707769419303 | No Hit |
| CCTCCTTAGGCAACCTGGTGGTCCCCCGCTCCCGGGAGGTCACCATATTGATGCCGAACTTAGTGCGGACACCCG | 40344 | 0.1134035496120572 | No Hit |
| CCTGGTTTCGGGTATATGCCAATATACTAAAGTCGCCCTATTCAGACTCG | 40308 | 0.11330235667665084 | No Hit |
| GGCTCTTTCGCTTTCGCTCGCCACTACTGACGAAATCATTATTTATTTTC | 39800 | 0.11187441192147225 | No Hit |
| CTGGAGTCTTGGAAGCTTGACTACCCTACGTTCTCCTACAAATGGACCTTGAGAGCTTGTTTGGAGGTTCTAGC | 39457 | 0.11091026812023946 | No Hit |
| CTCATGGATAGATCACCTGGTTTCGGGTATATGCCAATATACTAAAGTCG | 36928 | 0.1038014644079429 | No Hit |
| CTGGTTTCGGGTATATGCCAATATACTAAAGTCGCCCTATTCAGACTCGG | 36556 | 0.10275580407541055 | No Hit |
| GTTCGTTCTCGGTACAGGTTGATAAAAAATTAACACTAGAAGCTTTTCTT | 35972 | 0.10111422978992964 | No Hit |

## Adapter Content

Produced by FastQC (version 0.11.7)
