# Supplementary material for: Transcriptomic Changes in Cisplatin-Resistant MCF-7 Cells
Source: Int J Mol Sci. 2024 Mar 29;25(7):3820. doi: 10.3390/ijms25073820 (PMC11011657; doi:10.3390/ijms25073820)
Supplement: Supplementary file 1 [file ijms-25-03820-s001.zip › ijms-2687107-supplementary additions/fastqc_report cpR-MCF-7N3.html]

R\_N3\_1.fastq.gz FastQC Report 

FastQC Report

Fri 13 Jul 2018  
R\_N3\_1.fastq.gz

## Summary

- Basic Statistics
- Per base sequence quality
- Per tile sequence quality
- Per sequence quality scores
- Per base sequence content
- Per sequence GC content
- Per base N content
- Sequence Length Distribution
- Sequence Duplication Levels
- Overrepresented sequences
- Adapter Content

## Basic Statistics

| Measure | Value |
| --- | --- |
| Filename | R\_N3\_1.fastq.gz |
| File type | Conventional base calls |
| Encoding | Sanger / Illumina 1.9 |
| Total Sequences | 45508511 |
| Sequences flagged as poor quality | 0 |
| Sequence length | 35-76 |
| %GC | 45 |

## Per base sequence quality

## Per tile sequence quality

## Per sequence quality scores

## Per base sequence content

## Per sequence GC content

## Per base N content

## Sequence Length Distribution

## Sequence Duplication Levels

## Overrepresented sequences

| Sequence | Count | Percentage | Possible Source |
| --- | --- | --- | --- |
| CTCGCTAATTTGACTATGGATTCATCAAAATGCAACTGAGGTTTGCTCAG | 390532 | 0.8581515664179827 | No Hit |
| CGCTAATTTGACTATGGATTCATCAAAATGCAACTGAGGTTTGCTCAGTT | 367816 | 0.8082356287156924 | No Hit |
| CTGATTAGTATTTAGCCTTACCGGGTGGTCCCGGCAGATTCAGACAGGGT | 170892 | 0.3755165709552659 | No Hit |
| CCCCACTACCACAAATTATGCAGTCGAGTTTCCCACATTTGGGGAAATCGCAGGGGTCAGCACATCCGGAGTGCA | 145908 | 0.3206169500909401 | No Hit |
| GTCTGATTAGTATTTAGCCTTACCGGGTGGTCCCGGCAGATTCAGACAGG | 141307 | 0.31050675334114974 | No Hit |
| GGGCTCTTTCGCTTTCGCTCGCCACTACTGACGAAATCATTATTTATTTT | 140475 | 0.3086785238919375 | No Hit |
| CCGGCATTCTCACTTTTAATCTCTCCACCAGTCCTCACGGTCTGACTTCA | 136950 | 0.30093272003559945 | No Hit |
| CTCCATCATTCTTTTACCAAGTACAGGAATATTAACCTGTTGTCCATCGA | 128421 | 0.2821911707900089 | No Hit |
| CCACAATCCAGTAAGTGGTAGAACTATCCTTTTTCGTCACTCCATCATTC | 112622 | 0.2474745877754603 | No Hit |
| CCCACTACCACAAATTATGCAGTCGAGTTTCCCACATTTGGGGAAATCGC | 110085 | 0.24189980639006187 | No Hit |
| CTCACTTAACACAATTTTGGGACCTTAGCTGACGATCTGGGTTGTTTCCC | 90853 | 0.1996395795063477 | No Hit |
| CCTCACGGTACTAGTTCACTATCGGTGTCTGATTAGTATTTAGCCTTACC | 90104 | 0.1979937335238237 | No Hit |
| CCCCTCCTTAGGCAACCTGGTGGTCCCCCGCTCCCGGGAGGTCACCATAT | 89840 | 0.1974136222562852 | No Hit |
| CCCCATTCGGAAATCTCCGTATCATAGTTTATTTCCAACTCCACGAAGCT | 85473 | 0.18781761503908576 | No Hit |
| CCCCATTAAACAATACTATACGCTAGCCCTAAAGCTATTTCGAAGAGAAC | 84607 | 0.18591467429026626 | No Hit |
| CCCTCCTTAGGCAACCTGGTGGTCCCCCGCTCCCGGGAGGTCACCATATT | 84374 | 0.18540268214884023 | No Hit |
| CCGTTACATTATTGGCGCAAGATCTCTTGACTAGTGAGCAATTACGCACT | 84041 | 0.18467095089092236 | No Hit |
| CTCAATGTAAGATGTCCTACAACCCTTTTTTACAGGTTTGGGCTCTTTCG | 79449 | 0.17458053066161625 | No Hit |
| CTCGGTACAGGTTGATAAAAAATTAACACTAGAAGCTTTTCTTGGAAACA | 78654 | 0.172833604685506 | No Hit |
| CCCTGACTAACCCTGGGTGGACGAACCTTGCCCAGGAAACTTTTCCCAAT | 75409 | 0.16570307035534518 | No Hit |
| CTGGAGTCTTGGAAGCTTGACTACCCTACGTTCTCCTACAAATGGACCTTGAGAGCTTGTTTGGAGGTTCTAGC | 73051 | 0.16052162198846717 | No Hit |
| CCCATTCGGAAATCTCCGTATCATAGTTTATTTCCAACTCCACGAAGCTT | 70971 | 0.15595104836543652 | No Hit |
| GGGACCTTAGCTGACGATCTGGGTTGTTTCCCTCGCGAGCGTGGACGTTA | 69079 | 0.15179358428141057 | No Hit |
| CGGCATTCTCACTTTTAATCTCTCCACCAGTCCTCACGGTCTGACTTCAACGAAATTAAAACGCTCTCCTAACGC | 67252 | 0.1477789506231043 | No Hit |
| CCTTAGGCAACCTGGTGGTCCCCCGCTCCCGGGAGGTCACCATATTGATG | 65911 | 0.14483224907094852 | No Hit |
| CCCCCATTAAACAATACTATACGCTAGCCCTAAAGCTATTTCGAAGAGAACCAGCTATCTCCAGGTTCGATTGGA | 61012 | 0.1340672297540124 | No Hit |
| GGCTCTTTCGCTTTCGCTCGCCACTACTGACGAAATCATTATTTATTTTC | 60854 | 0.13372004194995524 | No Hit |
| CTGGTTTCGGGTATATGCCAATATACTAAAGTCGCCCTATTCAGACTCGG | 59803 | 0.13141058383562582 | No Hit |
| CTCCTTAGGCAACCTGGTGGTCCCCCGCTCCCGGGAGGTCACCATATTGA | 59416 | 0.13056019345480233 | No Hit |
| CCTGGTTTCGGGTATATGCCAATATACTAAAGTCGCCCTATTCAGACTCG | 58462 | 0.12846388228347003 | No Hit |
| CTTCTATGTTGAAGCTTTCCAACTTCTTCTACTATCATAAAATTTTGTAA | 57401 | 0.1261324502574914 | No Hit |
| GCCCTGACTAACCCTGGGTGGACGAACCTTGCCCAGGAAACTTTTCCCAATAGGCGTCGAAGATTCTCACTTCGA | 56736 | 0.12467118513282054 | No Hit |
| CCCATTTTTAAGTGAAGCTGTGAAGCTCCTTTCTATTACTCATCATGCGATAAATAACTATATCCGGTATTAGCT | 54900 | 0.12063677495403004 | No Hit |
| CCACAAATTATGCAGTCGAGTTTCCCACATTTGGGGAAATCGCAGGGGTCAGCACATCCGGAGTGCAATGGATA | 52931 | 0.11631011175030534 | No Hit |
| CTCATGGATAGATCACCTGGTTTCGGGTATATGCCAATATACTAAAGTCG | 52326 | 0.1149806900955296 | No Hit |
| GCCCTATTCAGACTCGGTTTCCCTACGGCTCCGCTTTTTTCTGCTTAACC | 52100 | 0.114484079692258 | No Hit |
| CTCCGTTTCCGACCTGGGCCGGTTCACCCCTCCTTAGGCAACCTGGTGGT | 49948 | 0.10975529390535321 | No Hit |
| GTTACATTATTGGCGCAAGATCTCTTGACTAGTGAGCAATTACGCACTCTTTAAAAGGTGGCTGCTTCTAAGCCA | 49884 | 0.10961466087079844 | No Hit |
| GTCTCGCTAATTTGACTATGGATTCATCAAAATGCAACTGAGGTTTGCTC | 49879 | 0.10960367391497385 | No Hit |
| CATTAAACAATACTATACGCTAGCCCTAAAGCTATTTCGAAGAGAACCAG | 49614 | 0.10902136525627042 | No Hit |
| CCGCTTTTTTCTGCTTAACCTTGCATATTAACATAACTCGCCGGCCCATA | 48515 | 0.1066064323660249 | No Hit |
| ATTAAACAATACTATACGCTAGCCCTAAAGCTATTTCGAAGAGAACCAGCTATCTCCAGGTTCGATTGGAATTTC | 47144 | 0.10359380907892153 | No Hit |
| CCCGTTACATTATTGGCGCAAGATCTCTTGACTAGTGAGCAATTACGCAC | 46825 | 0.1028928412973125 | No Hit |
| CCTCCTTAGGCAACCTGGTGGTCCCCCGCTCCCGGGAGGTCACCATATTGATGCCGAACTTAGTGCGGACACCCG | 46613 | 0.10242699437034974 | No Hit |
| CCCCCACTACCACAAATTATGCAGTCGAGTTTCCCACATTTGGGGAAATCGCAGGGGTCAGCACATCCGGAGTGC | 46425 | 0.10201388483134506 | No Hit |
| CAATAGGCGTCGAAGATTCTCACTTCGAATCGTTACTCATGCCGGCATTC | 45926 | 0.10091738664005068 | No Hit |
| GTCTGGAGTCTTGGAAGCTTGACTACCCTACGTTCTCCTACAAATGGACC | 45726 | 0.10047790840706698 | No Hit |

## Adapter Content

Produced by FastQC (version 0.11.7)
