# Supplementary material for: Transcriptomic Changes in Cisplatin-Resistant MCF-7 Cells
Source: Int J Mol Sci. 2024 Mar 29;25(7):3820. doi: 10.3390/ijms25073820 (PMC11011657; doi:10.3390/ijms25073820)
Supplement: Supplementary file 1 [file ijms-25-03820-s001.zip › ijms-2687107-supplementary additions/fastqc_report wt-MCF-7N1.html]

W\_N1\_1.fastq.gz FastQC Report 

FastQC Report

Fri 13 Jul 2018  
W\_N1\_1.fastq.gz

## Summary

- Basic Statistics
- Per base sequence quality
- Per tile sequence quality
- Per sequence quality scores
- Per base sequence content
- Per sequence GC content
- Per base N content
- Sequence Length Distribution
- Sequence Duplication Levels
- Overrepresented sequences
- Adapter Content

## Basic Statistics

| Measure | Value |
| --- | --- |
| Filename | W\_N1\_1.fastq.gz |
| File type | Conventional base calls |
| Encoding | Sanger / Illumina 1.9 |
| Total Sequences | 42431423 |
| Sequences flagged as poor quality | 0 |
| Sequence length | 35-76 |
| %GC | 46 |

## Per base sequence quality

## Per tile sequence quality

## Per sequence quality scores

## Per base sequence content

## Per sequence GC content

## Per base N content

## Sequence Length Distribution

## Sequence Duplication Levels

## Overrepresented sequences

| Sequence | Count | Percentage | Possible Source |
| --- | --- | --- | --- |
| CCCCACTACCACAAATTATGCAGTCGAGTTTCCCACATTTGGGGAAATCGCAGGGGTCAGCACATCCGGAGTGCA | 190980 | 0.4500909620683709 | No Hit |
| CCCCTCCTTAGGCAACCTGGTGGTCCCCCGCTCCCGGGAGGTCACCATAT | 163270 | 0.38478558685151804 | No Hit |
| CCCTCCTTAGGCAACCTGGTGGTCCCCCGCTCCCGGGAGGTCACCATATT | 155102 | 0.3655357021611083 | No Hit |
| CCCACTACCACAAATTATGCAGTCGAGTTTCCCACATTTGGGGAAATCGC | 141448 | 0.33335672008926026 | No Hit |
| CCTTAGGCAACCTGGTGGTCCCCCGCTCCCGGGAGGTCACCATATTGATG | 118308 | 0.27882166478366754 | No Hit |
| CTCCTTAGGCAACCTGGTGGTCCCCCGCTCCCGGGAGGTCACCATATTGA | 104687 | 0.24672045526260103 | No Hit |
| CTCCGTTTCCGACCTGGGCCGGTTCACCCCTCCTTAGGCAACCTGGTGGT | 88742 | 0.20914217277134445 | No Hit |
| CCTCCTTAGGCAACCTGGTGGTCCCCCGCTCCCGGGAGGTCACCATATTGATGCCGAACTTAGTGCGGACACCCG | 83692 | 0.1972406157578076 | No Hit |
| CCACAAATTATGCAGTCGAGTTTCCCACATTTGGGGAAATCGCAGGGGTCAGCACATCCGGAGTGCAATGGATA | 77668 | 0.1830435901242341 | No Hit |
| CTGGAGTCTTGGAAGCTTGACTACCCTACGTTCTCCTACAAATGGACCTTGAGAGCTTGTTTGGAGGTTCTAGC | 76933 | 0.1813113833113728 | No Hit |
| CTCGCTAATTTGACTATGGATTCATCAAAATGCAACTGAGGTTTGCTCAG | 76013 | 0.1791431788653423 | No Hit |
| CGCTAATTTGACTATGGATTCATCAAAATGCAACTGAGGTTTGCTCAGTT | 70803 | 0.16686454281771318 | No Hit |
| CCCCCACTACCACAAATTATGCAGTCGAGTTTCCCACATTTGGGGAAATCGCAGGGGTCAGCACATCCGGAGTGC | 61335 | 0.14455089097530383 | No Hit |
| GTCCCCCACTACCACAAATTATGCAGTCGAGTTTCCCACATTTGGGGAAA | 57161 | 0.13471384167342207 | No Hit |
| GTCTGGAGTCTTGGAAGCTTGACTACCCTACGTTCTCCTACAAATGGACC | 53439 | 0.12594204064285094 | No Hit |
| GCTCCGTTTCCGACCTGGGCCGGTTCACCCCTCCTTAGGCAACCTGGTGGTCCCCCGCTCCCGGGAGGTCACCAT | 45325 | 0.10681942012644734 | No Hit |

## Adapter Content

Produced by FastQC (version 0.11.7)
