# Supplementary material for: Transcriptomic Changes in Cisplatin-Resistant MCF-7 Cells
Source: Int J Mol Sci. 2024 Mar 29;25(7):3820. doi: 10.3390/ijms25073820 (PMC11011657; doi:10.3390/ijms25073820)
Supplement: Supplementary file 1 [file ijms-25-03820-s001.zip › ijms-2687107-supplementary additions/fastqc_report wt-MCF-7N2.html]

W\_N2\_1.fastq.gz FastQC Report 

FastQC Report

Fri 13 Jul 2018  
W\_N2\_1.fastq.gz

## Summary

- Basic Statistics
- Per base sequence quality
- Per tile sequence quality
- Per sequence quality scores
- Per base sequence content
- Per sequence GC content
- Per base N content
- Sequence Length Distribution
- Sequence Duplication Levels
- Overrepresented sequences
- Adapter Content

## Basic Statistics

| Measure | Value |
| --- | --- |
| Filename | W\_N2\_1.fastq.gz |
| File type | Conventional base calls |
| Encoding | Sanger / Illumina 1.9 |
| Total Sequences | 42841067 |
| Sequences flagged as poor quality | 0 |
| Sequence length | 35-76 |
| %GC | 45 |

## Per base sequence quality

## Per tile sequence quality

## Per sequence quality scores

## Per base sequence content

## Per sequence GC content

## Per base N content

## Sequence Length Distribution

## Sequence Duplication Levels

## Overrepresented sequences

| Sequence | Count | Percentage | Possible Source |
| --- | --- | --- | --- |
| CTCGCTAATTTGACTATGGATTCATCAAAATGCAACTGAGGTTTGCTCAG | 155966 | 0.3640572257455679 | No Hit |
| CCCCACTACCACAAATTATGCAGTCGAGTTTCCCACATTTGGGGAAATCGCAGGGGTCAGCACATCCGGAGTGCA | 149095 | 0.3480188763739241 | No Hit |
| CGCTAATTTGACTATGGATTCATCAAAATGCAACTGAGGTTTGCTCAGTT | 143825 | 0.3357175954557808 | No Hit |
| CCCACTACCACAAATTATGCAGTCGAGTTTCCCACATTTGGGGAAATCGC | 102895 | 0.24017842506116852 | No Hit |
| CCCCTCCTTAGGCAACCTGGTGGTCCCCCGCTCCCGGGAGGTCACCATAT | 100682 | 0.23501282075910948 | No Hit |
| CCCTCCTTAGGCAACCTGGTGGTCCCCCGCTCCCGGGAGGTCACCATATT | 94408 | 0.22036799410248115 | No Hit |
| CCACAAATTATGCAGTCGAGTTTCCCACATTTGGGGAAATCGCAGGGGTCAGCACATCCGGAGTGCAATGGATA | 94325 | 0.22017425476354266 | No Hit |
| CCGGCATTCTCACTTTTAATCTCTCCACCAGTCCTCACGGTCTGACTTCA | 81300 | 0.18977118380361535 | No Hit |
| CCACAATCCAGTAAGTGGTAGAACTATCCTTTTTCGTCACTCCATCATTC | 75772 | 0.17686767698853065 | No Hit |
| CCTTAGGCAACCTGGTGGTCCCCCGCTCCCGGGAGGTCACCATATTGATG | 75165 | 0.17545081218448644 | No Hit |
| CTCCATCATTCTTTTACCAAGTACAGGAATATTAACCTGTTGTCCATCGA | 71563 | 0.1670429917163361 | No Hit |
| CTGATTAGTATTTAGCCTTACCGGGTGGTCCCGGCAGATTCAGACAGGGT | 68997 | 0.1610534116715627 | No Hit |
| CTCGGTACAGGTTGATAAAAAATTAACACTAGAAGCTTTTCTTGGAAACA | 65884 | 0.1537870193569175 | No Hit |
| CTCCTTAGGCAACCTGGTGGTCCCCCGCTCCCGGGAGGTCACCATATTGA | 64955 | 0.15161853928614802 | No Hit |
| GTCTGATTAGTATTTAGCCTTACCGGGTGGTCCCGGCAGATTCAGACAGG | 63744 | 0.14879181230476823 | No Hit |
| CTCCGTTTCCGACCTGGGCCGGTTCACCCCTCCTTAGGCAACCTGGTGGT | 63568 | 0.1483809915378625 | No Hit |
| GGGCTCTTTCGCTTTCGCTCGCCACTACTGACGAAATCATTATTTATTTT | 62640 | 0.14621484567599588 | No Hit |
| CTCACTTAACACAATTTTGGGACCTTAGCTGACGATCTGGGTTGTTTCCC | 61078 | 0.14256881136970748 | No Hit |
| CCTCACGGTACTAGTTCACTATCGGTGTCTGATTAGTATTTAGCCTTACC | 54736 | 0.12776525850768378 | No Hit |
| CCCCCACTACCACAAATTATGCAGTCGAGTTTCCCACATTTGGGGAAATCGCAGGGGTCAGCACATCCGGAGTGC | 53145 | 0.12405153214321203 | No Hit |
| CCTCCTTAGGCAACCTGGTGGTCCCCCGCTCCCGGGAGGTCACCATATTGATGCCGAACTTAGTGCGGACACCCG | 53142 | 0.12404452951650341 | No Hit |
| CTGGAGTCTTGGAAGCTTGACTACCCTACGTTCTCCTACAAATGGACCTTGAGAGCTTGTTTGGAGGTTCTAGC | 51451 | 0.12009738226174432 | No Hit |
| GTTCGTTCTCGGTACAGGTTGATAAAAAATTAACACTAGAAGCTTTTCTT | 49395 | 0.1152982487574364 | No Hit |
| GTCCCCCACTACCACAAATTATGCAGTCGAGTTTCCCACATTTGGGGAAA | 44937 | 0.10489234546842635 | No Hit |
| CCCCATTAAACAATACTATACGCTAGCCCTAAAGCTATTTCGAAGAGAAC | 44323 | 0.10345914120206204 | No Hit |

## Adapter Content

Produced by FastQC (version 0.11.7)
