# Supplementary material for: Transcriptomic Changes in Cisplatin-Resistant MCF-7 Cells
Source: Int J Mol Sci. 2024 Mar 29;25(7):3820. doi: 10.3390/ijms25073820 (PMC11011657; doi:10.3390/ijms25073820)
Supplement: Supplementary file 1 [file ijms-25-03820-s001.zip › ijms-2687107-supplementary additions/fastqc_report wt-MCF-7N3.html]

W\_N3\_1.fastq.gz FastQC Report 

FastQC Report

Fri 13 Jul 2018  
W\_N3\_1.fastq.gz

## Summary

- Basic Statistics
- Per base sequence quality
- Per tile sequence quality
- Per sequence quality scores
- Per base sequence content
- Per sequence GC content
- Per base N content
- Sequence Length Distribution
- Sequence Duplication Levels
- Overrepresented sequences
- Adapter Content

## Basic Statistics

| Measure | Value |
| --- | --- |
| Filename | W\_N3\_1.fastq.gz |
| File type | Conventional base calls |
| Encoding | Sanger / Illumina 1.9 |
| Total Sequences | 46201065 |
| Sequences flagged as poor quality | 0 |
| Sequence length | 35-76 |
| %GC | 45 |

## Per base sequence quality

## Per tile sequence quality

## Per sequence quality scores

## Per base sequence content

## Per sequence GC content

## Per base N content

## Sequence Length Distribution

## Sequence Duplication Levels

## Overrepresented sequences

| Sequence | Count | Percentage | Possible Source |
| --- | --- | --- | --- |
| CGCTAATTTGACTATGGATTCATCAAAATGCAACTGAGGTTTGCTCAGTT | 259817 | 0.5623614953464817 | No Hit |
| CTCGCTAATTTGACTATGGATTCATCAAAATGCAACTGAGGTTTGCTCAG | 252920 | 0.5474332680426307 | No Hit |
| CCCCACTACCACAAATTATGCAGTCGAGTTTCCCACATTTGGGGAAATCGCAGGGGTCAGCACATCCGGAGTGCA | 150113 | 0.3249124235556042 | No Hit |
| CCCACTACCACAAATTATGCAGTCGAGTTTCCCACATTTGGGGAAATCGC | 124731 | 0.2699742960470717 | No Hit |
| CCGGCATTCTCACTTTTAATCTCTCCACCAGTCCTCACGGTCTGACTTCA | 118069 | 0.25555471502659083 | No Hit |
| CCACAATCCAGTAAGTGGTAGAACTATCCTTTTTCGTCACTCCATCATTC | 117322 | 0.2539378691811542 | No Hit |
| CTGATTAGTATTTAGCCTTACCGGGTGGTCCCGGCAGATTCAGACAGGGT | 115709 | 0.25044660767019117 | No Hit |
| CTCCATCATTCTTTTACCAAGTACAGGAATATTAACCTGTTGTCCATCGA | 115162 | 0.24926265227868666 | No Hit |
| GGGCTCTTTCGCTTTCGCTCGCCACTACTGACGAAATCATTATTTATTTT | 98724 | 0.21368338587000102 | No Hit |
| CTCGGTACAGGTTGATAAAAAATTAACACTAGAAGCTTTTCTTGGAAACA | 92351 | 0.19988933155545224 | No Hit |
| GTCTGATTAGTATTTAGCCTTACCGGGTGGTCCCGGCAGATTCAGACAGG | 91139 | 0.19726601540462327 | No Hit |
| CTCACTTAACACAATTTTGGGACCTTAGCTGACGATCTGGGTTGTTTCCC | 80508 | 0.1742557233258584 | No Hit |
| CTGGAGTCTTGGAAGCTTGACTACCCTACGTTCTCCTACAAATGGACCTTGAGAGCTTGTTTGGAGGTTCTAGC | 76019 | 0.1645394970873507 | No Hit |
| CCACAAATTATGCAGTCGAGTTTCCCACATTTGGGGAAATCGCAGGGGTCAGCACATCCGGAGTGCAATGGATA | 75515 | 0.16344861314344158 | No Hit |
| CCCCTCCTTAGGCAACCTGGTGGTCCCCCGCTCCCGGGAGGTCACCATAT | 75415 | 0.16323216791647552 | No Hit |
| CCTCACGGTACTAGTTCACTATCGGTGTCTGATTAGTATTTAGCCTTACC | 72259 | 0.1564011565534258 | No Hit |
| CCCTCCTTAGGCAACCTGGTGGTCCCCCGCTCCCGGGAGGTCACCATATT | 72046 | 0.15594012821998798 | No Hit |
| CCTTAGGCAACCTGGTGGTCCCCCGCTCCCGGGAGGTCACCATATTGATG | 61700 | 0.1335467050380765 | No Hit |
| CGGCATTCTCACTTTTAATCTCTCCACCAGTCCTCACGGTCTGACTTCAACGAAATTAAAACGCTCTCCTAACGC | 61101 | 0.13225019812854963 | No Hit |
| CCGTTACATTATTGGCGCAAGATCTCTTGACTAGTGAGCAATTACGCACT | 59807 | 0.12944939689160845 | No Hit |
| CCCCATTAAACAATACTATACGCTAGCCCTAAAGCTATTTCGAAGAGAAC | 59308 | 0.12836933520904767 | No Hit |
| CCCTGACTAACCCTGGGTGGACGAACCTTGCCCAGGAAACCTTTCCCAAT | 58143 | 0.12584774831489273 | No Hit |
| GTTCGTTCTCGGTACAGGTTGATAAAAAATTAACACTAGAAGCTTTTCTT | 56346 | 0.12195822758631213 | No Hit |
| GGGACCTTAGCTGACGATCTGGGTTGTTTCCCTCGCGAGCGTGGACGTTA | 54329 | 0.11759252735840614 | No Hit |
| CCCCATTCGGAAATCTCCGTATCATAGTTTATTTCCAACTCCACGAAGCT | 53765 | 0.1163717762783174 | No Hit |
| CTCAATGTAAGATGTCCTACAACCCCTTTTTACAGGTTTGGGCTCTTTCG | 52980 | 0.1146726812466336 | No Hit |
| CTCCTTAGGCAACCTGGTGGTCCCCCGCTCCCGGGAGGTCACCATATTGA | 52302 | 0.11320518260780352 | No Hit |
| CTGGTTTCGGGTATATGCCAATATACTAAAGTCGCCCTATTCAGACTCGG | 50849 | 0.11006023345998625 | No Hit |
| CCCATTCGGAAATCTCCGTATCATAGTTTATTTCCAACTCCACGAAGCTT | 49147 | 0.10637633569702343 | No Hit |
| CCCCCACTACCACAAATTATGCAGTCGAGTTTCCCACATTTGGGGAAATCGCAGGGGTCAGCACATCCGGAGTGC | 46212 | 0.10002366828556875 | No Hit |

## Adapter Content

Produced by FastQC (version 0.11.7)
