# Supplementary material for: Transcriptomic Changes in Cisplatin-Resistant MCF-7 Cells
Source: Int J Mol Sci. 2024 Mar 29;25(7):3820. doi: 10.3390/ijms25073820 (PMC11011657; doi:10.3390/ijms25073820)
Supplement: Supplementary file 1 [file ijms-25-03820-s001.zip › ijms-2687107-supplementary additions/fastqc_reportcpR-MCF-7N1 .html]

R\_N1\_1.fastq.gz FastQC Report 

FastQC Report

Fri 13 Jul 2018  
R\_N1\_1.fastq.gz

## Summary

- Basic Statistics
- Per base sequence quality
- Per tile sequence quality
- Per sequence quality scores
- Per base sequence content
- Per sequence GC content
- Per base N content
- Sequence Length Distribution
- Sequence Duplication Levels
- Overrepresented sequences
- Adapter Content

## Basic Statistics

| Measure | Value |
| --- | --- |
| Filename | R\_N1\_1.fastq.gz |
| File type | Conventional base calls |
| Encoding | Sanger / Illumina 1.9 |
| Total Sequences | 54800385 |
| Sequences flagged as poor quality | 0 |
| Sequence length | 35-76 |
| %GC | 44 |

## Per base sequence quality

## Per tile sequence quality

## Per sequence quality scores

## Per base sequence content

## Per sequence GC content

## Per base N content

## Sequence Length Distribution

## Sequence Duplication Levels

## Overrepresented sequences

| Sequence | Count | Percentage | Possible Source |
| --- | --- | --- | --- |
| CGCTAATTTGACTATGGATTCATCAAAATGCAACTGAGGTTTGCTCAGTT | 354199 | 0.6463439992255529 | No Hit |
| CTCGCTAATTTGACTATGGATTCATCAAAATGCAACTGAGGTTTGCTCAG | 353211 | 0.6445410921839326 | No Hit |
| CCCCACTACCACAAATTATGCAGTCGAGTTTCCCACATTTGGGGAAATCGCAGGGGTCAGCACATCCGGAGTGCA | 181683 | 0.331535991946042 | No Hit |
| CTGATTAGTATTTAGCCTTACCGGGTGGTCCCGGCAGATTCAGACAGGGT | 158951 | 0.29005453155119254 | No Hit |
| CCCACTACCACAAATTATGCAGTCGAGTTTCCCACATTTGGGGAAATCGC | 153195 | 0.2795509557095265 | No Hit |
| CCCCTCCTTAGGCAACCTGGTGGTCCCCCGCTCCCGGGAGGTCACCATAT | 149600 | 0.2729907828202302 | No Hit |
| CCCTCCTTAGGCAACCTGGTGGTCCCCCGCTCCCGGGAGGTCACCATATT | 140077 | 0.25561316768121245 | No Hit |
| CCTTAGGCAACCTGGTGGTCCCCCGCTCCCGGGAGGTCACCATATTGATG | 126449 | 0.23074472925692036 | No Hit |
| GTCTGATTAGTATTTAGCCTTACCGGGTGGTCCCGGCAGATTCAGACAGG | 118590 | 0.2164035891353683 | No Hit |
| GGGCTCTTTCGCTTTCGCTCGCCACTACTGACGAAATCATTATTTATTTT | 116511 | 0.2126098201682342 | No Hit |
| CTGGAGTCTTGGAAGCTTGACTACCCTACGTTCTCCTACAAATGGACCTTGAGAGCTTGTTTGGAGGTTCTAGC | 113591 | 0.20728139045008534 | No Hit |
| CTCCTTAGGCAACCTGGTGGTCCCCCGCTCCCGGGAGGTCACCATATTGA | 108017 | 0.19710992906345456 | No Hit |
| CCTCACGGTACTAGTTCACTATCGGTGTCTGATTAGTATTTAGCCTTACC | 104871 | 0.19136909348355854 | No Hit |
| CCACAAATTATGCAGTCGAGTTTCCCACATTTGGGGAAATCGCAGGGGTCAGCACATCCGGAGTGCAATGGATA | 103506 | 0.18887823507079377 | No Hit |
| CCACAATCCAGTAAGTGGTAGAACTATCCTTTTTCGTCACTCCATCATTC | 101070 | 0.18443301082647504 | No Hit |
| CCGGCATTCTCACTTTTAATCTCTCCACCAGTCCTCACGGTCTGACTTCA | 100531 | 0.1834494410942551 | No Hit |
| CTCCATCATTCTTTTACCAAGTACAGGAATATTAACCTGTTGTCCATCGA | 99285 | 0.18117573444055185 | No Hit |
| CTCCGTTTCCGACCTGGGCCGGTTCACCCCTCCTTAGGCAACCTGGTGGT | 94132 | 0.17177251583177744 | No Hit |
| CTCAATGTAAGATGTCCTACAACCCTTTTTTACAGGTTTGGGCTCTTTCG | 88286 | 0.16110470756729173 | No Hit |
| CTCACTTAACACAATTTTGGGACCTTAGCTGACGATCTGGGTTGTTTCCC | 85881 | 0.1567160522686109 | No Hit |
| CCTCCTTAGGCAACCTGGTGGTCCCCCGCTCCCGGGAGGTCACCATATTGATGCCGAACTTAGTGCGGACACCCG | 76872 | 0.14027638674436319 | No Hit |
| CCCCATTCGGAAATCTCCGTATCATAGTTTATTTCCAACTCCACGAAGCT | 76143 | 0.13894610411952396 | No Hit |
| CCCATTCGGAAATCTCCGTATCATAGTTTATTTCCAACTCCACGAAGCTT | 68775 | 0.12550094310468804 | No Hit |
| GTCTGGAGTCTTGGAAGCTTGACTACCCTACGTTCTCCTACAAATGGACC | 66554 | 0.1214480518704385 | No Hit |
| CCCTGACTAACCCTGGGTGGACGAACCTTGCCCAGGAAACTTTTCCCAAT | 64924 | 0.11847362021270472 | No Hit |
| CTCGGTACAGGTTGATAAAAAATTAACACTAGAAGCTTTTCTTGGAAACA | 62960 | 0.11488970378584018 | No Hit |
| CCCCATTAAACAATACTATACGCTAGCCCTAAAGCTATTTCGAAGAGAAC | 59367 | 0.10833318050593987 | No Hit |
| CTTCTATGTTGAAGCTTTCCAACTTCTTCTACTATCATAAAATTTTGTAA | 59276 | 0.10816712327842222 | No Hit |
| CTGGTTTCGGGTATATGCCAATATACTAAAGTCGCCCTATTCAGACTCGG | 58469 | 0.10669450588713929 | No Hit |
| CTCTGGAGTTGGAGTTGATCCACCTTGGTTATTTCCTTGCTCTGGAGTTG | 56145 | 0.10245365976899615 | No Hit |
| GGCTCTTTCGCTTTCGCTCGCCACTACTGACGAAATCATTATTTATTTTC | 55468 | 0.10121826698845274 | No Hit |

## Adapter Content

Produced by FastQC (version 0.11.7)
