# Supplementary material for: Transcriptomic Changes in Cisplatin-Resistant MCF-7 Cells
Source: Int J Mol Sci. 2024 Mar 29;25(7):3820. doi: 10.3390/ijms25073820 (PMC11011657; doi:10.3390/ijms25073820)
Supplement: Supplementary file 1 [file ijms-25-03820-s001.zip › ijms-2687107-supplementary additions/resultados de analisis con datos que faltaban .pptx]

## Slide 1
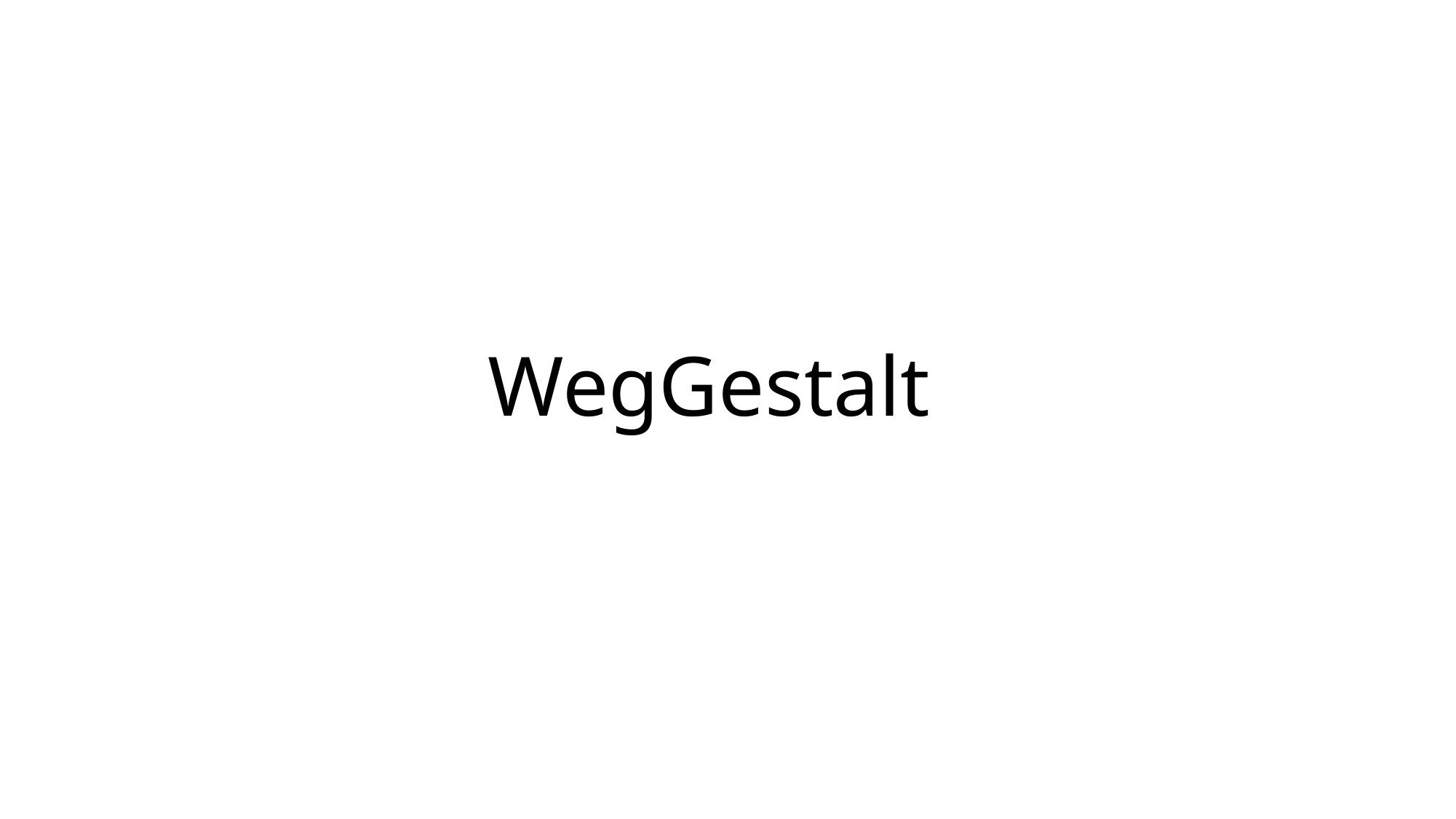

# WegGestalt

## Slide 2
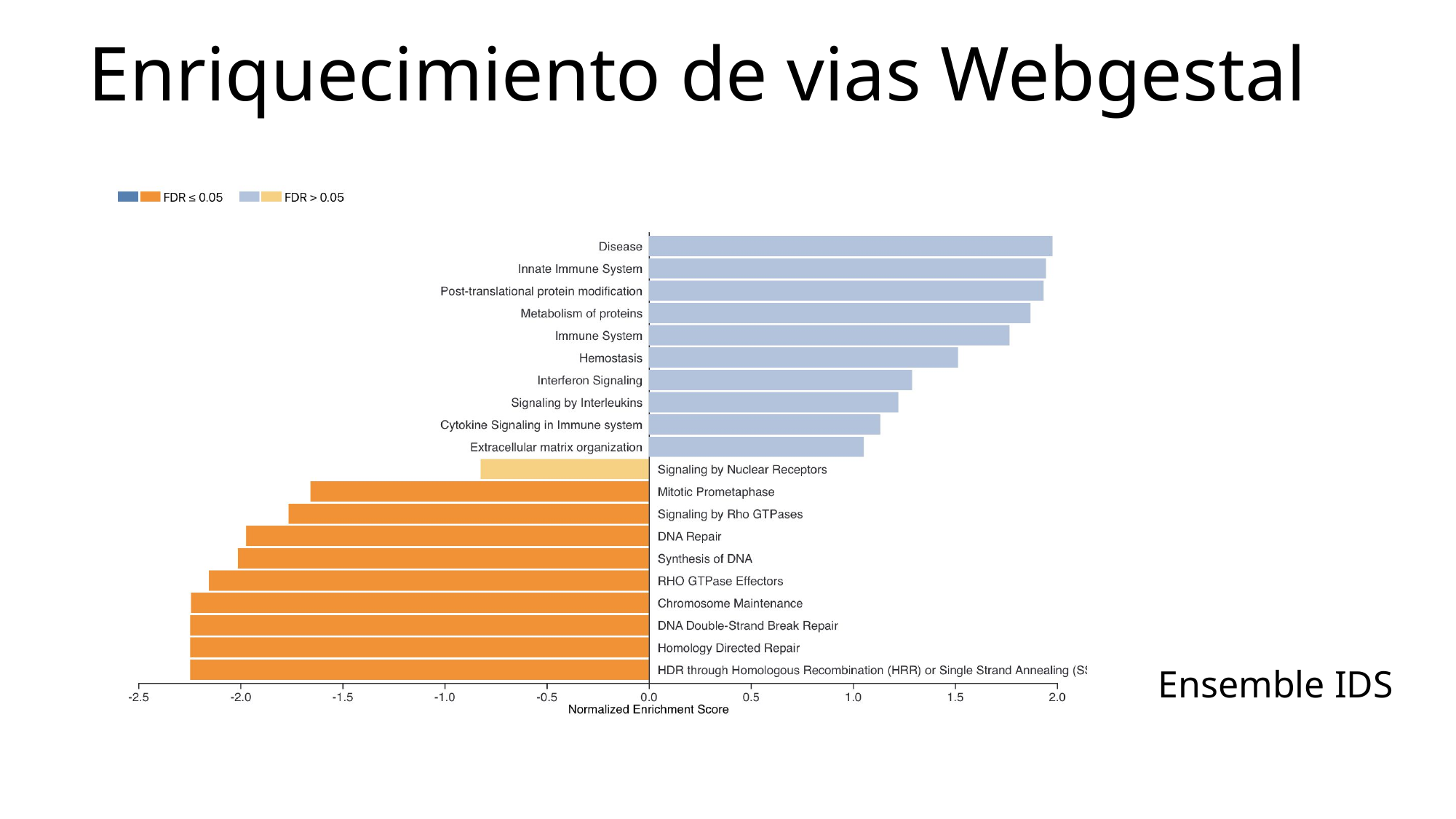

# Enriquecimiento de vias Webgestal
Ensemble IDS

## Slide 3
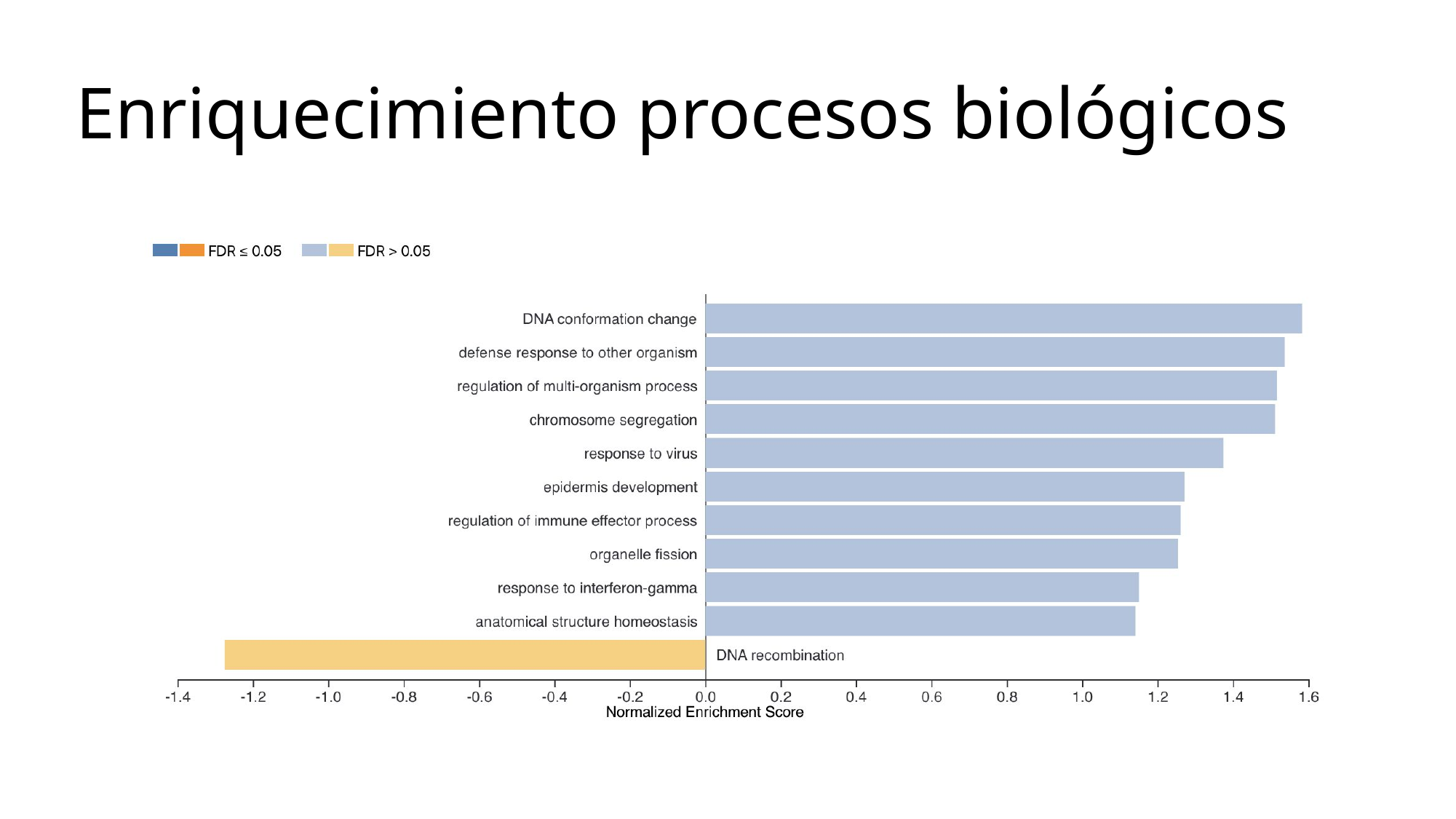

# Enriquecimiento procesos biológicos

## Slide 4
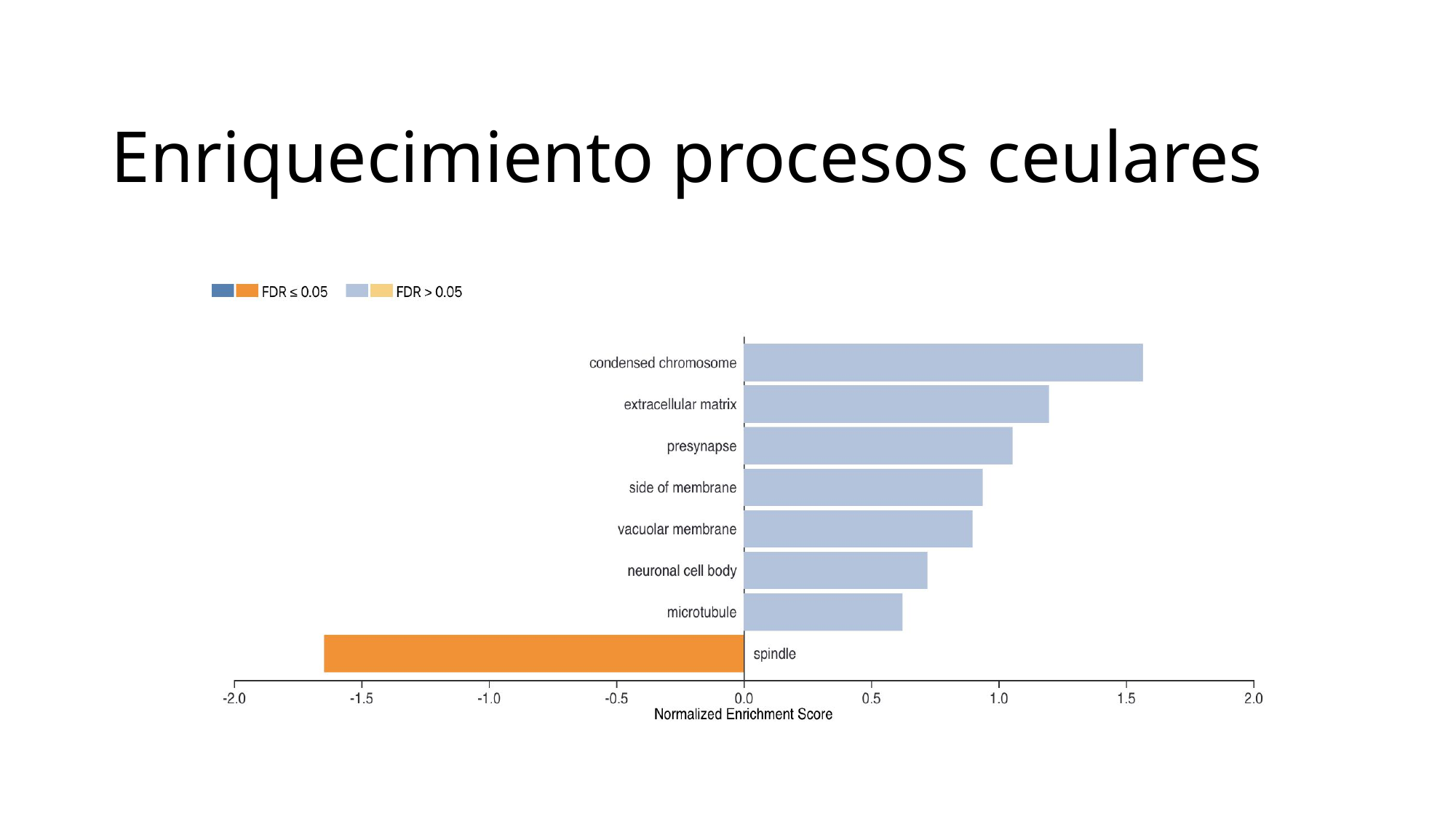

# Enriquecimiento procesos ceulares

## Slide 5
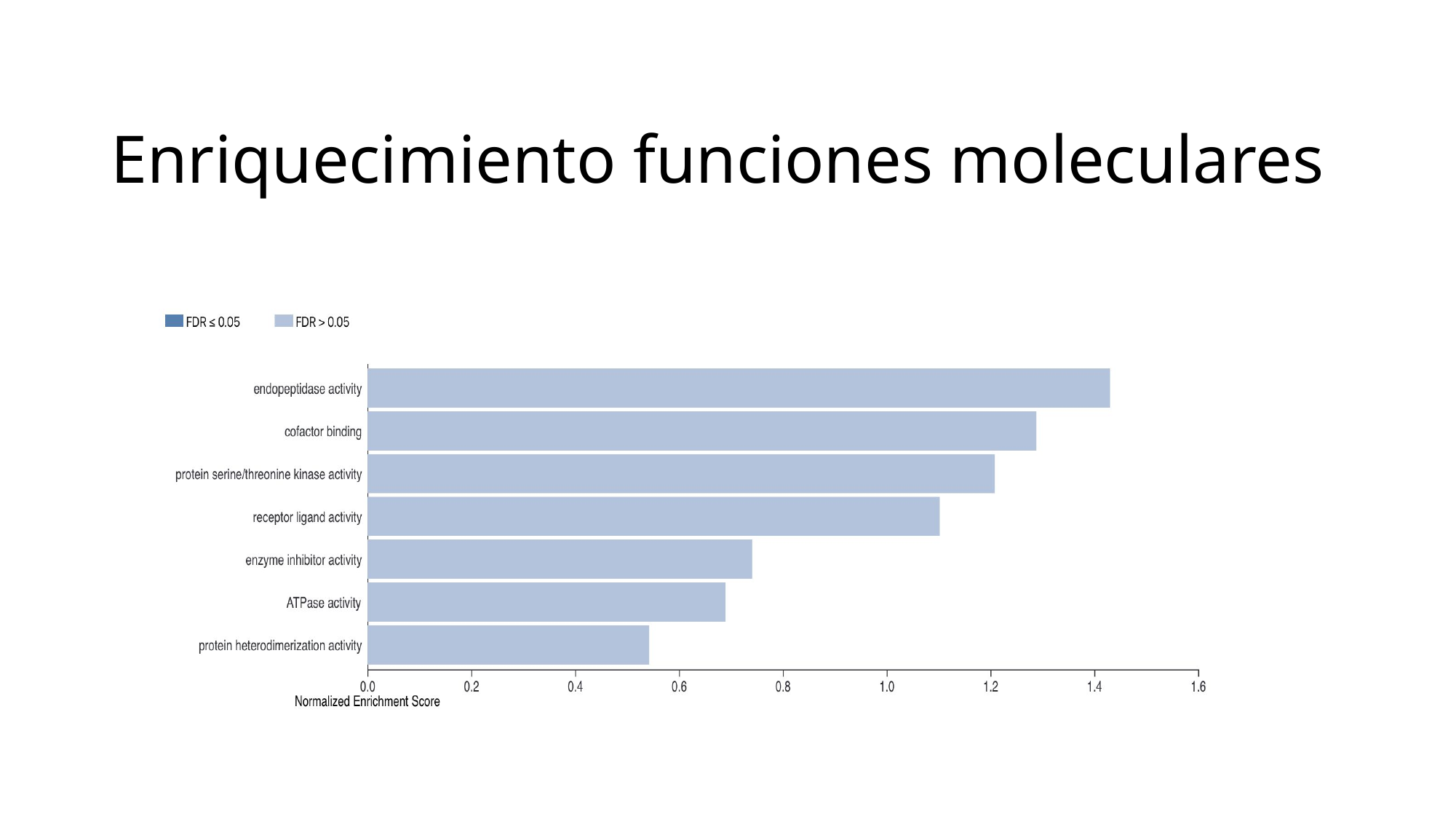

# Enriquecimiento funciones moleculares

## Slide 6
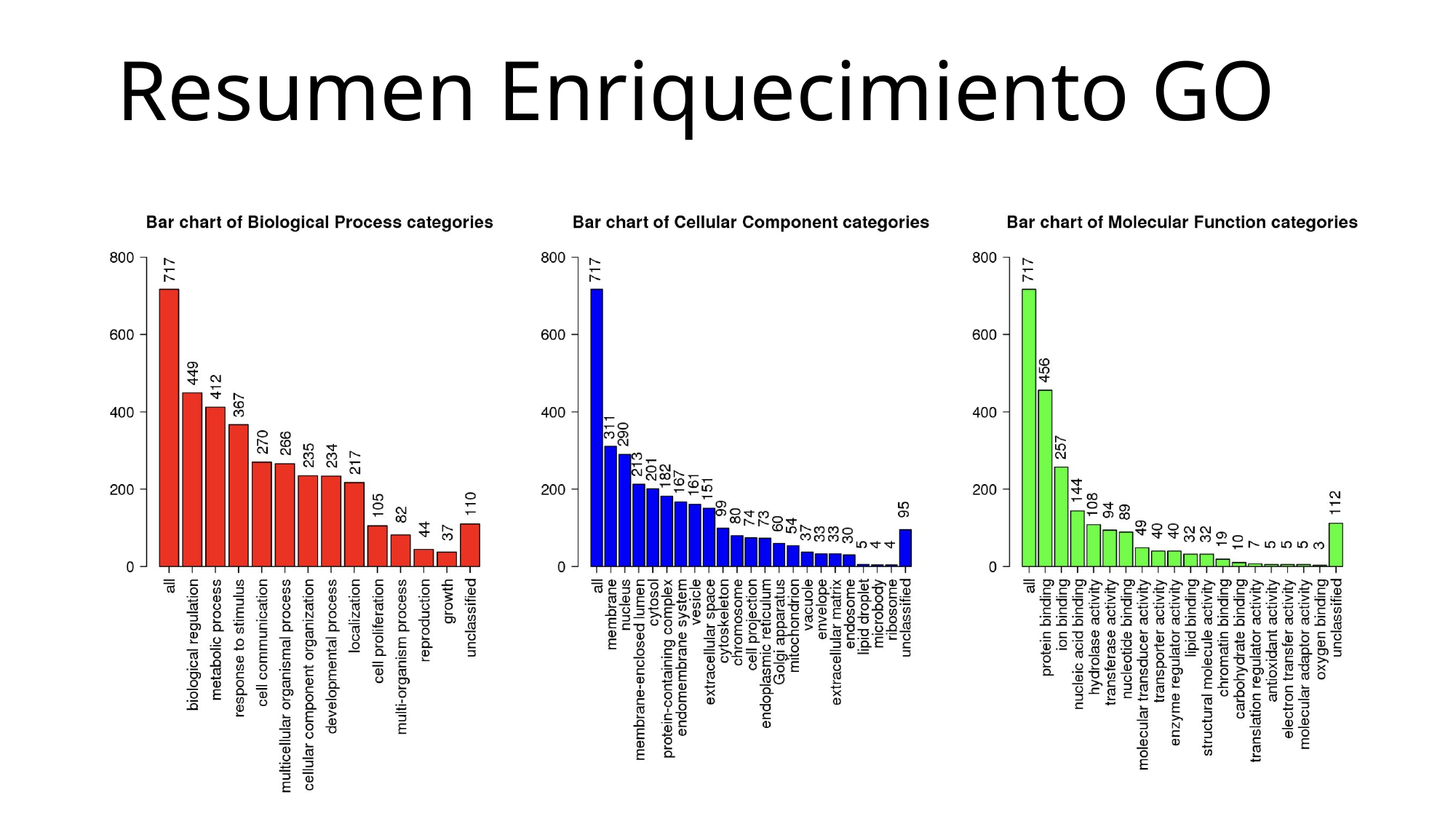

# Resumen Enriquecimiento GO

## Slide 7
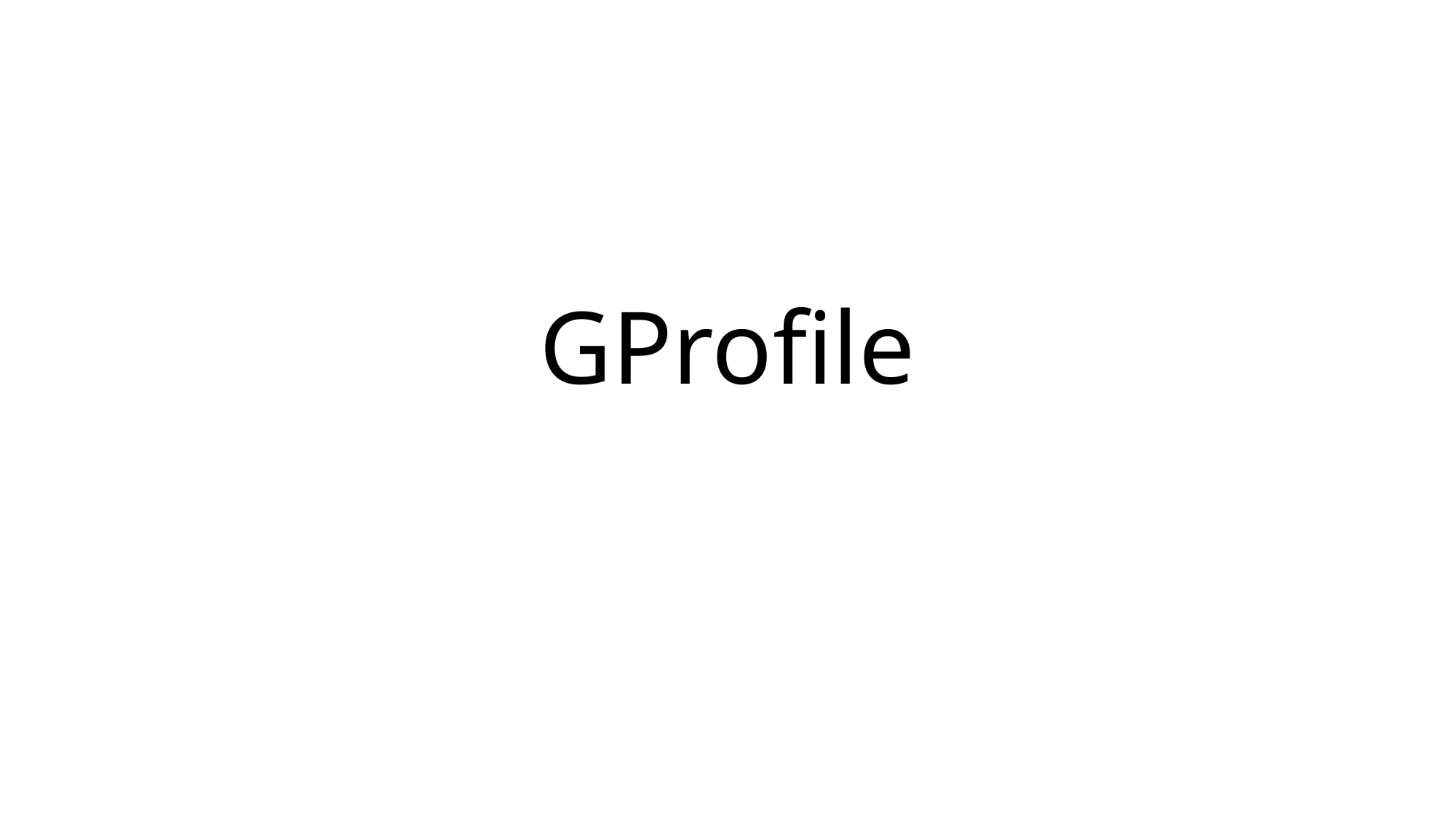

# GProfile

## Slide 8
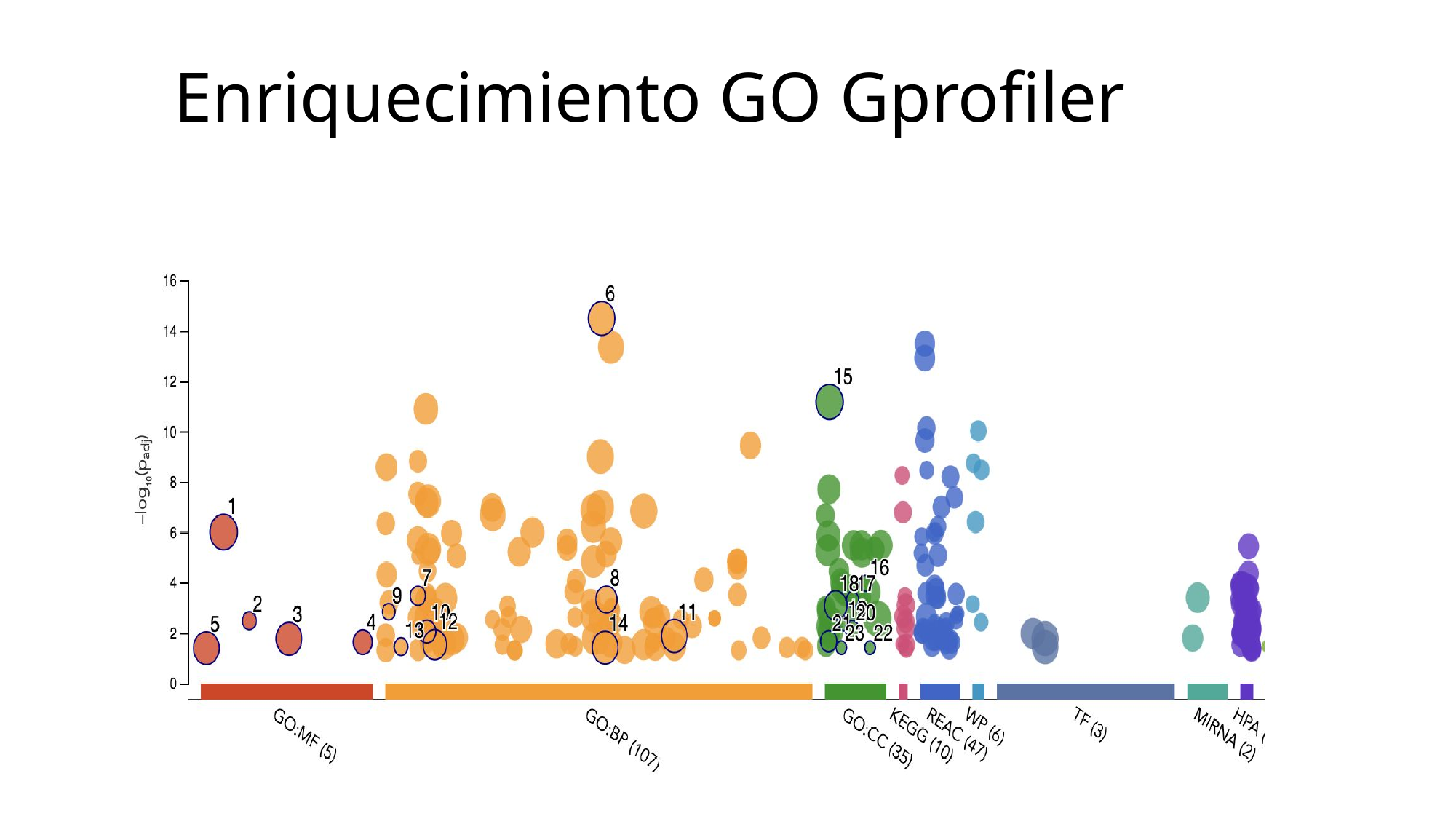

# Enriquecimiento GO Gprofiler

## Slide 9
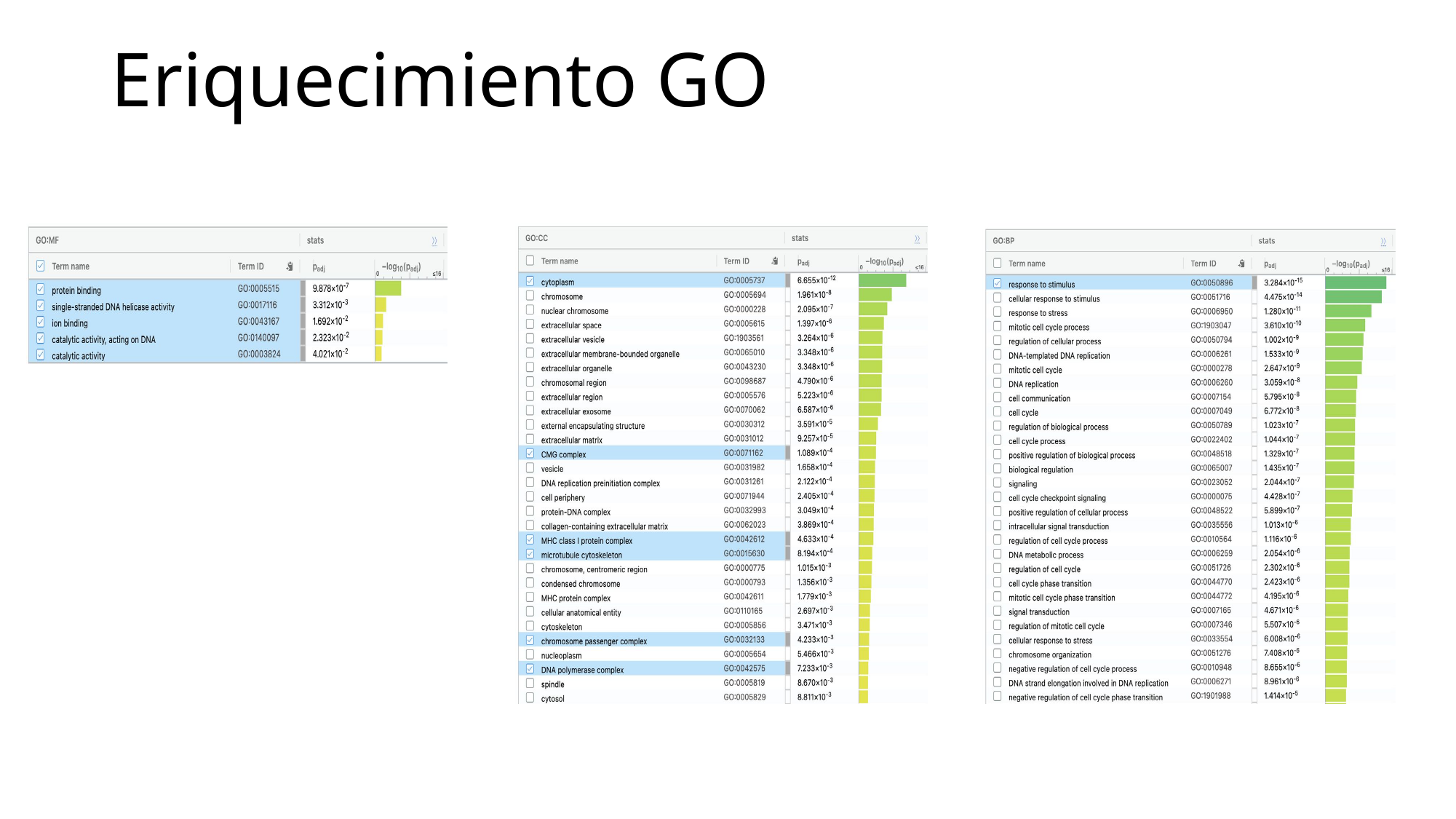

# Eriquecimiento GO

## Slide 10
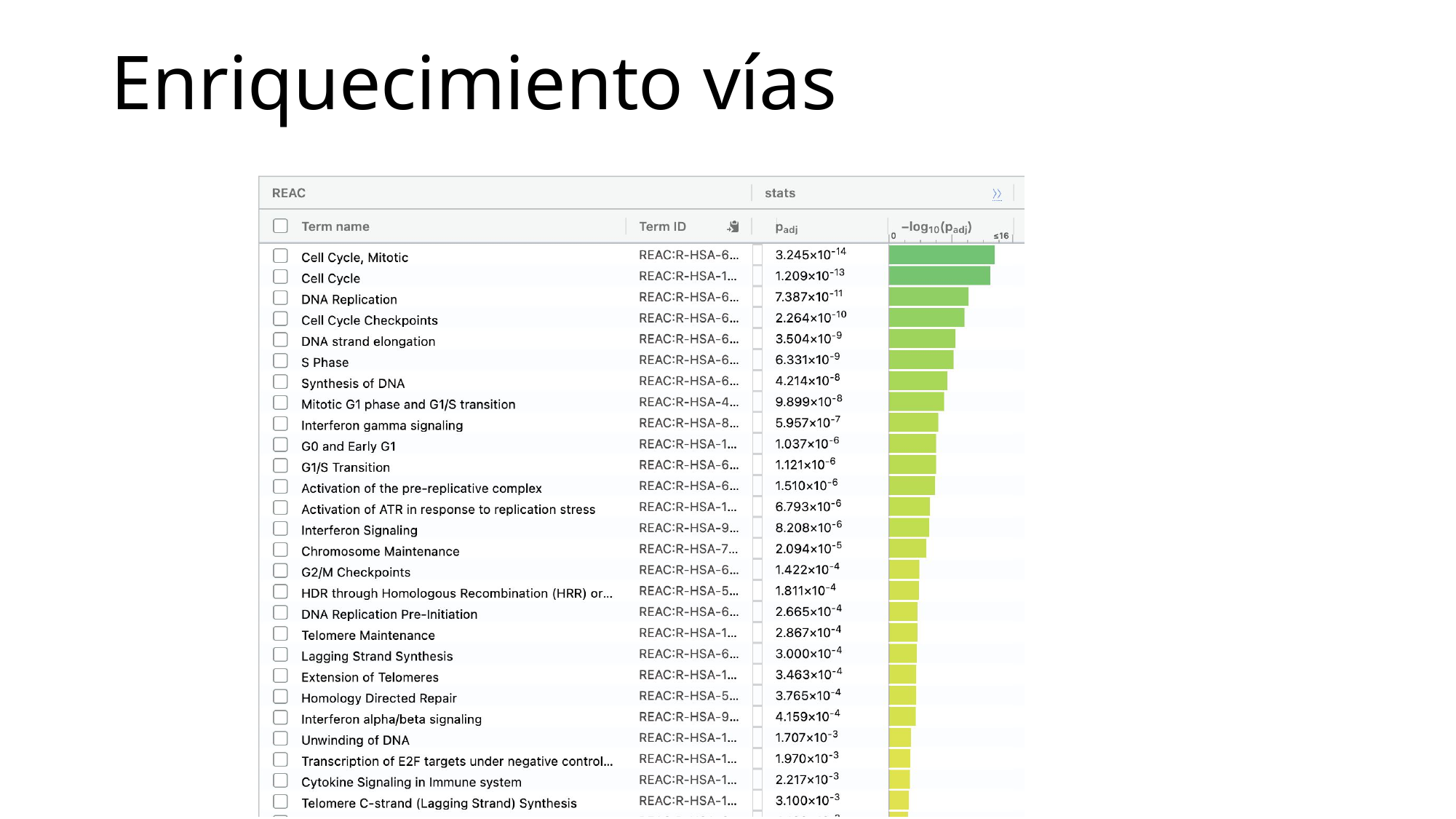

# Enriquecimiento vías

## Slide 11
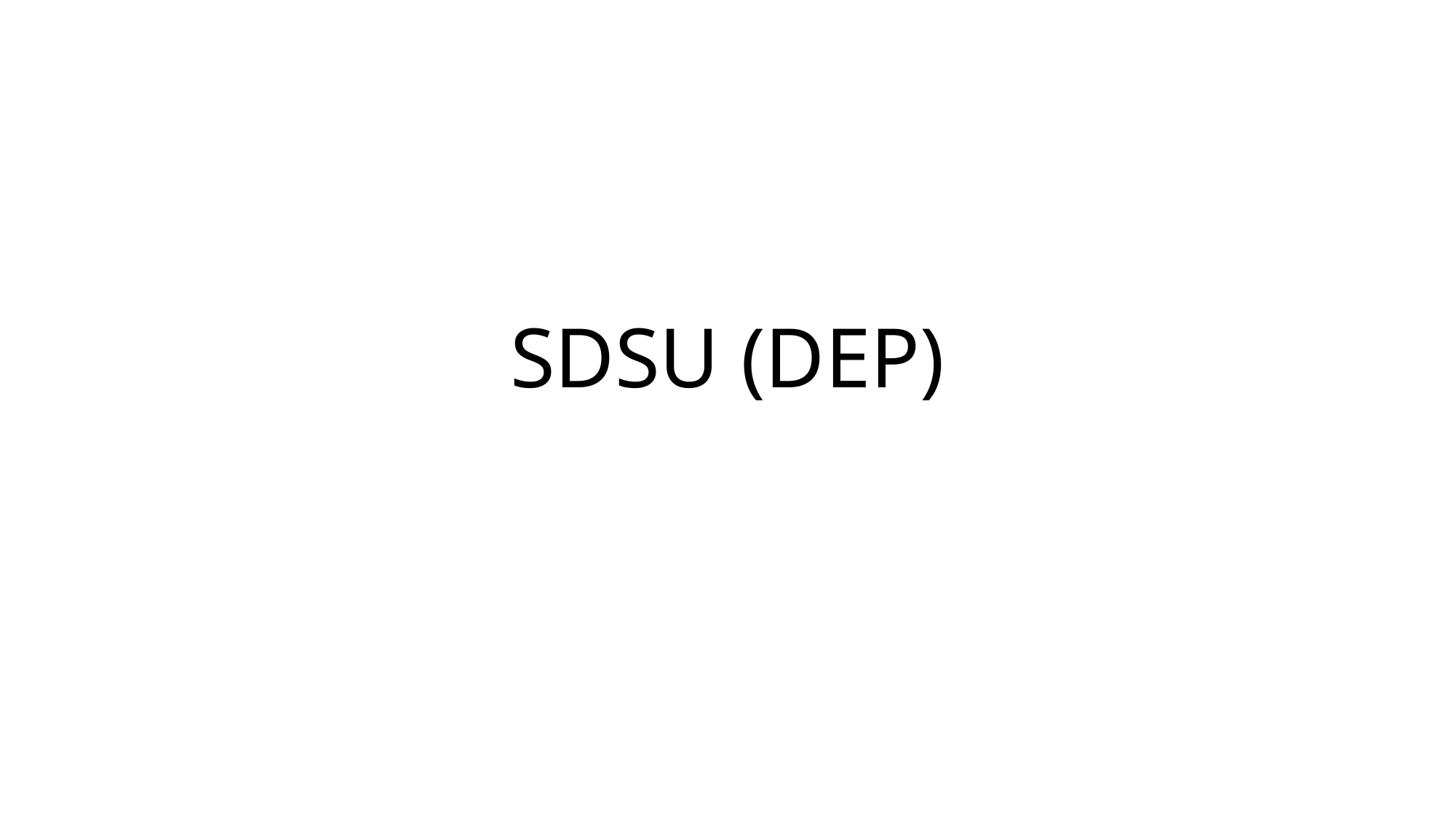

# SDSU (DEP)

## Slide 12
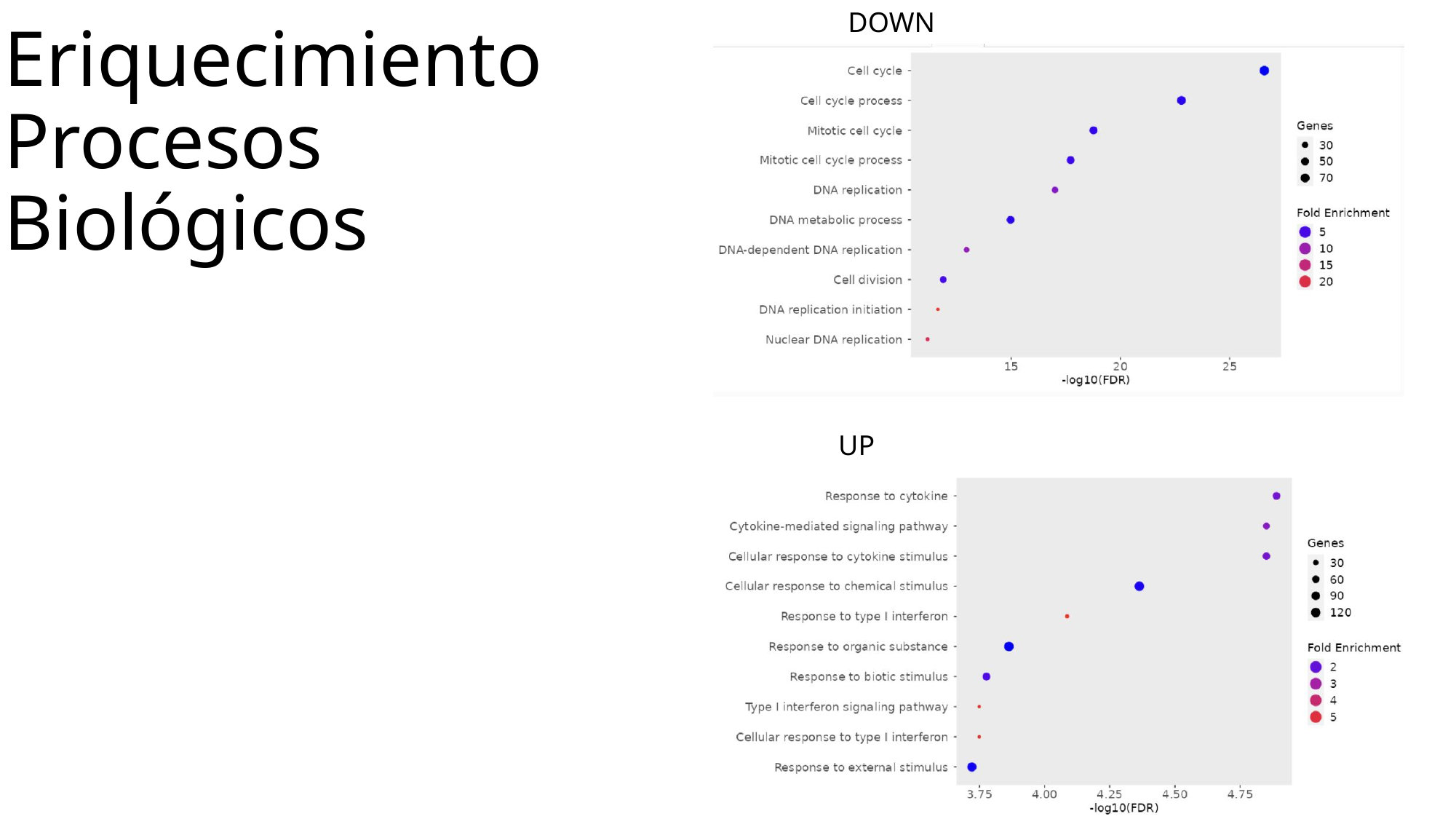

DOWN
# Eriquecimiento Procesos Biológicos
UP

## Slide 13
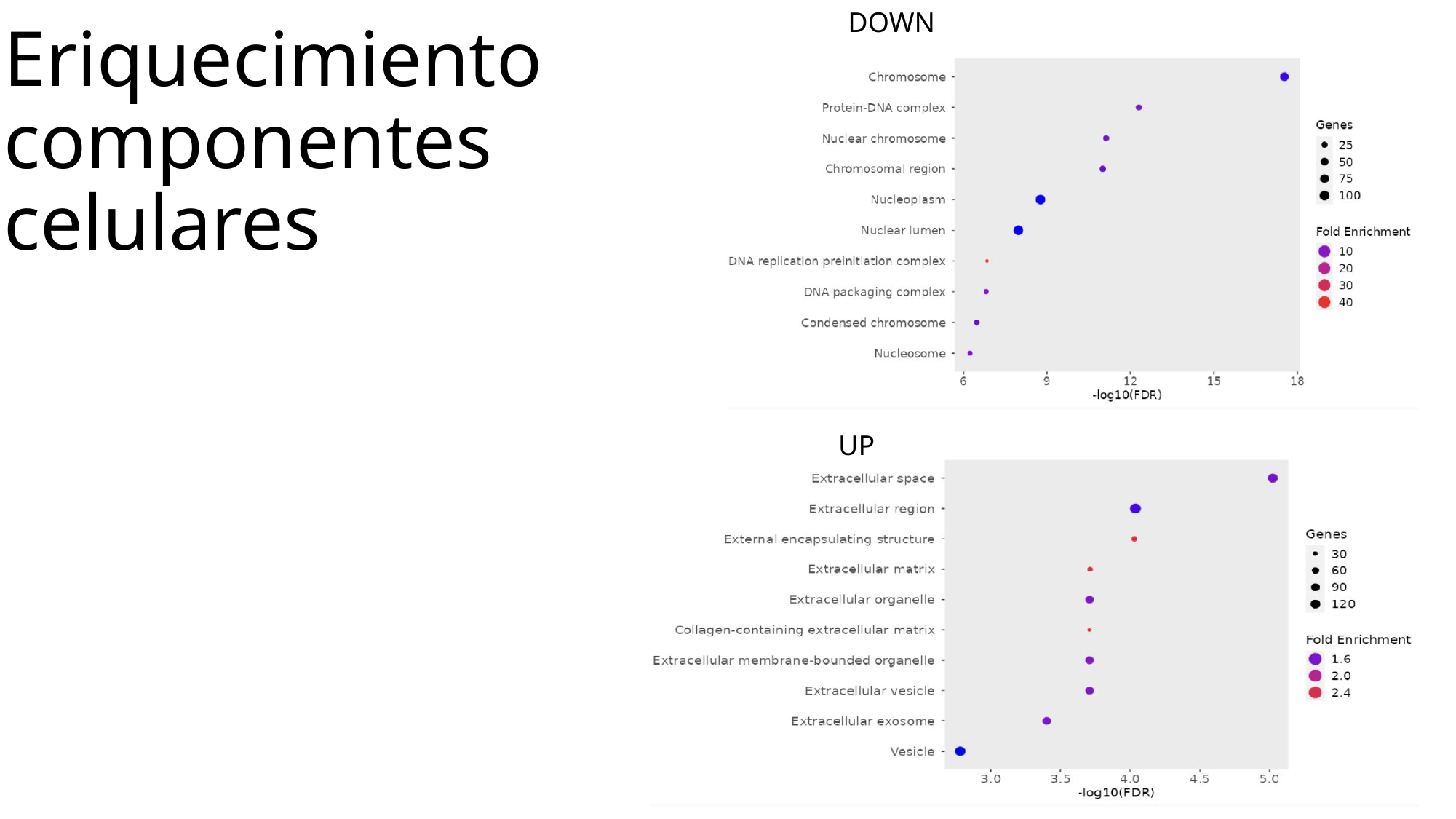

DOWN
# Eriquecimiento componentes celulares
UP

## Slide 14
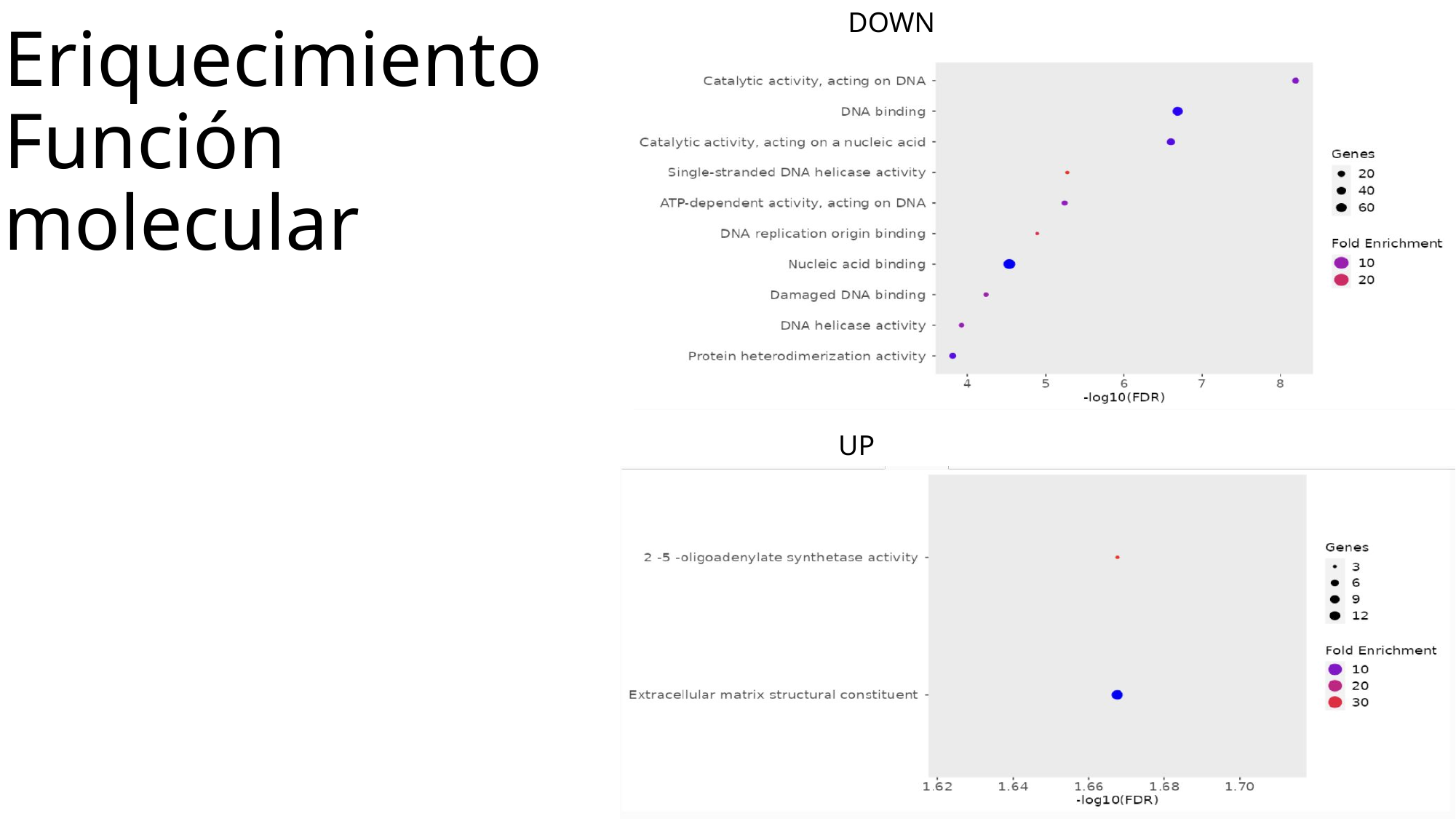

DOWN
# Eriquecimiento Función molecular
UP

## Slide 15
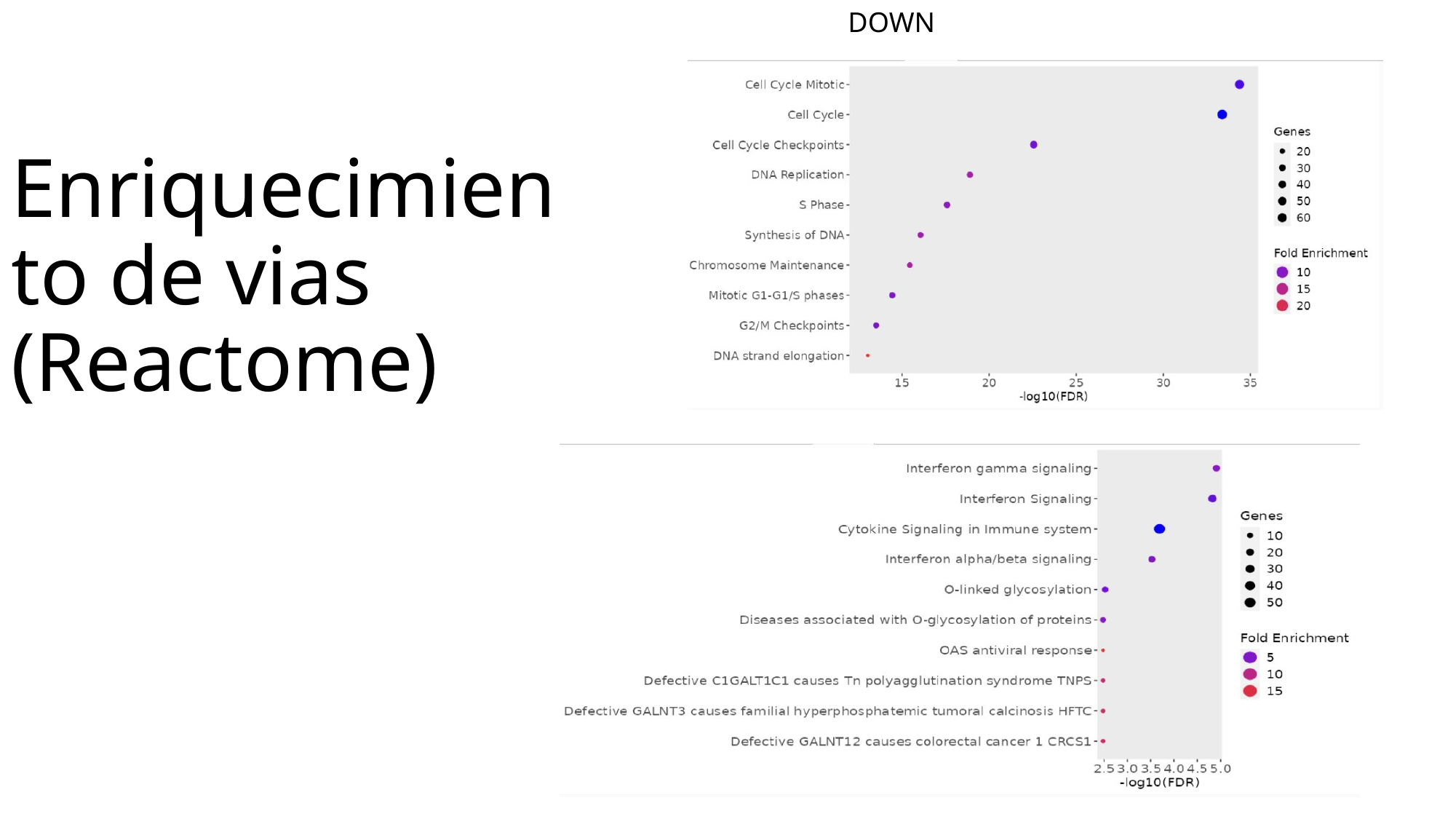

DOWN
# Enriquecimiento de vias (Reactome)

## Slide 16
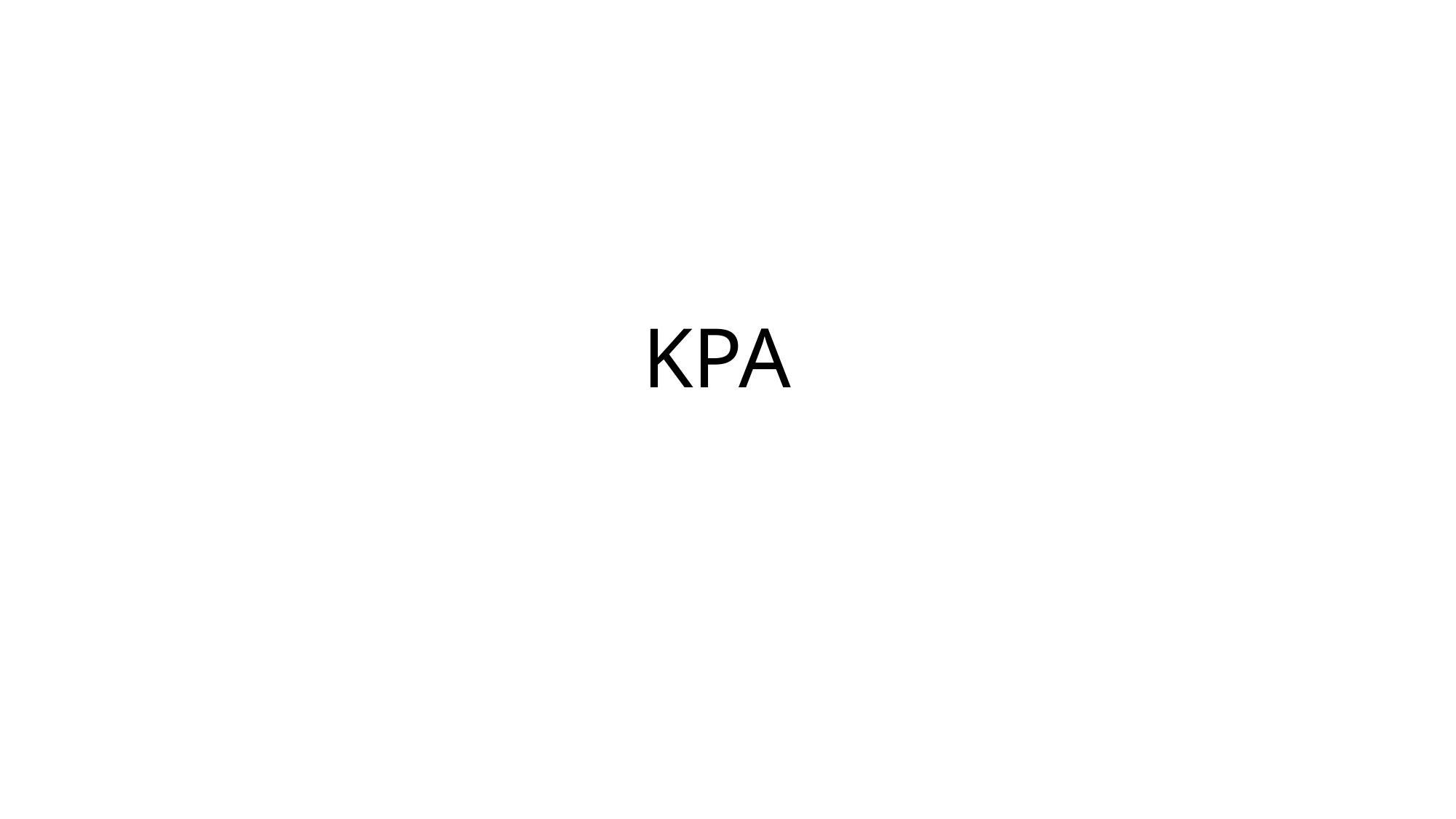

# KPA

## Slide 17
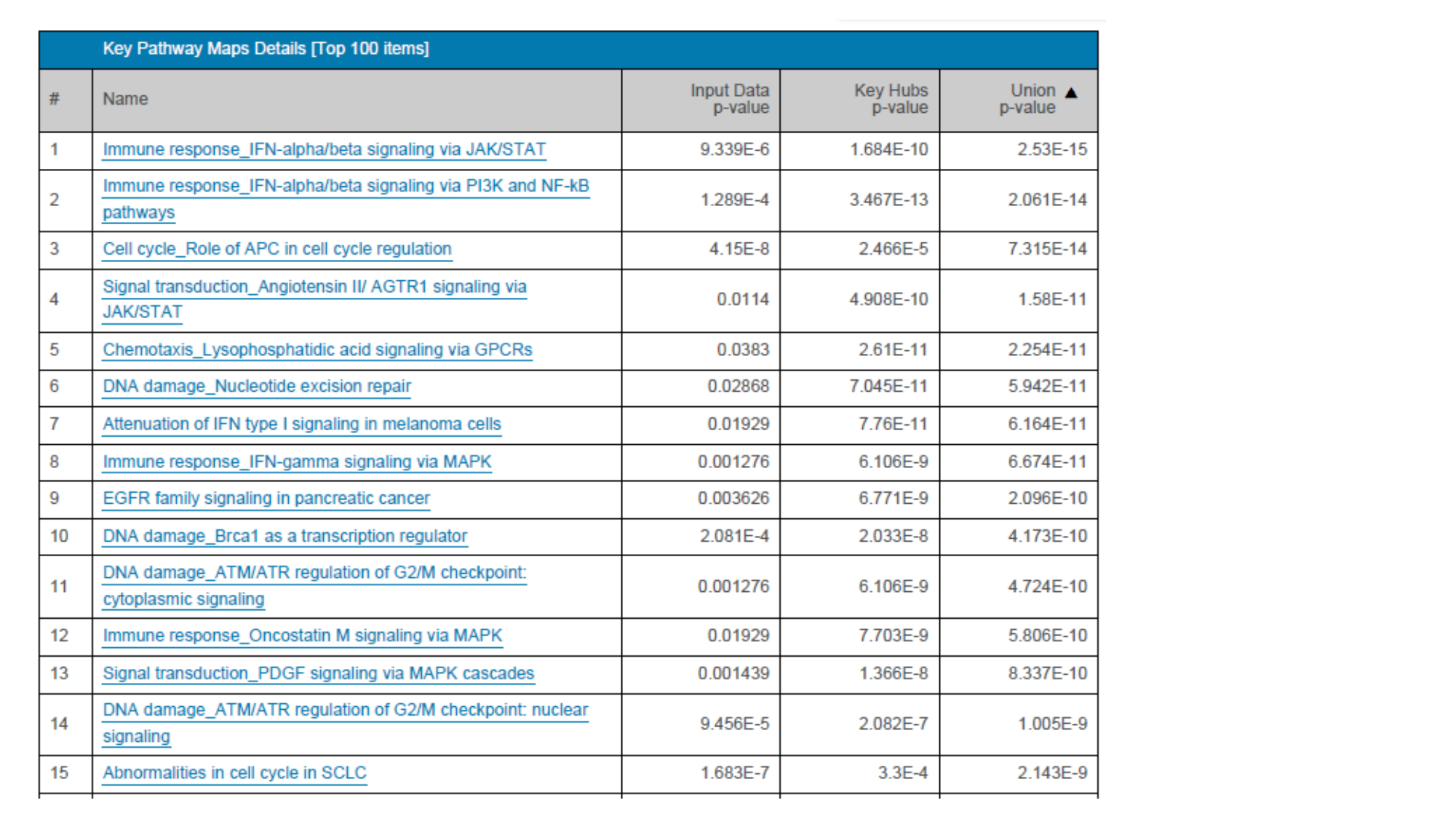

## Slide 18
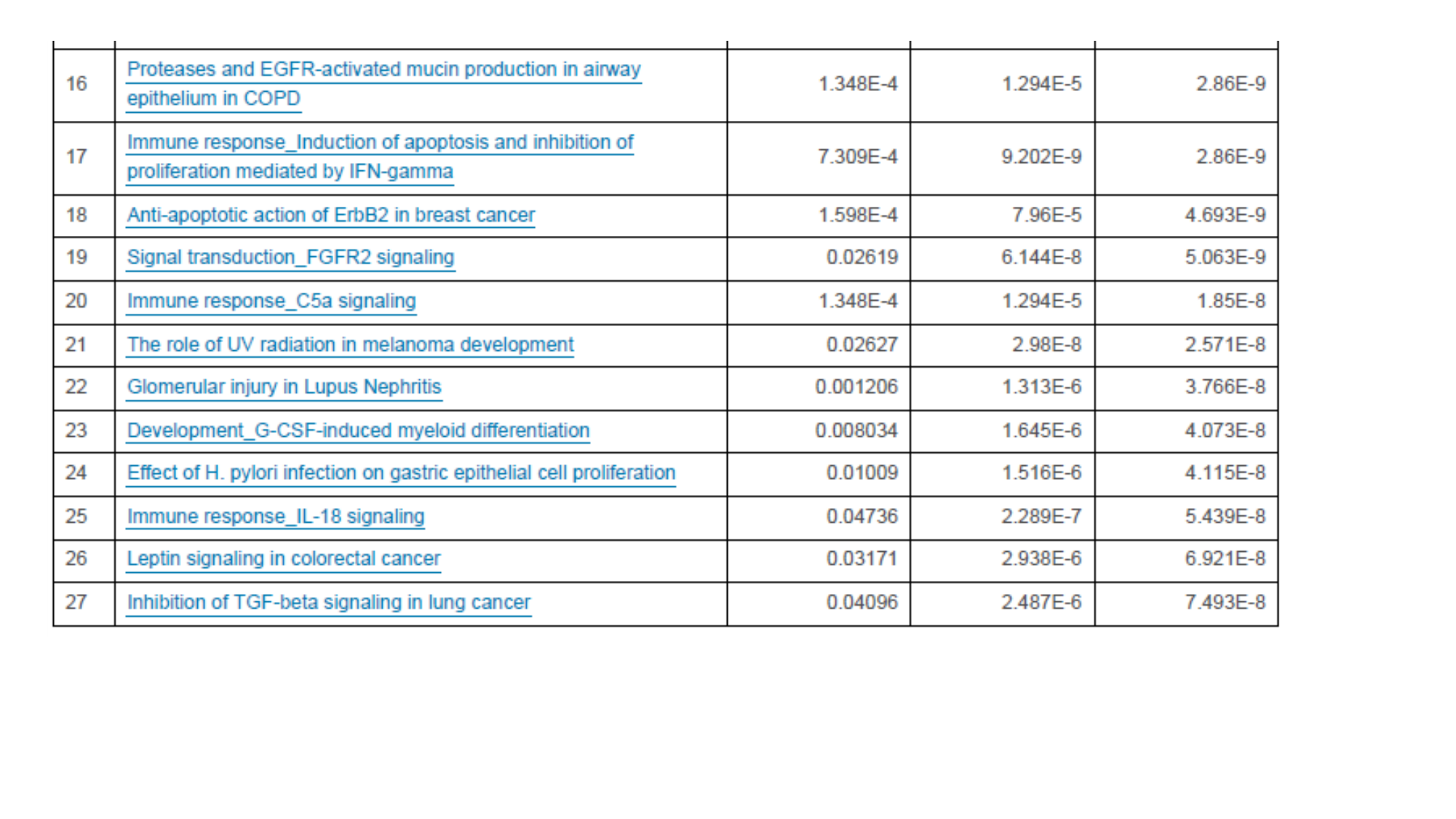

## Slide 19
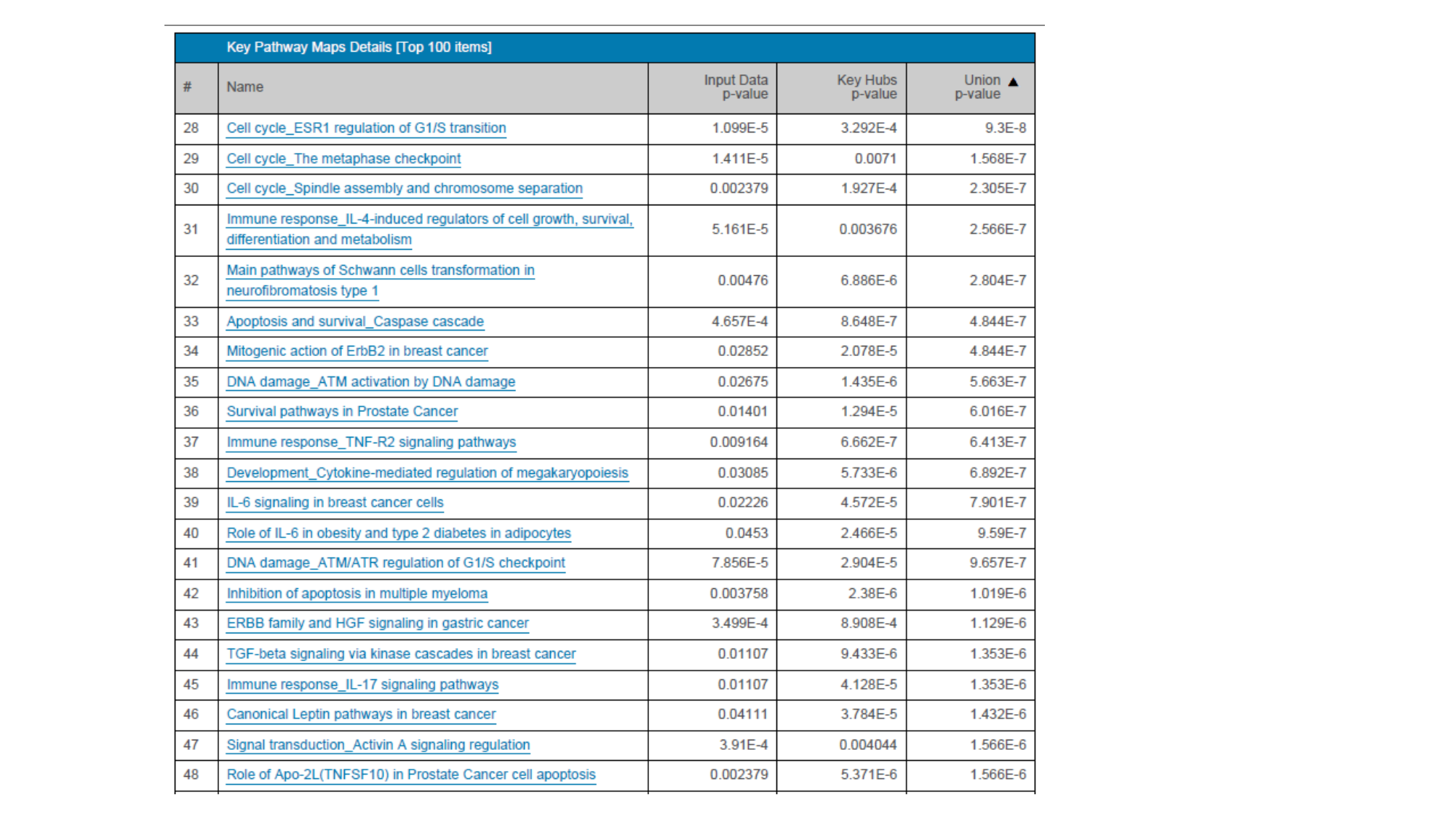

## Slide 20
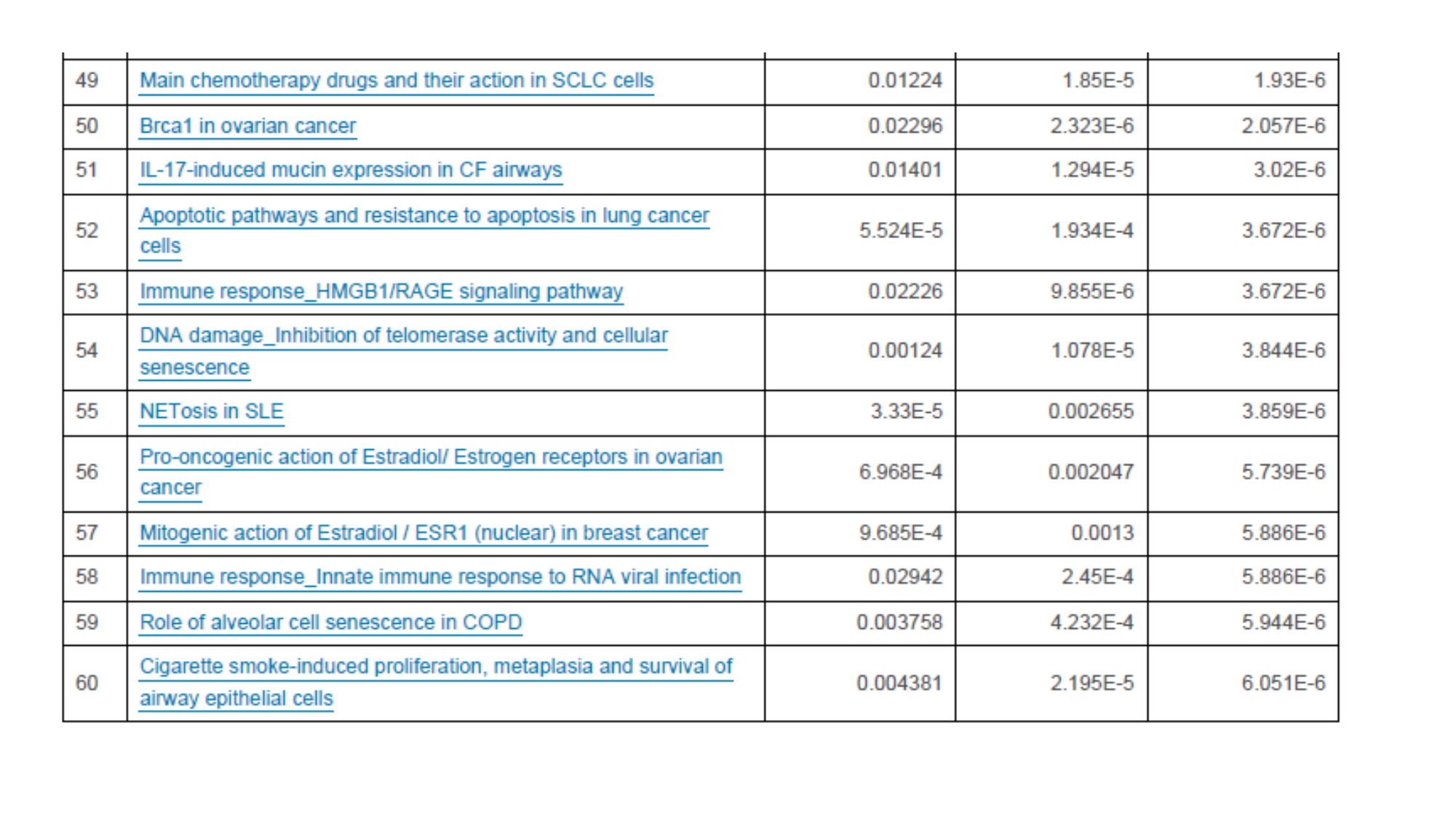

## Slide 21
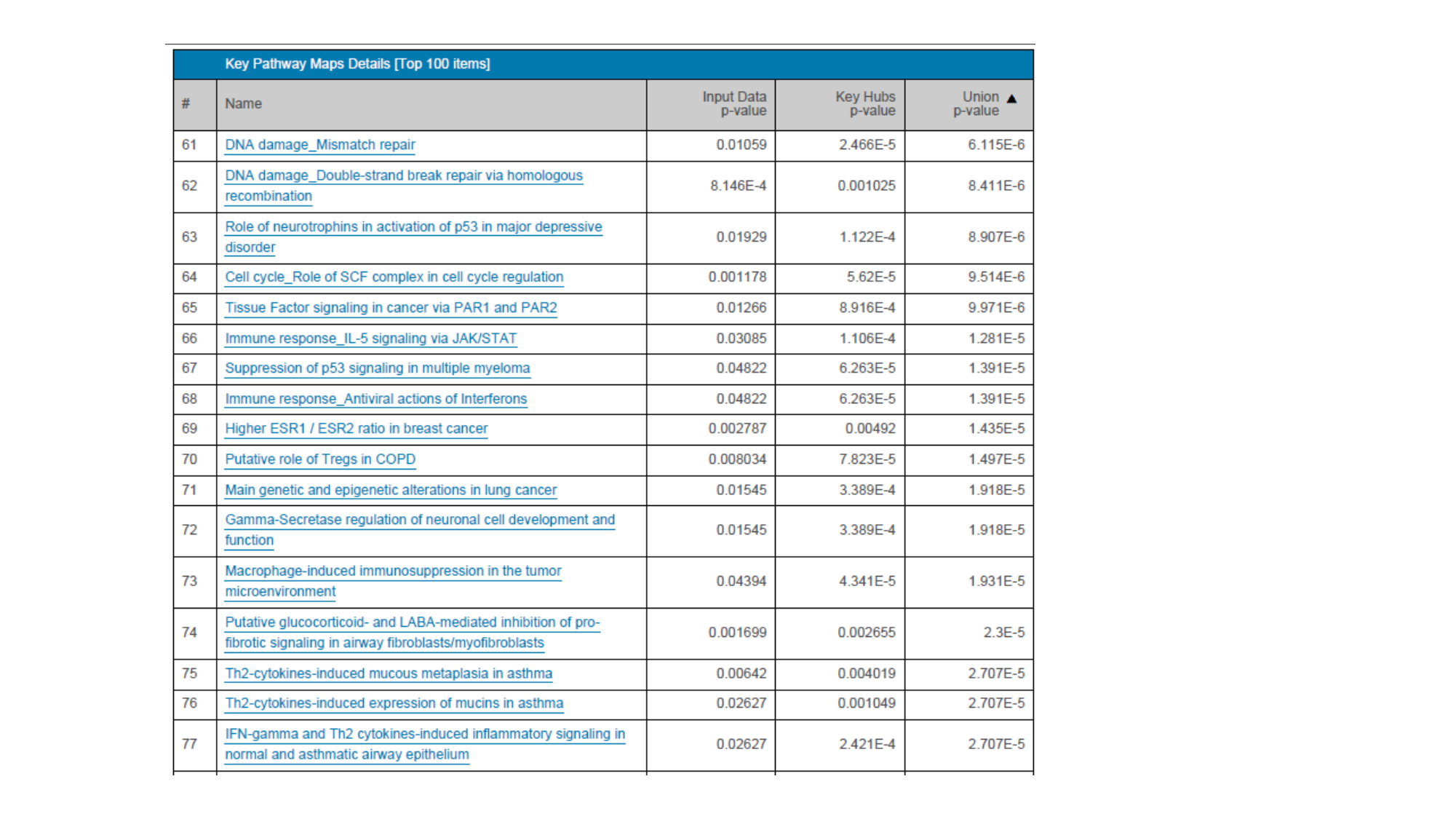

## Slide 22
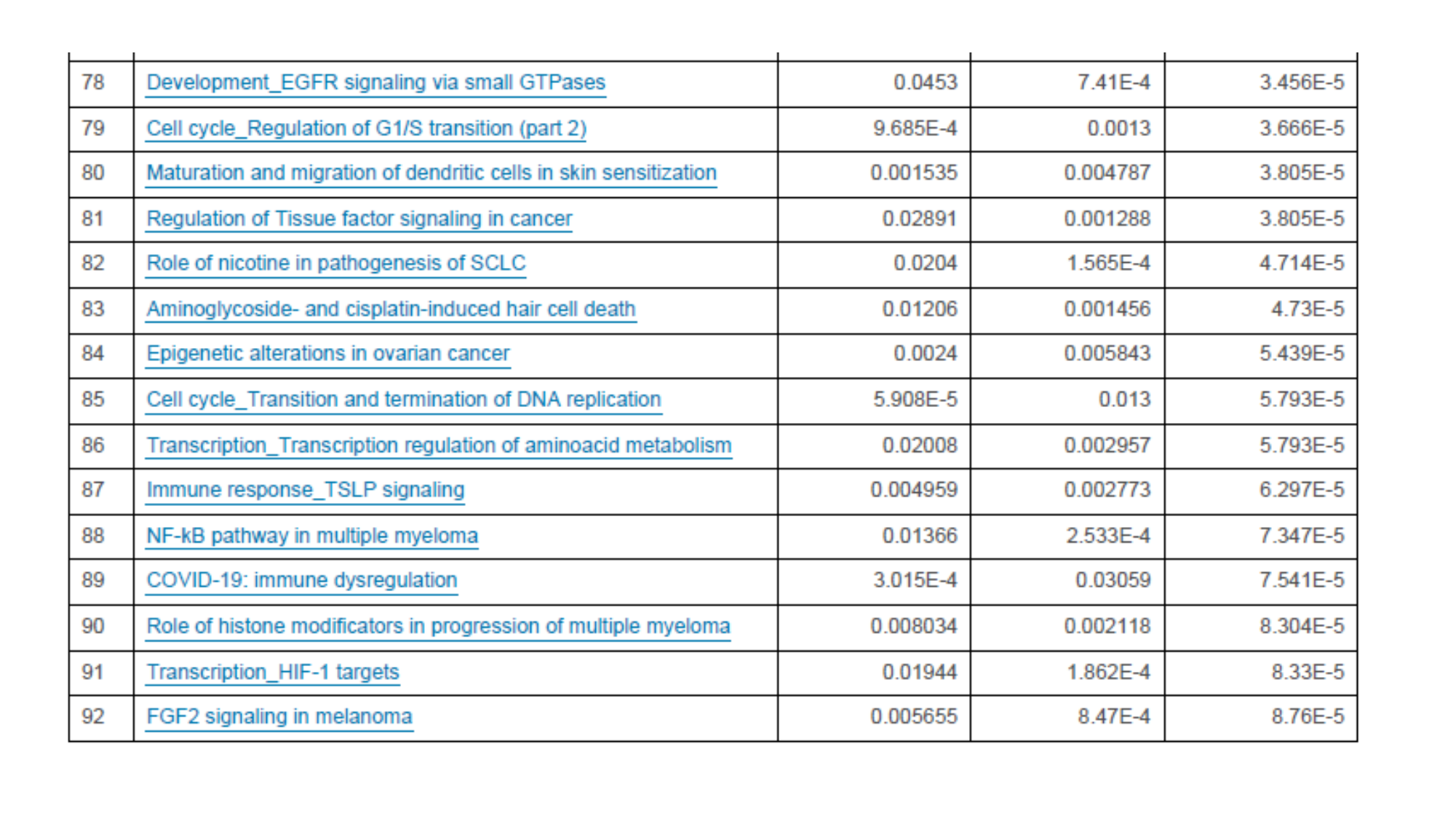

## Slide 23
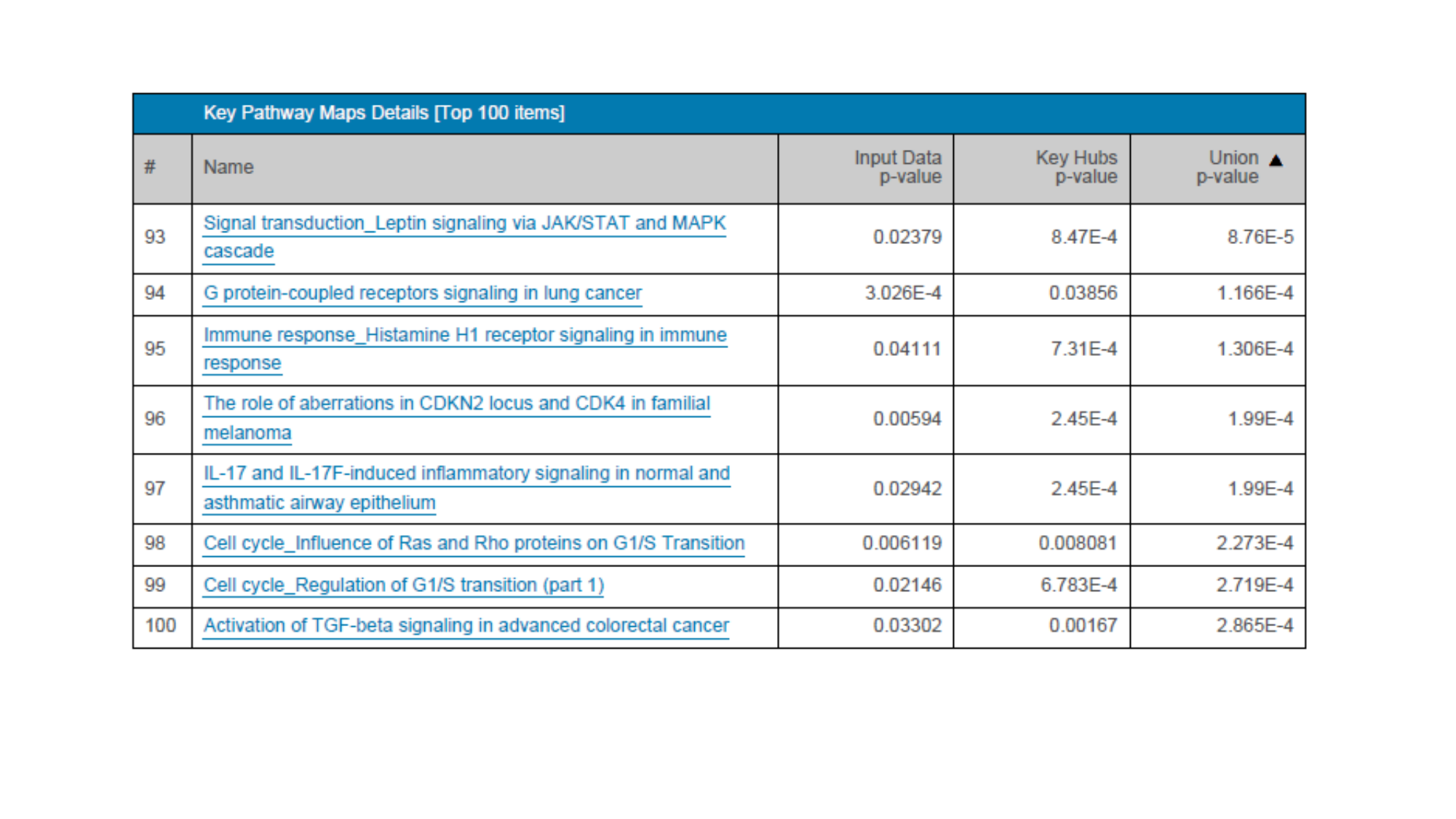

## Slide 24
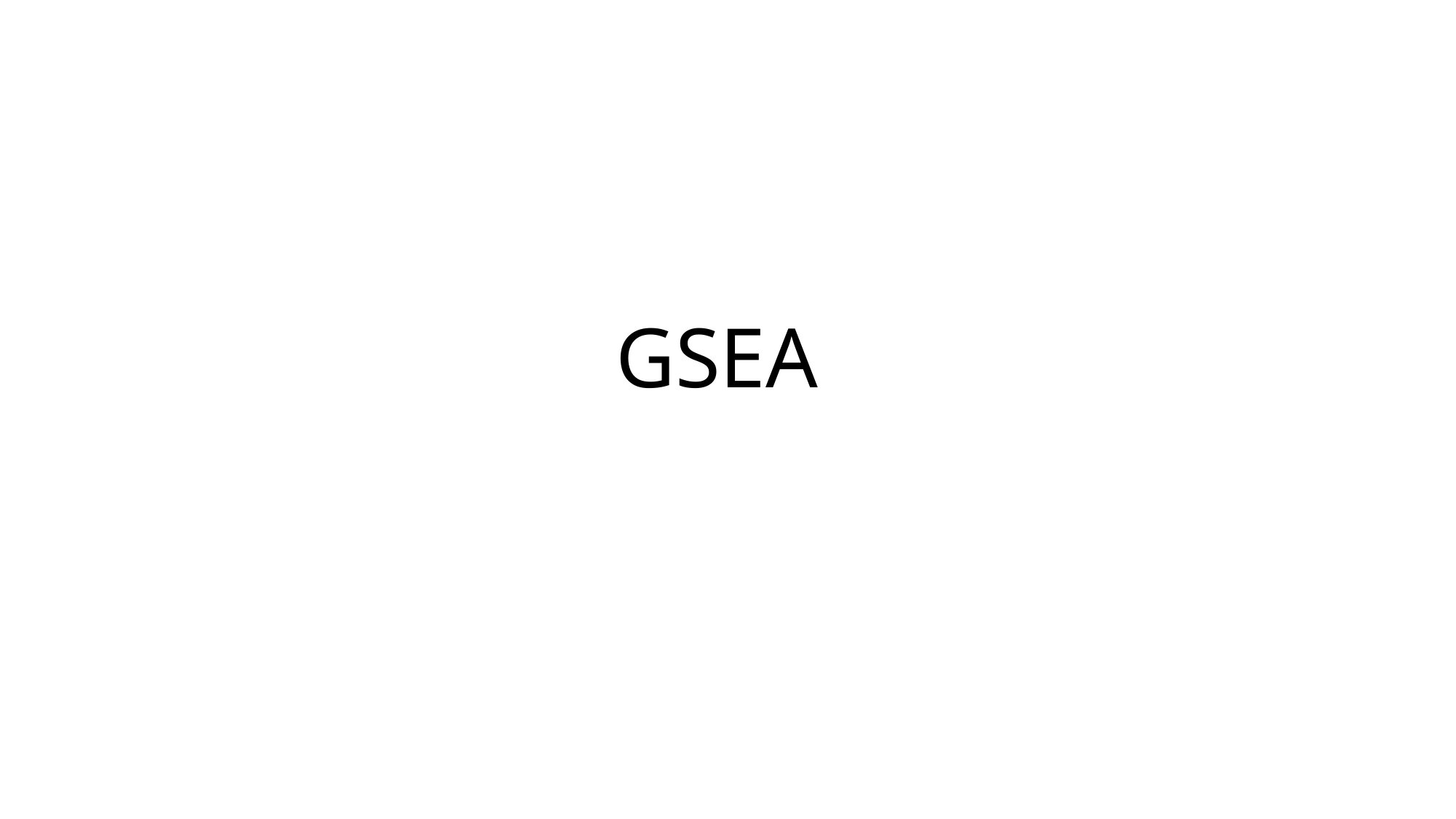

# GSEA

## Slide 25
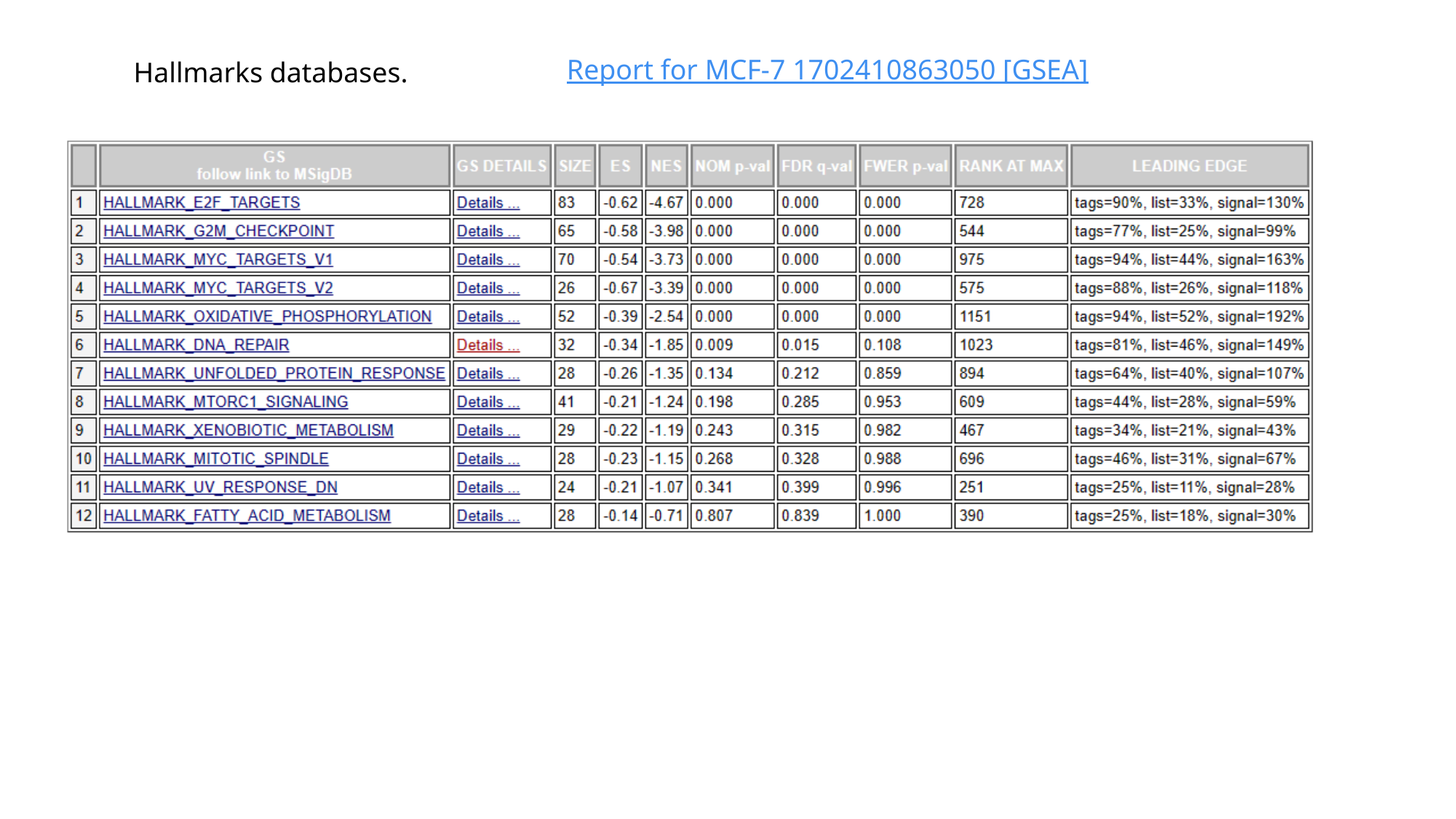

Report for MCF-7 1702410863050 [GSEA]
Hallmarks databases.

## Slide 26
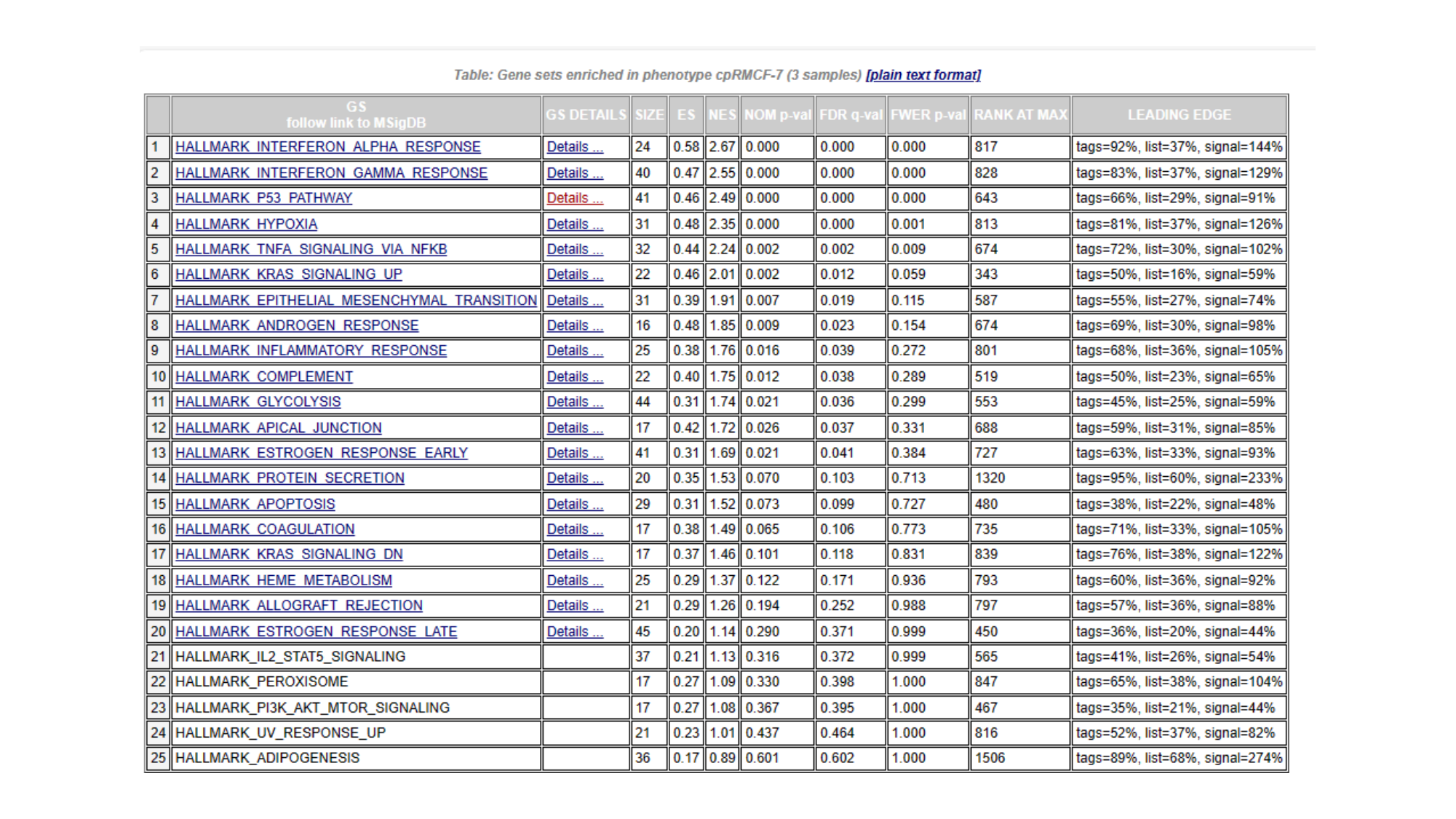

## Slide 27
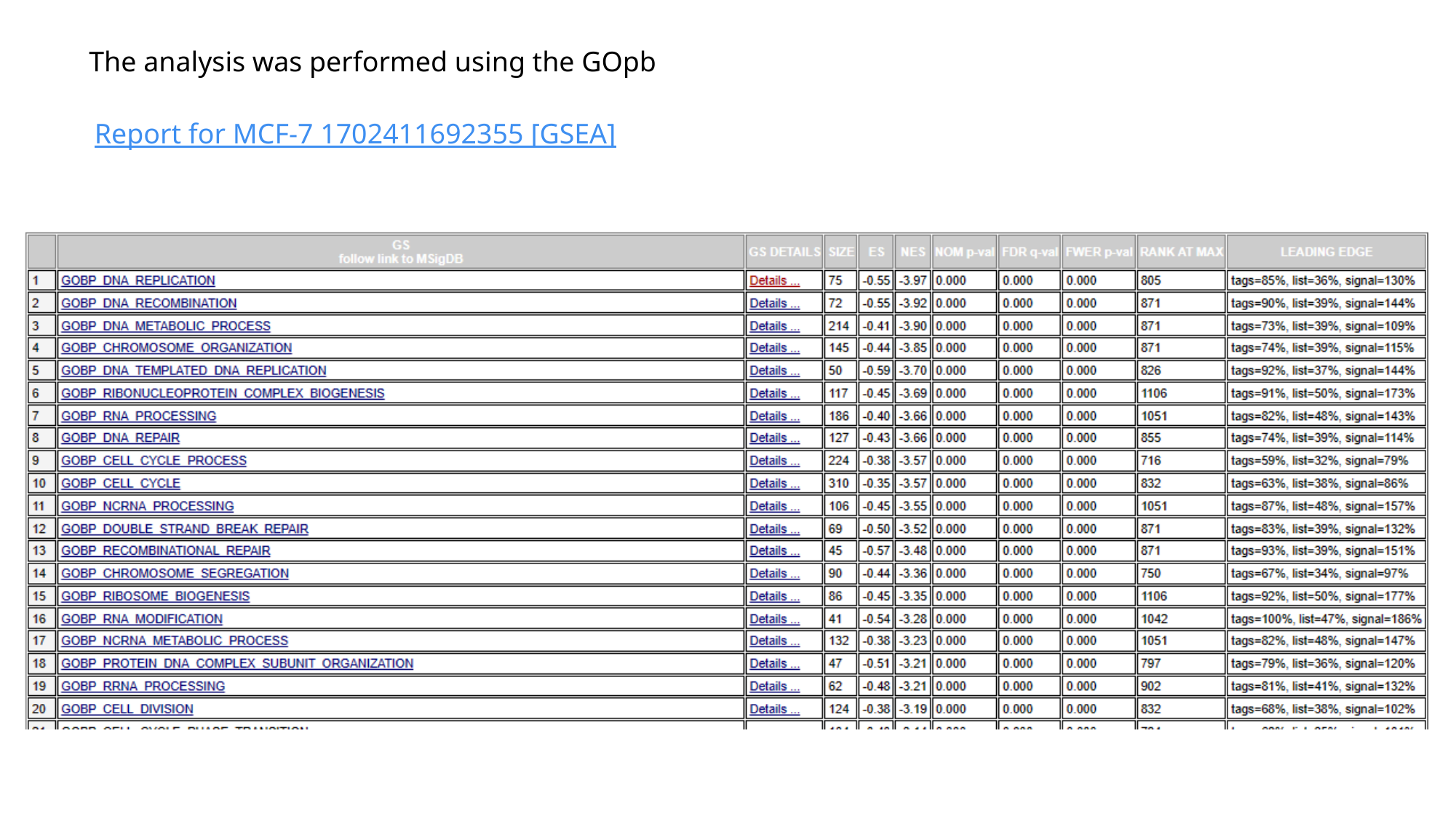

The analysis was performed using the GOpb
Report for MCF-7 1702411692355 [GSEA]

## Slide 28
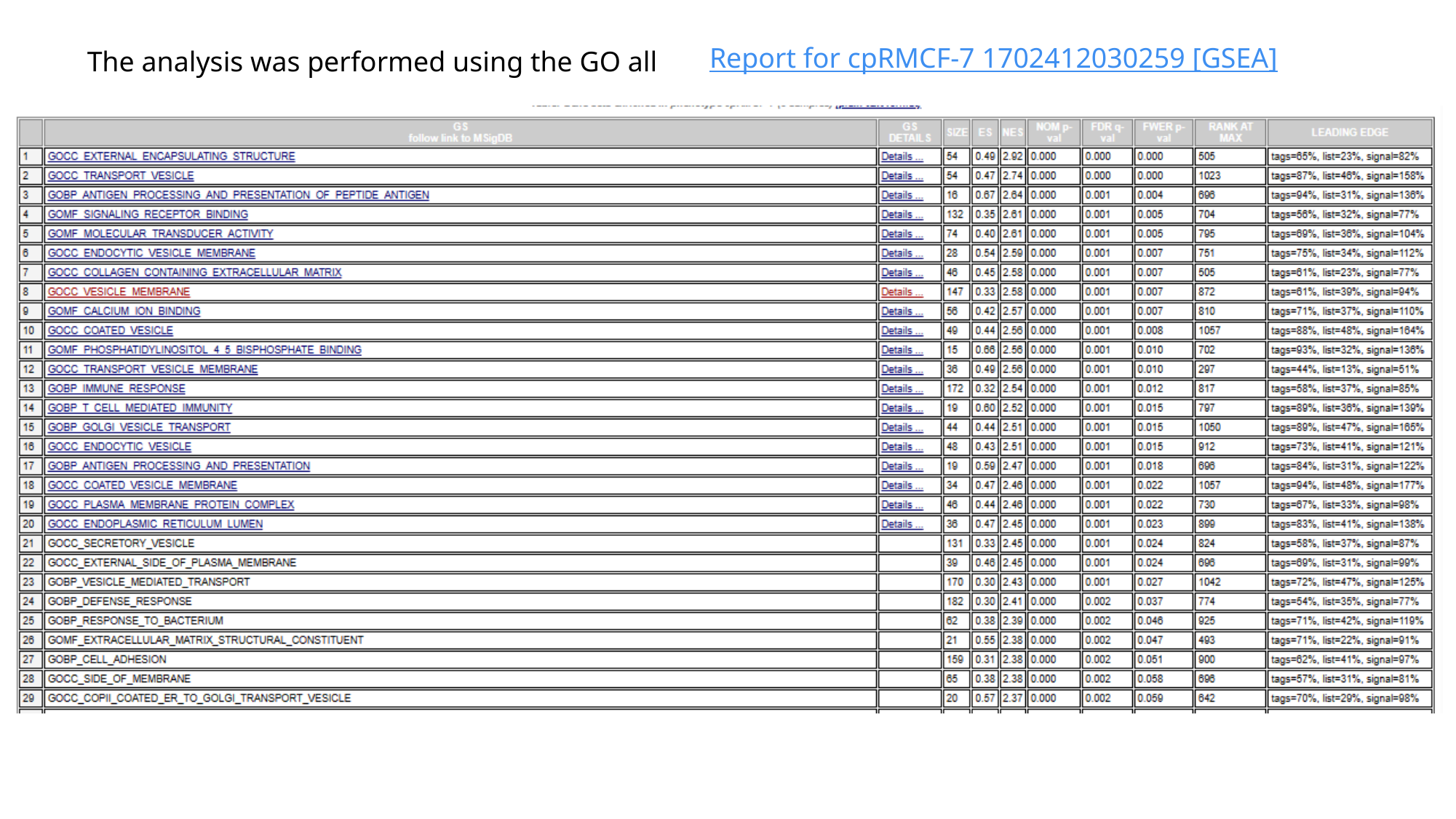

Report for cpRMCF-7 1702412030259 [GSEA]
The analysis was performed using the GO all

## Slide 29
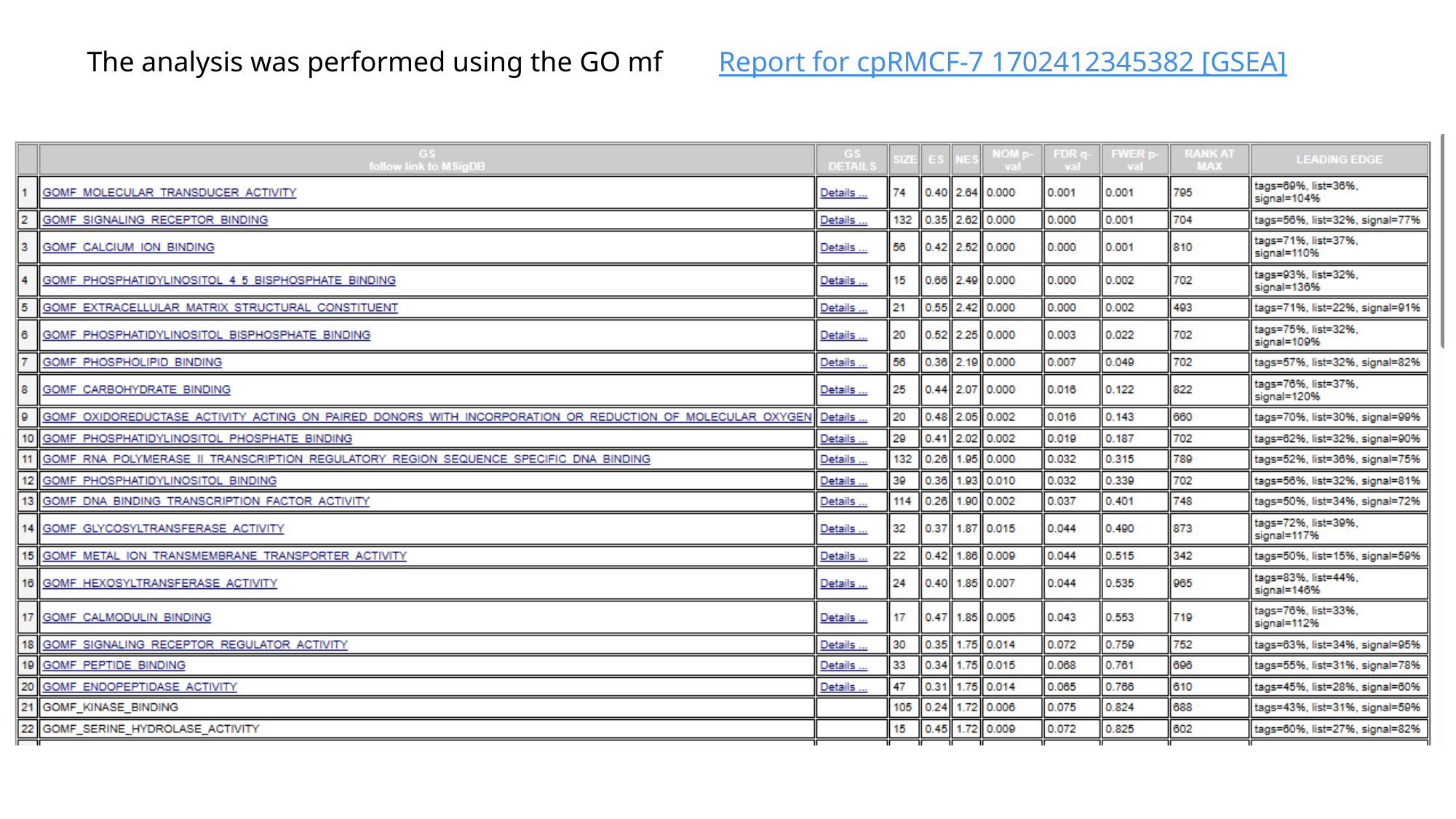

The analysis was performed using the GO mf
Report for cpRMCF-7 1702412345382 [GSEA]

## Slide 30
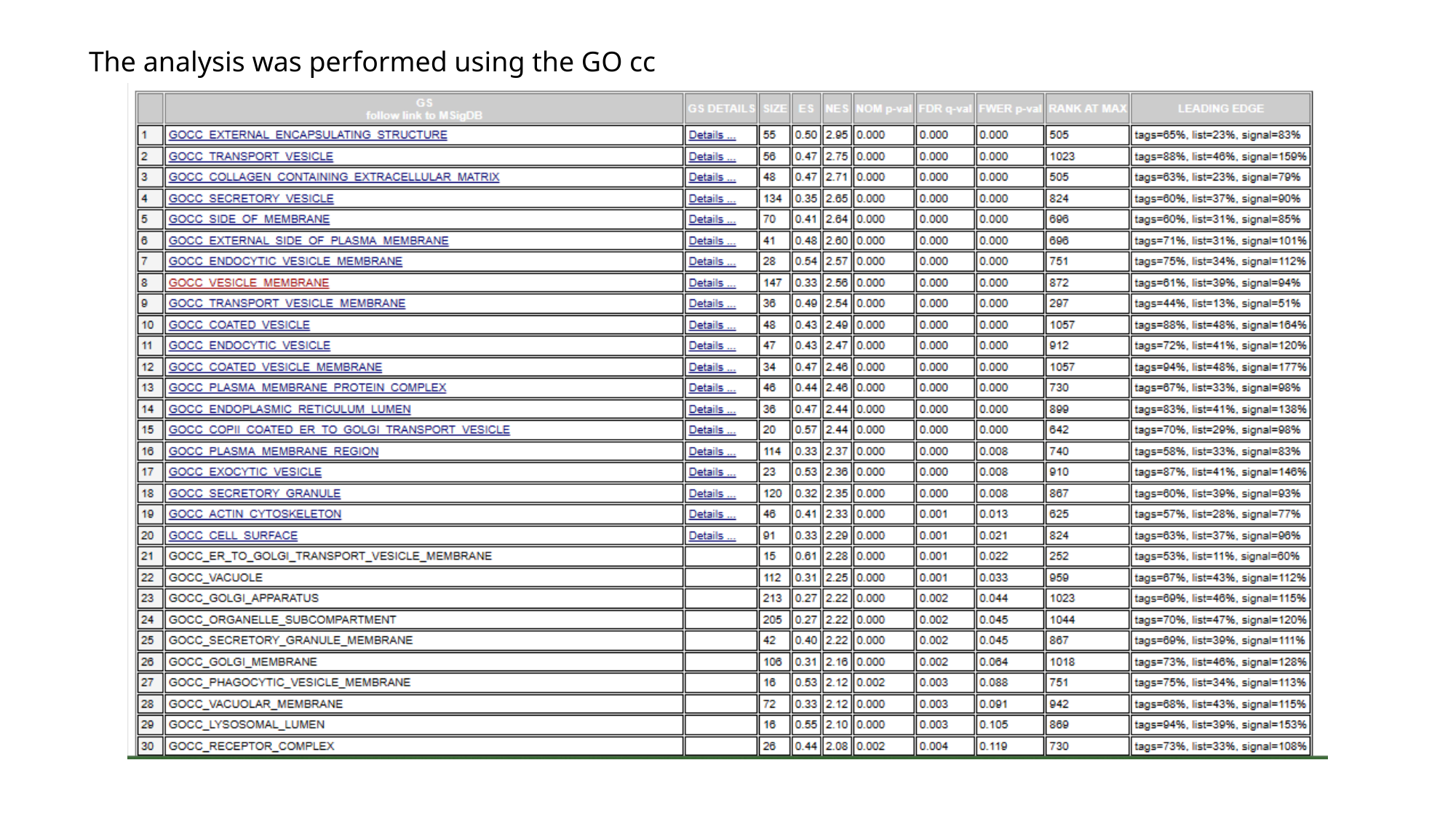

The analysis was performed using the GO cc

## Slide 31
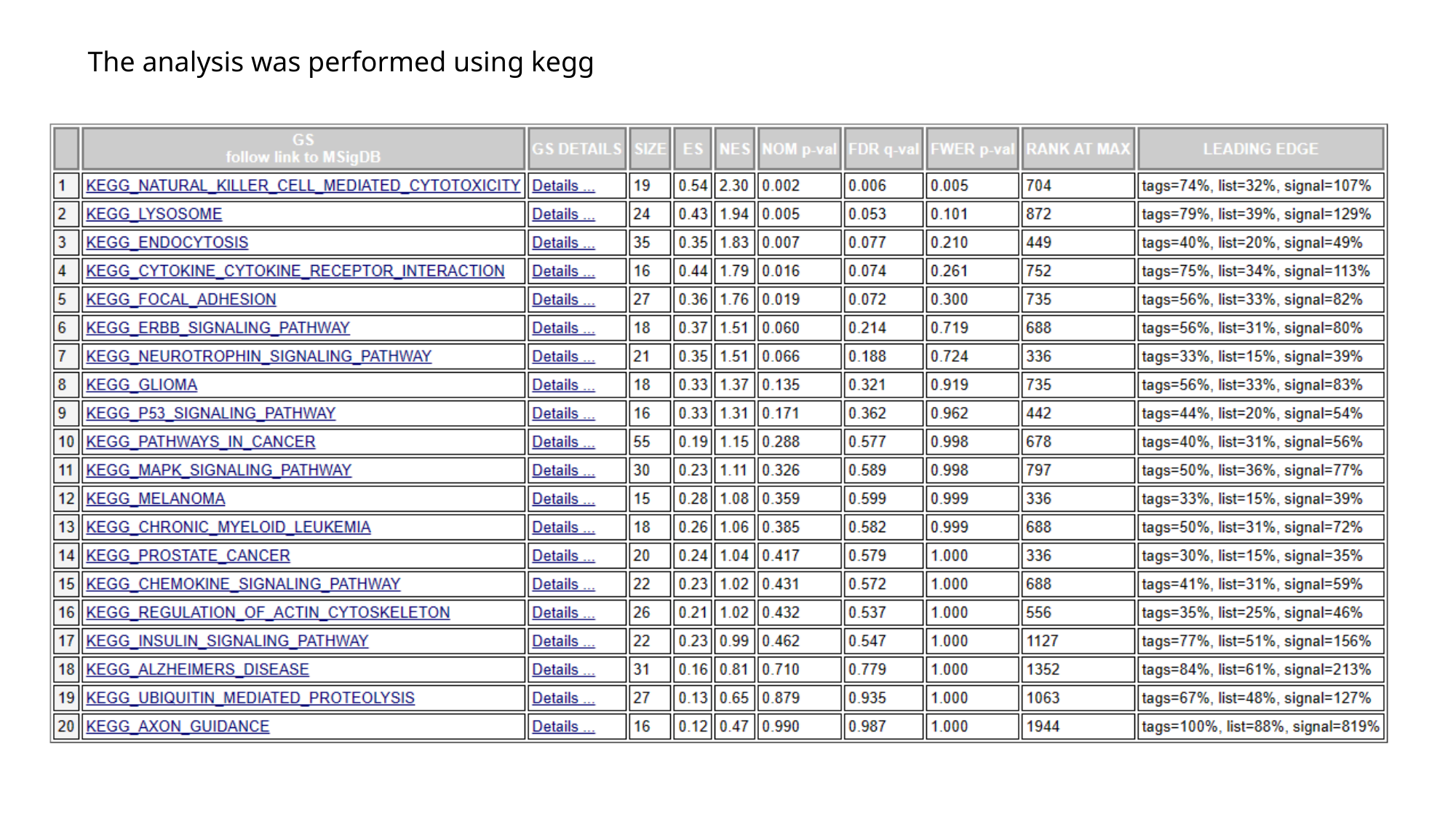

The analysis was performed using kegg
