# Supplementary material for: Transcriptomic Changes in Cisplatin-Resistant MCF-7 Cells
Source: Int J Mol Sci. 2024 Mar 29;25(7):3820. doi: 10.3390/ijms25073820 (PMC11011657; doi:10.3390/ijms25073820)
Supplement: Supplementary file 1 [file ijms-25-03820-s001.zip › ijms-2687107-supplementary additions/RNA.pdf]

Assay Class: Eukaryote Total RNA Nano
Data Path: D:\...Eukaryote Total RNA Nano_DE34903975_2018-05-03_12-29-51.xad

Created: 5/3/2018 12:29:50 PM
Modified: 11/27/2018 10:12:01 AM

Electrophoresis File Run Summary

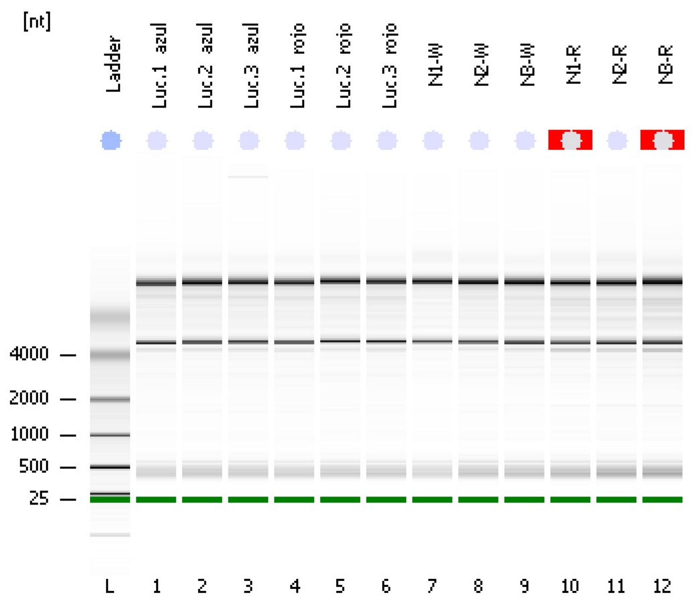

Instrument Information:

Instrument Name: DE34903975 Firmware: C.01.069
Serial#: DE34903975 Type: G2938C

Assay Information:

Assay Origin Path: D:\Programas\assays\RNA\Eukaryote Total RNA Nano Series II.xsy

Assay Class: Eukaryote Total RNA Nano

Version: 2.6

Assay Comments: Total RNA Analysis ng sensitivity (Eukaryote)

© Copyright 2003 - 2009 Agilent Technologies, Inc.

Chip Information:

Chip Lot #:

Reagent Kit Lot #:

Chip Comments:

Luc.1_azul
RIN: 8.70

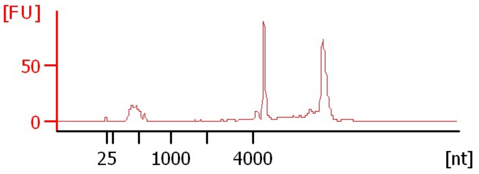

Luc.2_azul
RIN:9

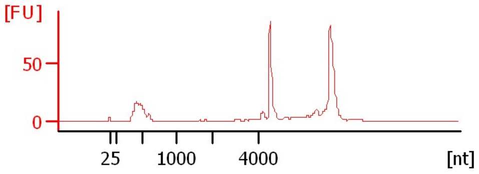

Luc.3_azul
RIN: 8.90

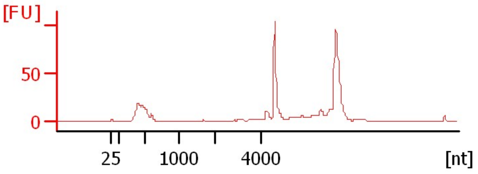

Luc.1_rojo
RIN: 8.80

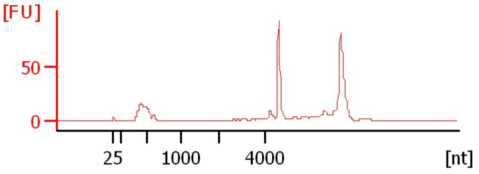

Luc.2_rojo
RIN:9

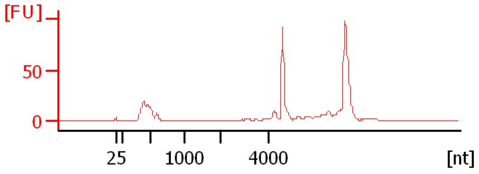

Luc.3_rojo
RIN: 8.80

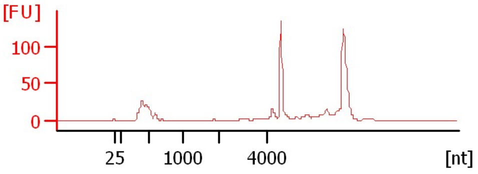

N1-W
RIN: 9.50

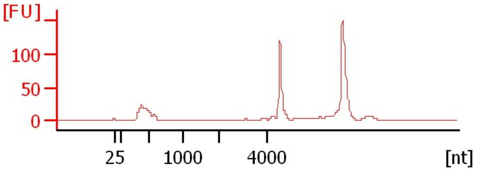

N2-W
RIN:9

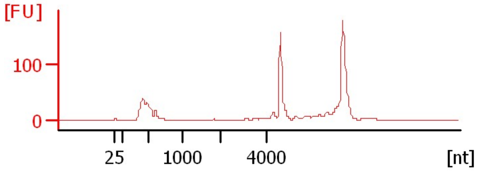

N3-W
RIN: 8.90

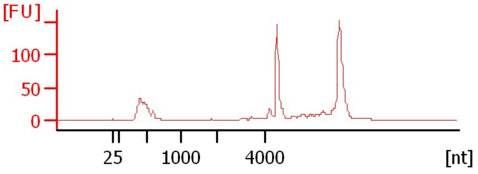

N1-R
RIN N/A

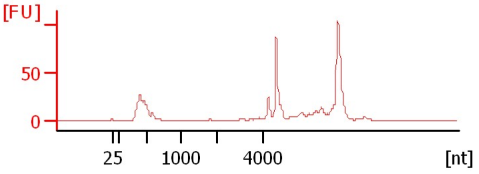

N2-R
RIN: 8.50

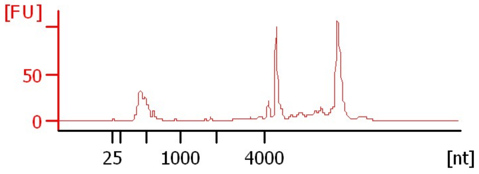

N3-R
RIN N/A

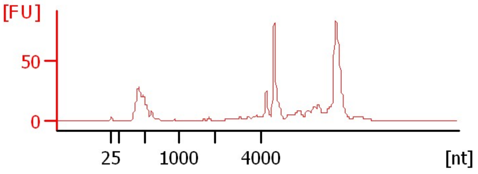

Assay Class: Eukaryote Total RNA Nano
Data Path: D:\...Eukaryote Total RNA Nano_DE34903975_2018-05-03_12-29-51.xad
Created: 5/3/2018 12:29:50 PM
Modified: 11/27/2018 10:12:01 AM

Electrophoresis File Run Summary (Chip Summary)

Sample Name	Sample Comment	Status	Result Label	Result Color
Luc.1_azul		✔	RIN: 8.70	
Luc.2_azul		✔	RIN:9	
Luc.3_azul		✔	RIN: 8.90	
Luc.1_rojo		✔	RIN: 8.80	
Luc.2_rojo		✔	RIN:9	
Luc.3_rojo		✔	RIN: 8.80	
N1-W		✔	RIN: 9.50	
N2-W		✔	RIN:9	
N3-W		✔	RIN: 8.90	
N1-R		✔	RIN N/A	
N2-R		✔	RIN: 8.50	
N3-R		✔	RIN N/A	
Ladder		✔	All Other Samples	

Chip Lot #

Reagent Kit Lot #

Chip Comments :

© Copyright 2003 - 2015 Agilent Technologies, Inc.

Assay Class:	Eukaryote Total RNA Nano	Created:	5/3/2018 12:29:50 PM
Data Path:	D:\...Eukaryote Total RNA Nano_DE34903975_2018-05-03_12-29-51.xad	Modified:	11/27/2018 10:12:01 AM

Electrophoresis Assay Details

General Analysis Settings

Number of Available Sample and Ladder Wells (Max.) : 13
Minimum Visible Range [s] : 17
Maximum Visible Range [s] : 70
Start Analysis Time Range [s] : 19
End Analysis Time Range [s] : 69
Ladder Concentration [ng/µl] : 150
Lower Marker Concentration [ng/µl] : 0
Upper Marker Concentration [ng/µl] : 0
Used Lower Marker for Quantitation
Standard Curve Fit is Logarithmic
Show Data Aligned to Lower Marker

Integrator Settings

Integration Start Time [s] : 19
Integration End Time [s] : 69
Slope Threshold : 0.6
Height Threshold [FU] : 0.5
Area Threshold : 0.2
Width Threshold [s] : 0.5
Baseline Plateau [s] : 6

Filter Settings

Filter Width [s] : 0.5
Polynomial Order : 4

Ladder

Ladder Peak	Size
1	25
2	200
3	500
4	1000
5	2000
6	4000

 © Copyright 2003 - 2015 Agilent Technologies, Inc.

Assay Class: Eukaryote Total RNA Nano
Data Path: D:\...Eukaryote Total RNA Nano_DE34903975_2018-05-03_12-29-51.xad

Created: 5/3/2018 12:29:50 PM
Modified: 11/27/2018 10:12:01 AM

Electropherogram Summary

Ladder

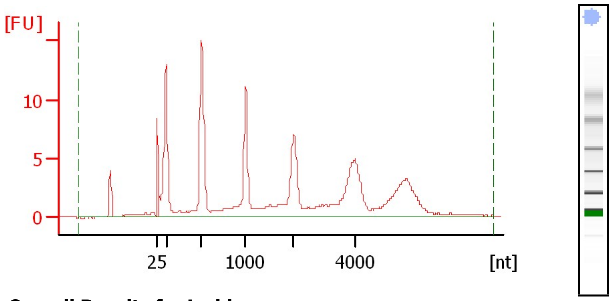

Overall Results for Ladder

RNA Area:	147.4
RNA Concentration:	150 ng/µl
Result Flagging Color:	
Result Flagging Label:	All Other Samples

N1-W

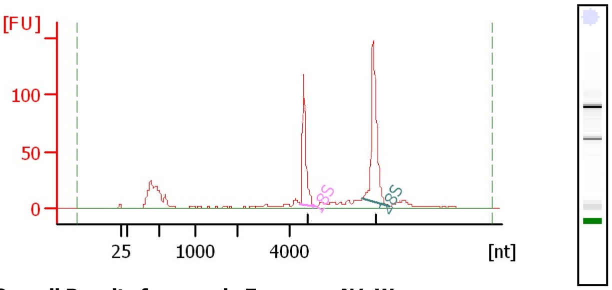

Overall Results for sample 7 : N1-W

RNA Area:	595.0
RNA Concentration:	605 ng/µl
rRNA Ratio [28s / 18s]:	1.7
RNA Integrity Number (RIN):	9.5 (B.02.08)
Result Flagging Color:	
Result Flagging Label:	RIN: 9.50

Fragment table for sample 7 : N1-W

Name	Start Size [nt]	End Size [nt]	Area	% of total Area
18S	4,425	5,003	110.2	18.5
28S	6,798	7,808	188.3	31.6

N2-W

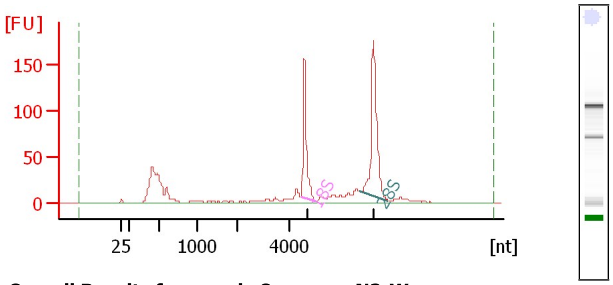

Overall Results for sample 8 : N2-W

RNA Area:	902.9
RNA Concentration:	919 ng/µl
rRNA Ratio [28s / 18s]:	1.7
RNA Integrity Number (RIN):	9 (B.02.08)
Result Flagging Color:	
Result Flagging Label:	RIN:9

Fragment table for sample 8 : N2-W

Name	Start Size [nt]	End Size [nt]	Area	% of total Area
18S	4,407	4,970	144.9	16.0
28S	6,673	7,749	239.2	26.5

N3-W

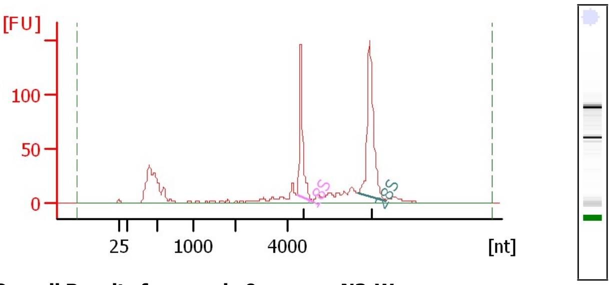

Overall Results for sample 9 : N3-W

RNA Area:	818.2
RNA Concentration:	833 ng/µl
rRNA Ratio [28s / 18s]:	1.6
RNA Integrity Number (RIN):	8.9 (B.02.08)
Result Flagging Color:	
Result Flagging Label:	RIN: 8.90

Fragment table for sample 9 : N3-W

Name	Start Size [nt]	End Size [nt]	Area	% of total Area
18S	4,387	4,951	138.9	17.0
28S	6,694	7,791	222.9	27.2

 © Copyright 2003 - 2015 Agilent Technologies, Inc.

Assay Class: Eukaryote Total RNA Nano
Data Path: D:\...Eukaryote Total RNA Nano_DE34903975_2018-05-03_12-29-51.xad

Created: 5/3/2018 12:29:50 PM
Modified: 11/27/2018 10:12:01 AM

Electropherogram Summary Continued ...

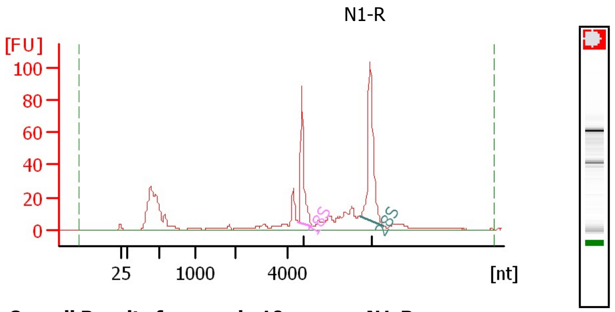

Overall Results for sample 10 : N1-R

RNA Area:	568.7
RNA Concentration:	579 ng/µl
rRNA Ratio [28s / 18s]:	1.6
RNA Integrity Number (RIN):	N/A (B.02.08)
Result Flagging Color:	
Result Flagging Label:	RIN N/A

Fragment table for sample 10 : N1-R

Name	Start Size [nt]	End Size [nt]	Area	% of total Area
18S	4,385	4,934	84.3	14.8
28S	6,745	7,714	131.6	23.1

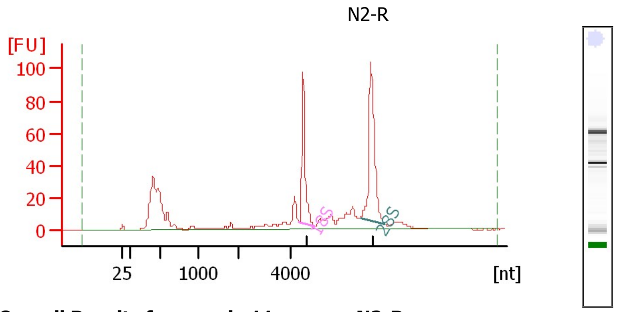

Overall Results for sample 11 : N2-R

RNA Area:	638.5
RNA Concentration:	650 ng/µl
rRNA Ratio [28s / 18s]:	1.5
RNA Integrity Number (RIN):	8.5 (B.02.08)
Result Flagging Color:	
Result Flagging Label:	RIN: 8.50

Fragment table for sample 11 : N2-R

Name	Start Size [nt]	End Size [nt]	Area	% of total Area
18S	4,380	4,948	92.3	14.5
28S	6,734	7,708	142.2	22.3

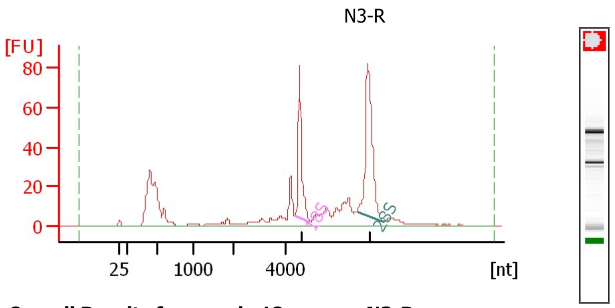

Overall Results for sample 12 : N3-R

RNA Area:	585.5
RNA Concentration:	596 ng/µl
rRNA Ratio [28s / 18s]:	1.7
RNA Integrity Number (RIN):	N/A (B.02.08)
Result Flagging Color:	
Result Flagging Label:	RIN N/A

Fragment table for sample 12 : N3-R

Name	Start Size [nt]	End Size [nt]	Area	% of total Area
18S	4,378	4,947	79.6	13.6
28S	6,753	7,777	133.7	22.8

© Copyright 2003 - 2015 Agilent Technologies, Inc.

Assay Class: Eukaryote Total RNA Nano
Data Path: D:\...Eukaryote Total RNA Nano_DE34903975_2018-05-03_12-29-51.xad

Created: 5/3/2018 12:29:50 PM
Modified: 11/27/2018 10:12:01 AM

Gel Image

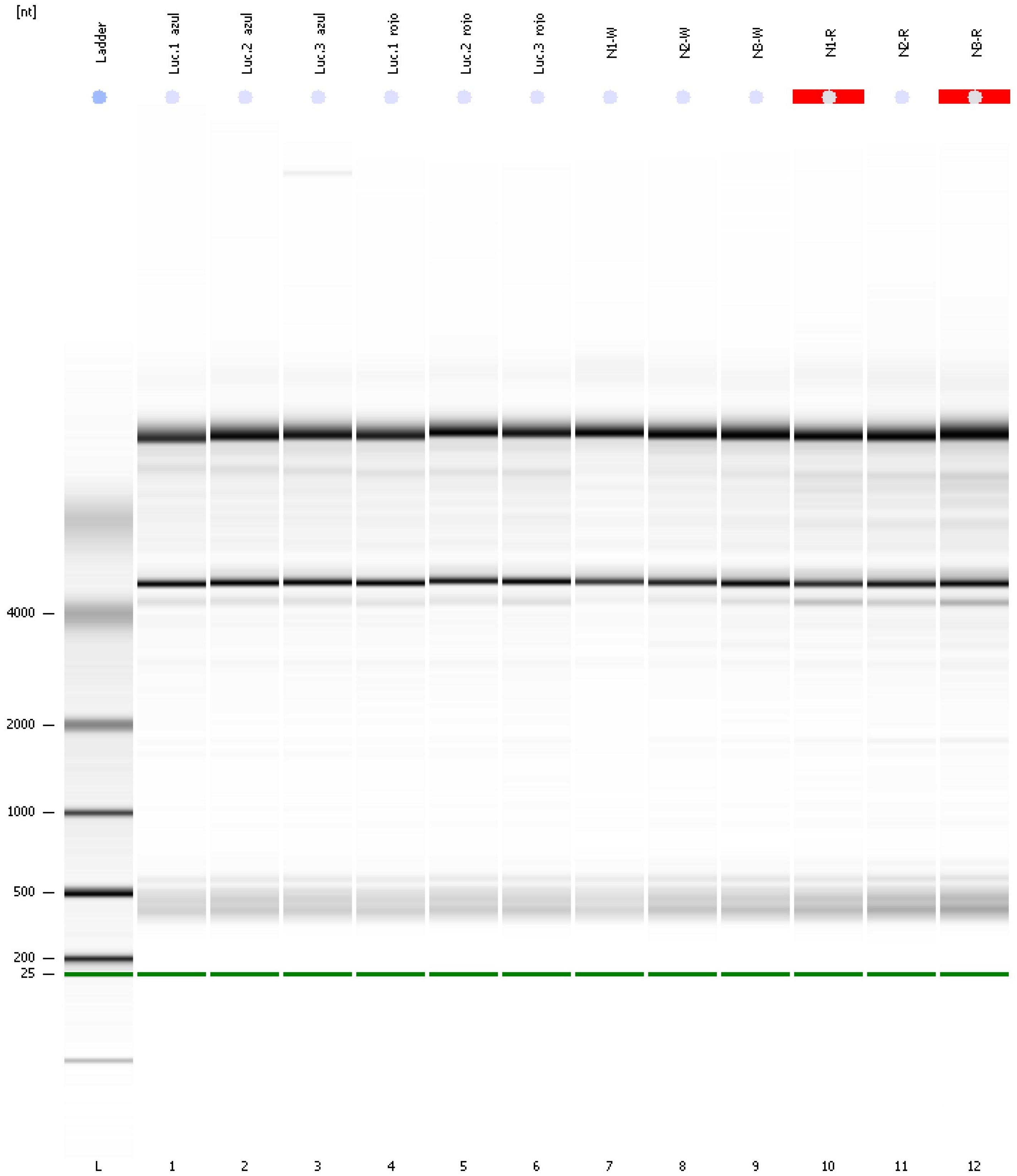

 © Copyright 2003 - 2015 Agilent Technologies, Inc.
