# Supplementary material for: Transcriptomic Changes in Cisplatin-Resistant MCF-7 Cells
Source: Int J Mol Sci. 2024 Mar 29;25(7):3820. doi: 10.3390/ijms25073820 (PMC11011657; doi:10.3390/ijms25073820)
Supplement: Supplementary file 1 [file ijms-25-03820-s001.zip › ijms-2687107-supplementary additions/SF1.pdf]

Instituto Nacional de Medicina Genómica
MEXICO

INSTITUTO NACIONAL DE MEDICINA GENÓMICA
Laboratorio de Diagnóstico Genómico
Periférico Sur 4809, Col. Arenal de Tepepan, Delegación Tlalpan,
México, D.F., C.P. 14610
Tel.: +52 (55) 53 50 19 37 www.inmegen.gob.mx

Dra. Vilma Araceli Maldonado Lagunas
INSTITUTO NACIONAL DE MEDICINA GENÓMICA
PRESENTE

Perfil Genético de STR para Verificar la Autenticidad de Línea Celular Humana

Fecha de la recepción de la última muestra: 30 mayo 2017, 13:41 horas   Resultado emitido el: 05 junio 2017, 12:08 horas

Nota: El personal del INMEGEN no obtuvo las muestras, ni verificó la identidad de las mismas. El INMEGEN es responsable del resultado reportado bajo la información proporcionada. La veracidad de la información proporcionada o la autenticidad de las muestras es responsabilidad de la persona que ha firmado el Documento de Autorización.
Metodología: La extracción de DNA se realizó a partir de la muestra proporcionada empleando la metodología adecuada según el tipo de muestra entregada (Maxwell 16 de Promega, QIAamp DNA Blood Mini Kit de QIAGEN, Prep-n-go buffer de AB o FTA Purification Reagent de GE). Reacción en cadena de la polimerasa tipo multiplex y electroforesis capilar en el Analizador Genético 3500 de AB, análisis de fragmentos en Gene Mapper ID v5
Realizaron el estudio: Itsel Alejandrina Alva Velázquez, Anallely Muñoz Rivas y Beatriz E. Villegas Torres

RESULTADOS:

LÍNEA CELULAR	MCF7					
ID TUBO	MCF7					
NO. CASO LDG	LCH417-334			PERFIL GENÉTICO PREVIAMENTE REPORTADO PARA:		
ID MUESTRA LDG	LCH417-334-1					
**Marcador**	**PERFIL GENÉTICO OBTENIDO:**			**Marcador**	**MCF7 (ATCC® HTB-22™)**	
**D3S1358**	16	18.1				
**vWA**	14	15		**vWA**	14	15
**D16S539**	11	12		**D16S539**	11	12
**CSF1PO**	10	10		**CSF1PO**	10	10
**TPOX**	9	12		**TPOX**	9	12
**D8S1179**	10	14				
**D21S11**	30	30				
**D18S51**	14	14				
**D2S441**	10	14				
**D19S433**	10	13	14			
**THO1**	6	6		**THO1**	6	6
**FGA**	23	24	25			
**D22S1045**	15	16				
**D5S818**	11	12		**D5S818**	11	12
**D13S317**	11	11		**D13S317**	11	11
**D7S820**	8	9		**D7S820**	8	9
**SE33**	16	18				
**D10S1248**	14	14				
**D1S1656**	11	15.3				
**D12S391**	18	20				
**D2S1338**	21	23				
**DYS391**	No aplica					
**AMEL**	X	X		**AMEL**	X	X

referencia: https://www.atcc.org/products/all/HTB-22.aspx#specifications

LÍNEA CELULAR	AS49					
ID TUBO	AS49					
NO. CASO LDG	LCH417-334			PERFIL GENÉTICO PREVIAMENTE REPORTADO PARA:		
ID MUESTRA LDG	LCH417-334-2					
**Marcador**	**PERFIL GENÉTICO OBTENIDO:**			**Marcador**	**A549 (ATCC® CCL-185™)**	
**D3S1358**	16	16				
**vWA**	14	14		**vWA**	**14**	**14**
**D16S539**	11	12		**D16S539**	**11**	**12**
**CSF1PO**	10	12		**CSF1PO**	**10**	**12**
**TPOX**	8	11		**TPOX**	**8**	**11**
**D8S1179**	13	14				
**D21S11**	29	29				
**D18S51**	14	17				
**D2S441**	10	13				
**D19S433**	13	13				
**THO1**	8	9.3		**THO1**	**8**	**9.3**
**FGA**	23	23				
**D22S1045**	15	15				
**D5S818**	11	11		**D5S818**	**11**	**11**
**D13S317**	11	11		**D13S317**	**11**	**11**
**D7S820**	8	11		**D7S820**	**8**	**11**
**SE33**	19	25.2				
**D10S1248**	13	16				
**D1S1656**	17	18.3				
**D12S391**	18	18				
**D2S1338**	24	24				
**DYS391**	No aplica					
**AMEL**	X	X		**AMEL**	X	Y

referencia: https://www.atcc.org/Products/All/CCL-185#specifications

LÍNEA CELULAR	Calu					
ID TUBO	AS49					
NO. CASO LDG	LCH417-334			PERFIL GENÉTICO PREVIAMENTE REPORTADO PARA:		
ID MUESTRA LDG	LCH417-334-3					
**Marcador**	**PERFIL GENÉTICO OBTENIDO:**			**Marcador**	**CALU1**	
**D3S1358**	17	17				
**vWA**	15	16		**vWA**	**15**	**16**
**D16S539**	11	11		**D16S539**	**11**	**11**
**CSF1PO**	10	10		**CSF1PO**	**10**	**10**
**TPOX**	8	8		**TPOX**	**8**	**8**
**D8S1179**	10	10				
**D21S11**	28	28				
**D18S51**	14	17				
**D2S441**	10	11				
**D19S433**	13	15				
**THO1**	9.3	9.3		**THO1**	**9.3**	**9.3**
**FGA**	20	21				
**D22S1045**	15	15				
**D5S818**	10	12		**D5S818**	**10**	**12**
**D13S317**	11	12		**D13S317**	**11**	**12**
**D7S820**	9	10		**D7S820**	**9**	**10**
**SE33**	18	21				
**D10S1248**	13	13				
**D1S1656**	15	16.3				
**D12S391**	18	19	20			
**D2S1338**	16	17				
**DYS391**	No aplica					
**AMEL**	X	X		**AMEL**	X	X

referencia: http://www.phe-culturecollections.org.uk/products/celllines/generalcell/detail.jsp?refId=93120818&collection=ecacc_gc

INTERPRETACIÓN.

A partir de las muestras entregadas (ID LDG: LCH417-34), se realizó la lisis celular para amplificar 21 marcadores de tipo STR autosómicos, un STR en el cromosoma Y, además del gen de la amelogenina. En la electroforesis capilar se obtuvo un electroferograma de buena calidad que nombro sólo los alelos reportados en este documento.

La muestra identificada como: “MCF7” (ID LDG: LCH417-344-1) posee los mismos 14 alelos reportados para la línea celular humana MCF7 (ATCC® HTB-22™). Con esta información se puede concluir que la muestra “MCF7 (ID LDG: LCH417-344-1) contiene sólo células de la línea antes mencionada. No existe evidencia de que la muestra analizada presente más de dos tipos de células de origen humano es decir la muestra no esta contaminada con otro tipo de células humanas.

La muestra identificada como: “AS49” (ID LDG: LCH417-344-2) posee 14 de los 15 alelos reportados para la línea celular humana A549 (ATCC® CCL-185™). No existe evidencia de que la muestra analizada presente dos ó más tipos de células de origen humano.

La muestra identificada como: “Calu” (ID LDG: LCH417-344-3) posee los 13 alelos reportados para la línea celular humana CALU. No existe evidencia de que la muestra analizada presente más de dos tipos de células de origen humano es decir la muestra no esta contaminada.

Firmado digitalmente por Beatriz E. Villegas T.
Fecha: 2017.06.05 12:13:52 -05'00'

Mtra. Beatriz E. Villegas Torres, cédula profesional 3788745. En ausencia de la Dra. Carmen Aláez Versón, Jefa del Departamento de Diagnóstico Genómico, Dirección de Investigación, por el artículo cuarenta y cinco del Capítulo Décimo del Estatuto Orgánico del Instituto Nacional de Medicina Genómica

Dra. Carmen Aláez Verson

Cédula profesional: 4768536

Jefe del Departamento de Diagnóstico Genómico, Dirección de Investigación, ***INMEGEN.***

Este Laboratorio participa en el Programas de Control de Calidad del Colegio Americano de Patólogos con resultados satisfactorios en pruebas de identificación humana.
