# Supplementary material for: Transcriptomic Changes in Cisplatin-Resistant MCF-7 Cells
Source: Int J Mol Sci. 2024 Mar 29;25(7):3820. doi: 10.3390/ijms25073820 (PMC11011657; doi:10.3390/ijms25073820)
Supplement: Supplementary file 1 [file ijms-25-03820-s001.zip › ijms-2687107-supplementary additions/SNORA.pdf]

HUGO	Name	RealFC	PPEE	Location
SNORA11D	small nucleolar RNA, H/ACA box 11D	5.90	5.62E-05	Xp11.21
SNORA11	small nucleolar RNA, H/ACA box 11	3.32	0.00705456	Xp11.22
SNORA36B	small nucleolar RNA, H/ACA box 36B	2.69	0.00504844	1q41
SNORA66	small nucleolar RNA, H/ACA box 66	2.54	0.02502041	11q21
SNORA40	small nucleolar RNA, H/ACA box 40	2.11	0.04620136	1p22.1
SNORA78	small nucleolar RNA, H/ACA box 78	2.08	0.0156753	16p13.3
SNORD56	small nucleolar RNA, C/D box 56	1.98	0.01603528	20p13
SNRPA	small nuclear ribonucleoprotein polypeptide A	0.71	0.00038768	19q13.2
SNRPB	small nuclear ribonucleoprotein polypeptides B and B1	0.67	0.00157498	20p13
SNRPD2	small nuclear ribonucleoprotein D2 polypeptide	0.67	9.96E-13	18q11.2
SNRPE	small nuclear ribonucleoprotein polypeptide E	0.63	1.82E-07	19q13.2-q13.3
SNRPF	small nuclear ribonucleoprotein polypeptide F	0.60	1.65E-09	1q32.1
SNRPD1	small nuclear ribonucleoprotein D1 polypeptide	0.59	2.29E-09	12q23.1
