# Supplementary material for: Transcriptomic Changes in Cisplatin-Resistant MCF-7 Cells
Source: Int J Mol Sci. 2024 Mar 29;25(7):3820. doi: 10.3390/ijms25073820 (PMC11011657; doi:10.3390/ijms25073820)
Supplement: Supplementary file 1 [file ijms-25-03820-s001.zip › ijms-2687107-supplementary additions/trimming.html]

Trimming | Resistant cells RNASeq experiments


# Resistant cells RNASeq experiments

Analyses: July 14, 2018

## Site Navigation[Skip]

- Home
- Trimming
- Quality Control
- Align logs
- Results. Differential Expression

## Trimming

Conditions:

Trim adapter: TruSeq2-PE

Headcrop:1

Sliding window 4:20

Crop 75

MinLen 50

© Jorge Melendez Zajgla 2018

[Back To Top]
