# Supplementary material for: Transcriptomic Changes in Cisplatin-Resistant MCF-7 Cells
Source: Int J Mol Sci. 2024 Mar 29;25(7):3820. doi: 10.3390/ijms25073820 (PMC11011657; doi:10.3390/ijms25073820)
Supplement: Supplementary file 1 [file ijms-25-03820-s001.zip › ijms-2687107-supplementary additions/WT-CMF-7 N2_star_trim_gcrh38_rsem.pdf]

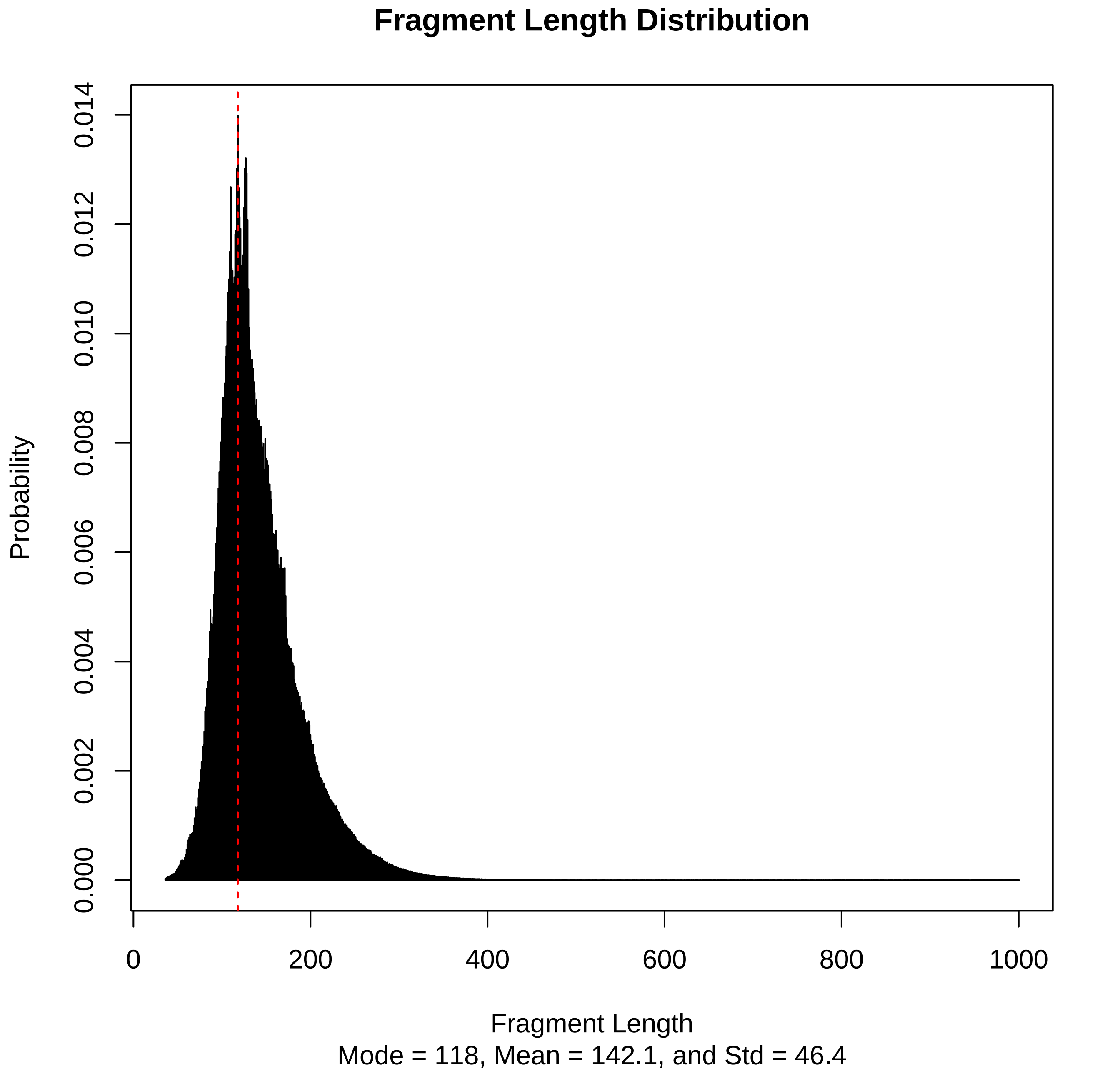
Fragment Length Distribution
Probability
0.000
0.002
0.004
0.006
0.008
0.010
0.012
0.014
0
200
400
600
800
1000
Fragment Length
Mode = 118, Mean = 142.1, and Std = 46.4

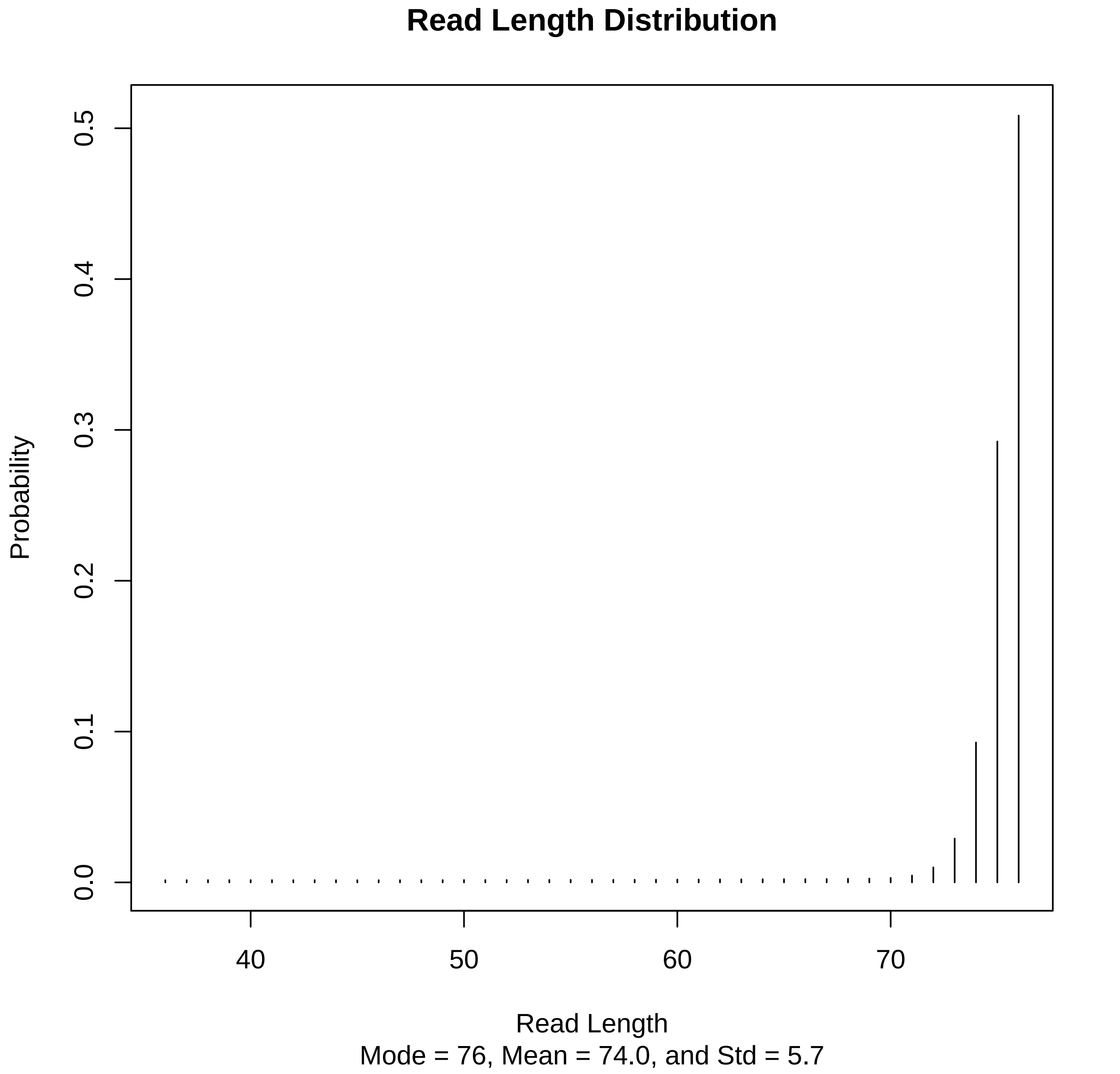
Read Length Distribution
Probability
0.0
0.1
0.2
0.3
0.4
0.5
40
50
60
70
Read Length
Mode = 76, Mean = 74.0, and Std = 5.7

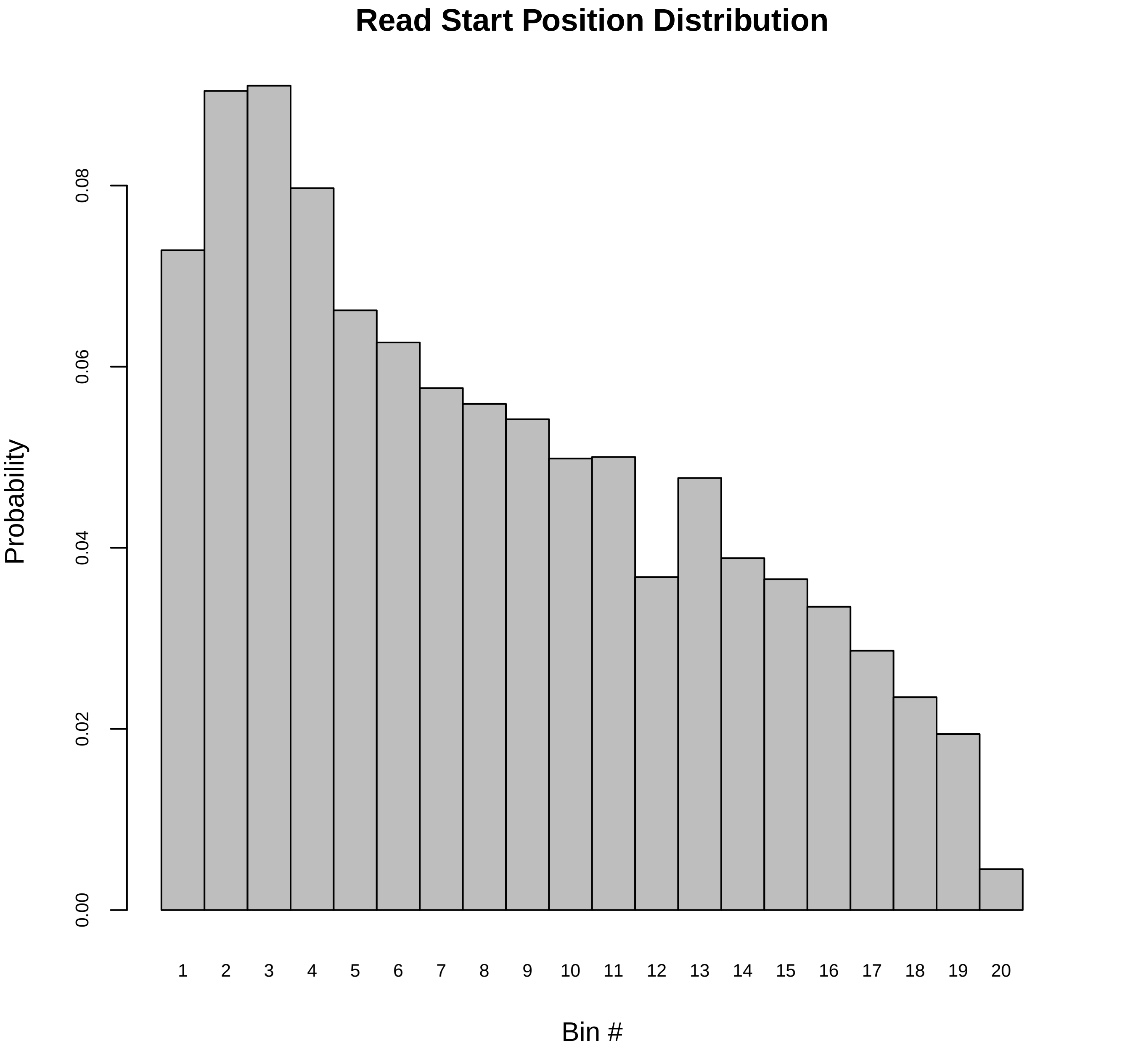
Read Start Position Distribution
Probability
0.00
0.02
0.04
0.06
0.08
1 2 3 4 5 6 7 8 9 10 11 12 13 14 15 16 17 18 19 20
Bin #

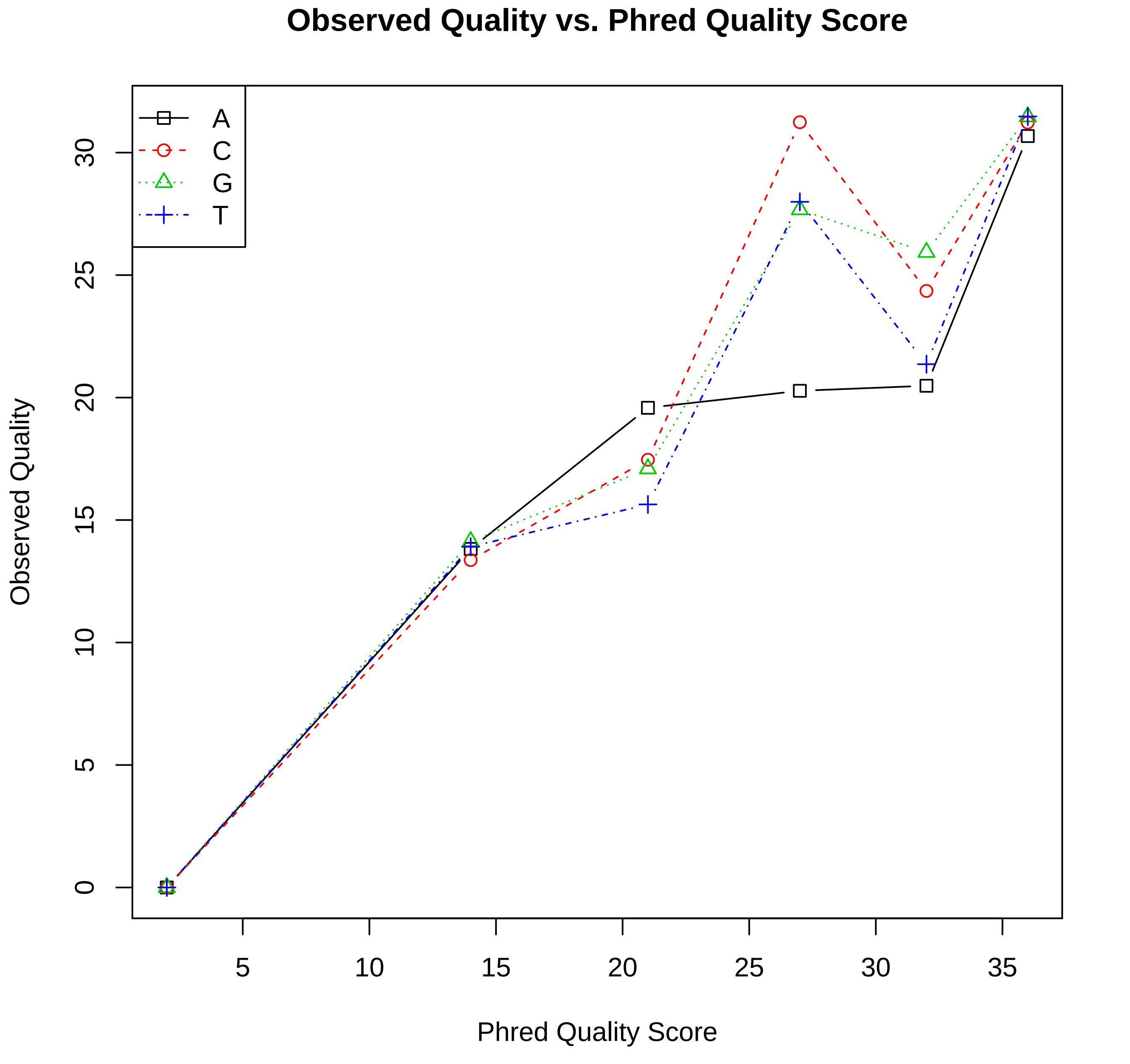

Observed Quality vs. Phred Quality Score
A
C
G
T
Observed Quality
Phred Quality Score
0
5
10
15
20
25
30
35

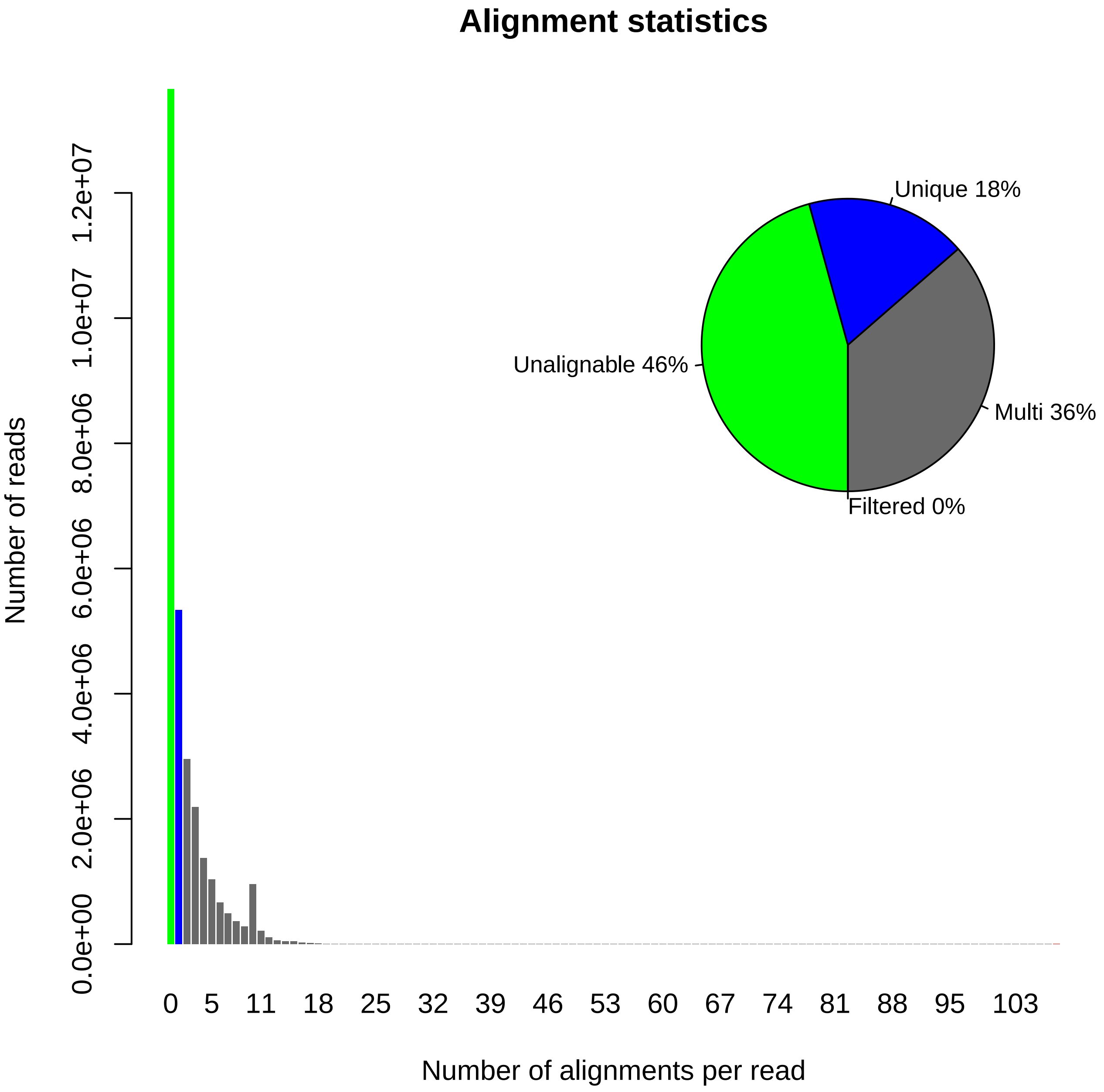

Alignment statistics
Number of reads
0.0e+00
2.0e+06
4.0e+06
6.0e+06
8.0e+06
1.0e+07
1.2e+07
0
5
11
18
25
32
39
46
53
60
67
74
81
88
95
103
Number of alignments per read
Unique 18%
Unalignable 46%
Multi 36%
Filtered 0%
