# Supplementary material for: Transcriptomic Changes in Cisplatin-Resistant MCF-7 Cells
Source: Int J Mol Sci. 2024 Mar 29;25(7):3820. doi: 10.3390/ijms25073820 (PMC11011657; doi:10.3390/ijms25073820)
Supplement: Supplementary file 1 [file ijms-25-03820-s001.zip › ijms-2687107-supplementary additions/WT-CMF-7 N3_star_trim_gcrh38_rsem.pdf]

Fragment Length Distribution

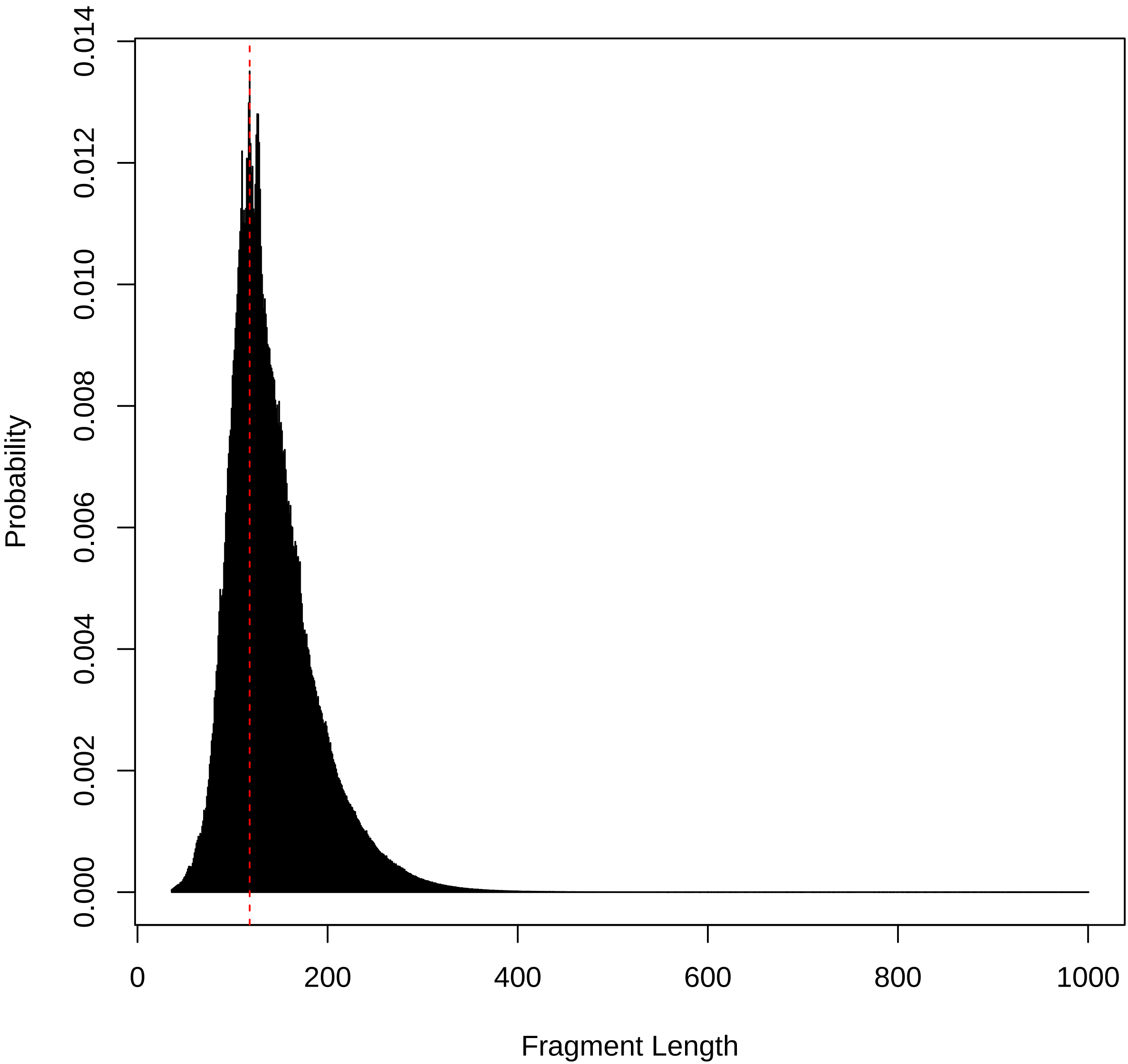

Mode = 118, Mean = 141.5, and Std = 46.1

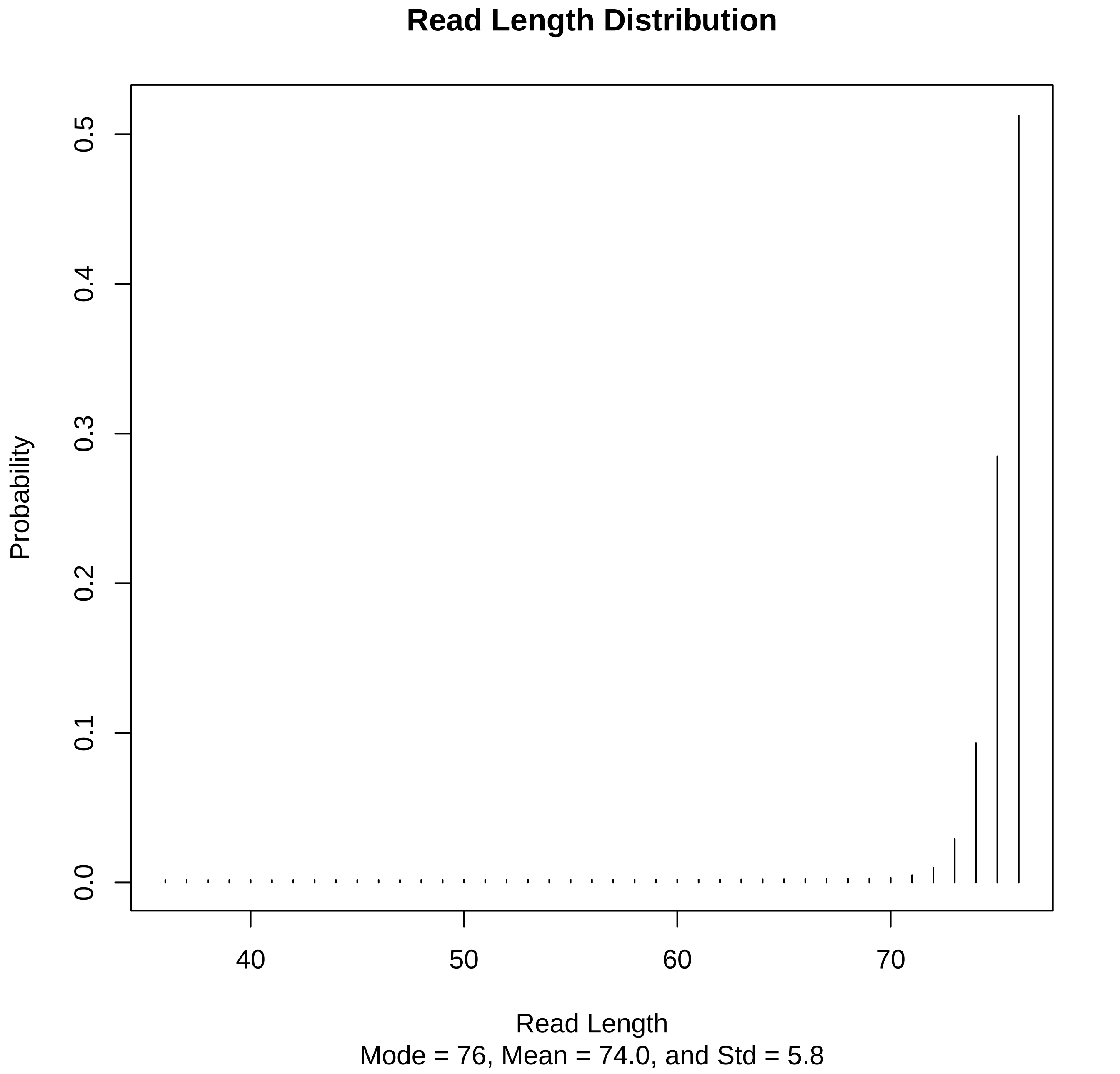

Read Length Distribution
Probability
0.0
0.1
0.2
0.3
0.4
0.5
40
50
60
70
Read Length
Mode = 76, Mean = 74.0, and Std = 5.8

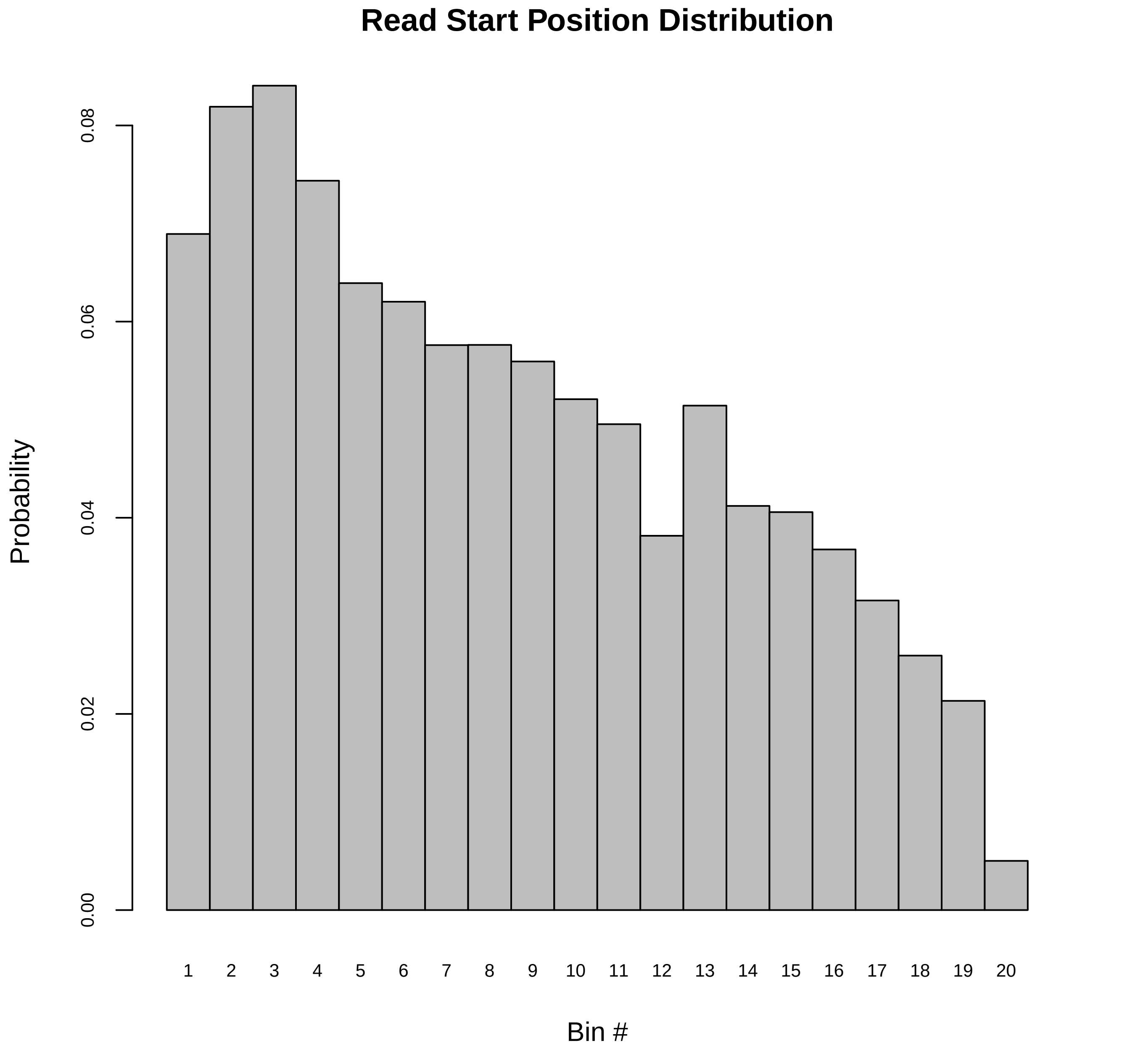
Read Start Position Distribution
Probability
0.00
0.02
0.04
0.06
0.08
1
2
3
4
5
6
7
8
9
10
11
12
13
14
15
16
17
18
19
20
Bin #

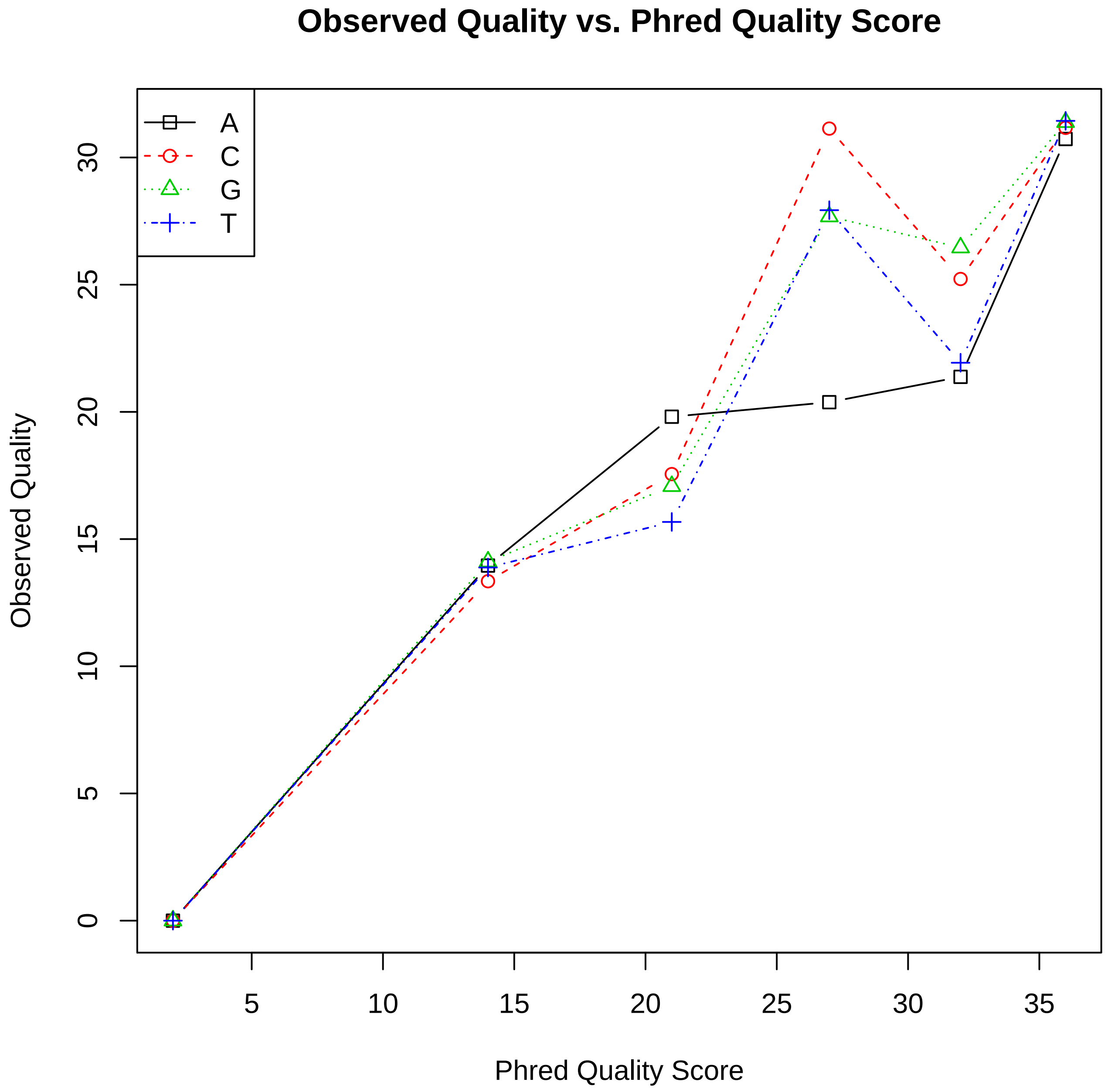

Observed Quality vs. Phred Quality Score
A
C
G
T
0
5
10
15
20
25
30
35
Phred Quality Score
Observed Quality

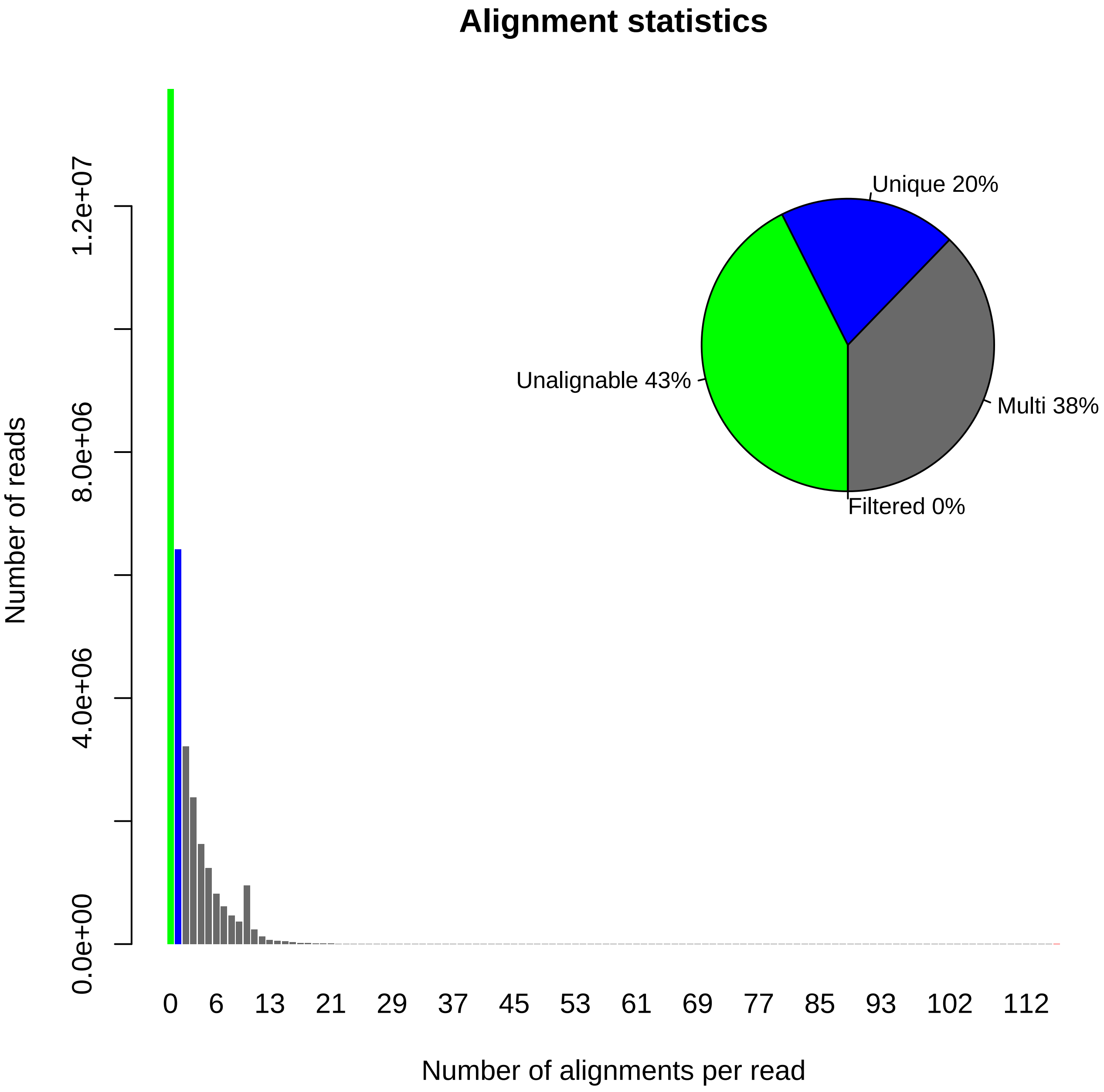

Alignment statistics
Number of reads
0.0e+00
4.0e+06
8.0e+06
1.2e+07
0
6
13
21
29
37
45
53
61
69
77
85
93
102
112
Number of alignments per read
Unique 20%
Unalignable 43%
Multi 38%
Filtered 0%
