# Supplementary material for: Transcriptomic Changes in Cisplatin-Resistant MCF-7 Cells
Source: Int J Mol Sci. 2024 Mar 29;25(7):3820. doi: 10.3390/ijms25073820 (PMC11011657; doi:10.3390/ijms25073820)
Supplement: Supplementary file 1 [file ijms-25-03820-s001.zip › Supplementary Figure S1.pdf]

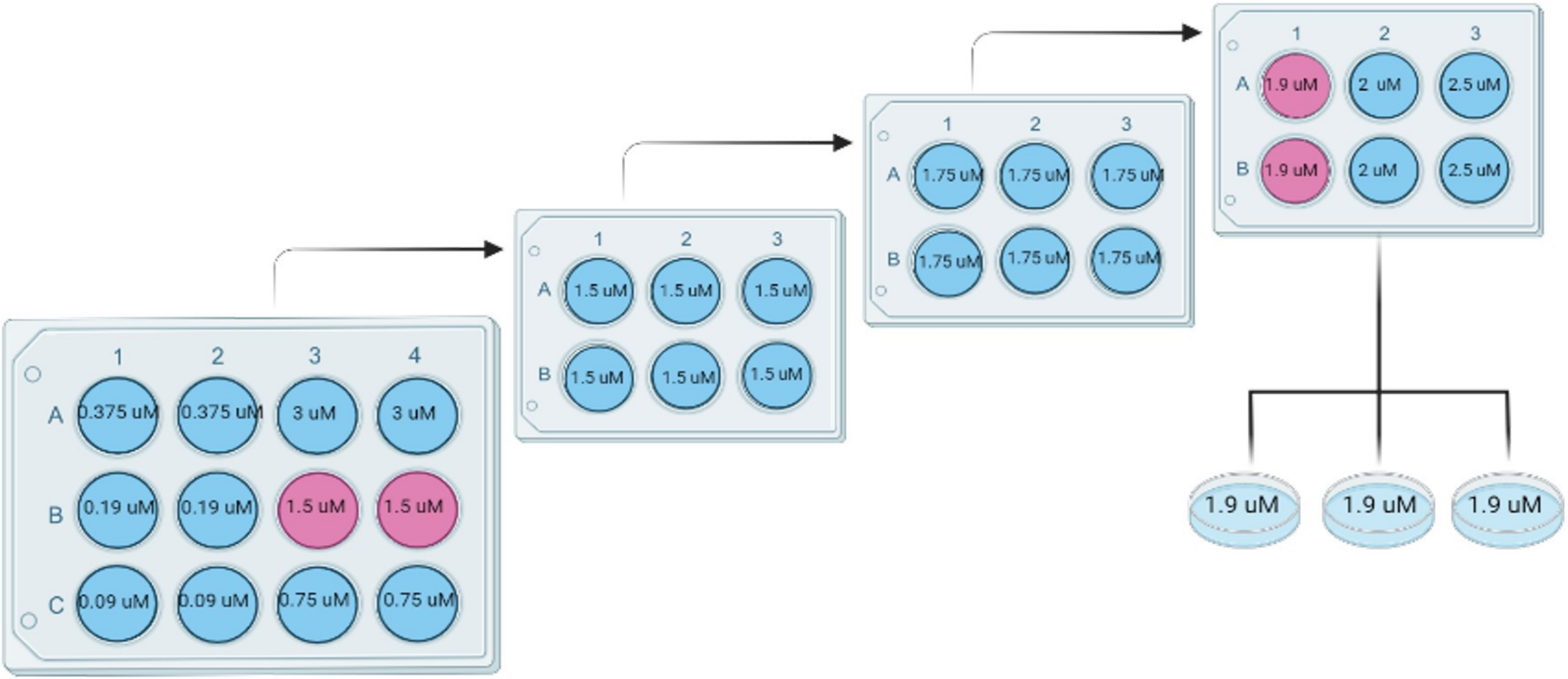

Supplementary Figure S1. **Generation of cisplatin-resistant breast cancer cells**. MCF7 cells were sequentially exposed to increasing concentrations of cisplatin. Initially, the cells were exposed to six concentrations of cisplatin ranging from 0.375 µM to 3 µM. The maximum tolerated concentration with viable cells was found to be 1.5 µM. Subsequently, the cells were exposed to 1.75 µM and then to 1.9 µM, and still, viable and proliferating cells were observed. However, higher concentrations of cisplatin were lethal for the cells.
